# EHMT1 knockdown induces apoptosis and cell cycle arrest in lung cancer cells by increasing CDKN1A expression

**DOI:** 10.1002/1878-0261.13050

**Published:** 2021-07-16

**Authors:** Jinkwon Lee, Kwangho Kim, Tae Young Ryu, Cho‐Rok Jung, Moo‐Seung Lee, Jung Hwa Lim, Kunhyang Park, Dae‐Soo Kim, Mi‐Young Son, Ryuji Hamamoto, Hyun‐Soo Cho

**Affiliations:** ^1^ Korea Research Institute of Bioscience and Biotechnology Daejeon Korea; ^2^ Department of Functional Genomics Korea University of Science and Technology Daejeon Korea; ^3^ Division of Molecular Modification and Cancer Biology National Cancer Center Tokyo Japan

**Keywords:** apoptosis, CDKN1A, cell cycle, EHMT1, lung cancer

## Abstract

Dozens of histone methyltransferases have been identified and biochemically characterized, but the pathological roles of their dysfunction in human diseases such as cancer remain largely unclear. Here, we demonstrate the involvement of EHMT1, a histone lysine methyltransferase, in lung cancer. Immunohistochemical analysis indicated that the expression levels of EHMT1 are significantly elevated in human lung carcinomas compared with non‐neoplastic lung tissues. Through gene ontology analysis of RNA‐seq results, we showed that EHMT1 is clearly associated with apoptosis and the cell cycle process. Moreover, FACS analysis and cell growth assays showed that knockdown of EHMT1 induced apoptosis and G1 cell cycle arrest via upregulation of CDKN1A in A549 and H1299 cell lines. Finally, in 3D spheroid culture, compared to control cells, EHMT1 knockdown cells exhibited reduced aggregation of 3D spheroids and clear upregulation of CDKN1A and downregulation of E‐cadherin. Therefore, the results of the present study suggest that EHMT1 plays a critical role in the regulation of cancer cell apoptosis and the cell cycle by modulating CDKN1A expression. Further functional analyses of EHMT1 in the context of human tumorigenesis may aid in the development of novel therapeutic strategies for cancer.

## Graphical abstract

Although histone methyltransferases have been previously well characterized, their role in carcinogenesis remains underexplored. In our study, we detected the overexpression of the histone lysine methyltransferase EHMT1 in lung cancer. EHMT1 modulated the gene expression of CDKN1A by regulating H3K9 dimethylation. Knockdown of EHMT1 in lung cancer cell lines upregulated CDKN1A expression and induced both apoptosis and cell cycle arrest. Our findings suggest that EHMT1 may potentially serve as a therapeutic target for the treatment of patients with lung cancer.

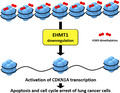

**Abbreviations**
CCK‐8: Cell Counting Kit‐8
CDH1: cadherin‐1
CDKN1A: cyclin‐dependent kinase inhibitor 1A
ChIP: chromatin immunoprecipitation
CV: crystal violet
DAVID: Database for Annotation
EHMT1: euchromatic histone lysine methyltransferase 1
FACS: fluorescence‐activated cell sorting
GLP: G9a‐like protein
GO: Gene Ontology
ULA: ultra‐low‐attachment

## 1 Introduction

Histone methylation plays important dynamic roles in regulating chromatin structure. Precise coordination and organization of open and closed chromatin are crucial for normal cellular processes such as DNA replication, repair, recombination, and transcription. Histone lysine methylation is considered to exert positive or negative regulatory effects on transcription depending on the methylation sites and status [1]. Despite a large body of information on the prominent role of histone lysine methyltransferases in transcriptional regulation, their involvement in human disease remains unclear.

EHMT1, also known as GLP, is mainly responsible for the monomethylation and dimethylation of histone H3 lysine 9 (H3K9), forming a heteromeric complex with G9a in euchromatin. Although G9a and EHMT1 can methylate H3K9 independently *in vitro*, the heteromeric complex appears to be essential for the methylation of euchromatin [2, 3]. Several studies have indicated that methylation mediated by the G9a‐EHMT1 complex is involved in transcriptional silencing, chromatin remodeling, and DNA methylation events, such as those affecting Mage‐A2, in G9a and GLP knockout embryonic stem (ES) cells [2, 4, 5]. EHMT1 knockout mice show very early embryonic lethality and a substantial reduction in H3K9 mono‐ and dimethylation as well as HP1 relocalization [3, 6]. Moreover, loss‐of‐function mutations in EHMT1 are related to 9p34 subtelomeric deletion syndrome [7, 8, 9]. Evidence of an association with breast cancer has been found for variants of *EHMT1* [10], although the physiological functions of this gene in cancer cells have not been elucidated.

Here, we demonstrate the possible involvement of EHMT1 in human tumorigenesis via direct regulation of CDKN1A by EHMT1. In a 3D spheroid culture system, EHMT1 knockdown interrupted 3D spheroid formation by upregulating *CDKN1A* expression. Therefore, EHMT1 could become a diagnostic and therapeutic marker for lung cancers, and our findings may contribute to the development of novel approaches for the treatment of cancers that overexpress EHMT1.

## 2 Materials and methods

### 2.1 Cell culture and reagents

The human lung cancer cell lines H1299 and A549 were purchased from the Korean Cell Line Bank (Seoul, South Korea) and cultured in RPMI‐1640 medium supplemented with 10% fetal bovine serum (FBS) and 1% penicillin/streptomycin in a humidified atmosphere with 5% CO_2_ at 37 °C.

### 2.2 siRNA transfection

siRNA duplexes targeting EHMT1 (siEHMT1: 5'‐CUCAGAACCAGUGCUACAU‐3' and 5'‐AUGUAGCACUGGUUCUGAG‐3') were purchased from Bioneer (Daejeon, Korea), and siRNA duplexes targeting CDKN1A (siCDKN1A: 5'‐CUGUACUGUUCUGUGUCUU‐3' and 5'‐AAGACACAGAACAGUACAG‐3') were purchased from Bioneer. Negative control siRNA (siCont: 5'‐AUGAACGUGAAUUGCUCAA‐3' and 5'‐UUGAGCAAUUCACGUUCAU‐3') was used as the control. siRNAs (50–100 nm) were transfected into the cancer cell lines for 48 h using RNAiMax (Thermo Fisher Scientific, Inc., Waltham, MA, USA).

### 2.3 Cell viability assay

Cell Counting Kit‐8 (CCK‐8: Dojindo Laboratories, Rockville, MD, USA) assay was used to assess cell viability. Cells were seeded in 6‐well plates starting at 2 × 10^5^ cells per well and incubated for 24 h. After siRNA transfection for 48 h, a mixture of CCK‐8 solution and RPMI‐1640 medium supplemented with 10% FBS was added to each well and incubated in 5% CO_2_ at 37 °C for 10 min. The absorbance at 450 nm was assessed by a microplate reader. For crystal violet staining, the cells were fixed with cold 100% methanol for 5 min at −20 °C and stained with 0.1% crystal violet solution (C0775, Sigma‐Aldrich, Merck KGaA, Darmstadt, Germany) for 5 min at room temperature after siRNA transfection for 48 h. Then, the cells were observed under a microscope (CELENA^®^ S Digital Imaging System, Logos Biosystems, Anyang, Korea).

### 2.4 FACS analysis

To measure caspase‐3/7 activity, we used the Muse Caspase‐3/7 Kit (MCH100108, Luminex, Austin, TX, USA). According to the user guide, cells were treated with siRNA for 48 h, and Muse Caspase‐3/7 working solution was then added. The cells were incubated for 30 min in a 37 °C incubator with 5% CO_2_. After incubation, approximately 5 × 10^4^ cells were analyzed with the Muse Cell Analyzer (Millipore, Burlington, MA, USA). To measure the apoptotic cell portion, the cells were collected after transfection with siRNA for 48 h and incubated with Muse Annexin V & Dead Cell Kit reagent (MCH100105, Millipore) for 20 min at room temperature. After incubation, approximately 5 × 10^4^ cells were analyzed by the Muse Cell Analyzer (Millipore). To identify shifts in the proportion of cell cycles, approximately 1 × 10^4^ cells were treated with siRNA for 48 h, fixed overnight, and incubated with Muse Cell Cycle Kit reagent (MCH100106, Luminex) for 20 min in accordance with the instruction manual. After incubation, the cells were analyzed by the Muse Cell Analyzer (Millipore). The FACS data obtained were analyzed by using muse 1.5 Analysis software (Millipore).

### 2.5 Immunohistochemical staining

An EnVision^+^ kit/HRP kit (Dako, Carpinteria, CA, USA) was used. Paraffin‐embedded sections of lung tumor specimens were processed in a microwave (90 °C) with antigen retrieval solution (pH 9) (S2367; Dako), treated with a peroxidase‐blocking reagent, and then treated with a protein‐blocking reagent (K130, X0909; Dako). Tissue sections were incubated first with a rabbit anti‐EHMT1 antibody (BIO MATRIX RESEARCH, Chiba, Japan) and then with an HRP‐conjugated secondary antibody (Dako). Immunoreactivity was visualized with a chromogenic substrate (Liquid DAB Chromogen; Dako). Finally, tissue specimens were stained with Mayer’s hematoxylin solution (Hematoxylin QS; Vector Laboratories, Burlingame, CA, USA) for 20 s to distinguish nuclei from the cytoplasm. Human lung cancer tissues were purchased from BioChain [11].

### 2.6 Quantitative real‐time PCR (qRT‐PCR)

Total RNA was isolated from the indicated cell lines using the Qiagen RNeasy Mini Kit (Qiagen, Hilden, Germany) according to the manufacturer’s instructions. RNA aliquots of 1 µg were then reverse‐transcribed by the iScript™ cDNA Synthesis Kit (Bio‐Rad Laboratories, Inc., Hercules, CA, USA) according to the standard protocol. For qRT‐PCR, PCR was performed with the SensiFAST SYBR Lo‐ROX Kit (Meridian Bioscience Inc., Cincinnati, OH, USA) following the manufacturer’s instructions. qRT‐PCR was performed on cDNA samples by Brilliant III Ultra‐Fast SYBR^®^ Green QPCR Master Mix (Agilent Technologies), and signals were detected by the AriaMx Real‐Time PCR System (Agilent Technologies). The threshold value for fluorescence was calculated by agilent aria 1.6 software (Agilent, Santa Clara, CA, USA). The PCR primers used were as follows: EHMT1 (forward, 5′‐CAGGACTTCCAAGGAGAGCA‐3′ and reverse, 5′‐ACTCAGGTCAGACTCGTCAC‐3′), CDKN1A (forward, 5′‐GAGTGGGGGCATC ATCAAAA‐3′ and reverse, 5′‐CTAGGCTGTGCTCACTTCAG‐3′), CDH1 (forward, 5′‐CGAGAGCTACACGTTCACGG‐3′ and reverse, 5′‐GGGTGTCGAGGGAAAAA TAGG‐3′), and ACTB (forward, 5′‐ACTCTTCCAGCCTTCCTTCC‐3′ and reverse, 5′‐CAATGCCAGGGTACATGGTG‐3′).

### 2.7 Western blot analysis

The cells were washed once with phosphate‐buffered saline (PBS) and then lysed in cold lysis buffer [50 mm Tris/HCl (pH 7.4), 150 mm NaCl, 1% Triton X‐100, 0.1% SDS, 1 mm EDTA, 1 mm Na3VO4, 1 mm NaF, and 1X protease inhibitor cocktail). Cell lysates were centrifuged at 14 000 ***g*** for 15 min at 4 °C and boiled in 5X sample buffer after protein concentration assessment with a BSA kit (cat. no. 23208, Thermo Fisher Scientific, Inc.). Following protein sampling, nitrocellulose membranes (cat. no. 1620145, Bio‐Rad Laboratories), blocking reagent (5% skim milk, 1 h at room temperature), and a 4–20% precast gel (cat. no. 456‐1094, Bio‐Rad Laboratories) were used for western blot analysis with the following antibodies at dilutions of 1 : 1000: anti‐EHMT1 (A301‐642A, Bethyl Laboratories, Inc., Montgomery, TX, USA); anti‐PARP (cat. no. 9542, Cell Signaling Technology, Inc.); and anti‐p21 (sc‐6246) and anti‐β‐actin (sc‐47778) from Santa Cruz Biotechnology, Inc. (Santa Cruz, CA, USA). Final incubation with secondary antibodies (rabbit: SC‐2357, mouse: SC‐2031, Santa Cruz Biotechnology, Inc.) was conducted at room temperature for 1 h, and ECL solution (cat. no. 170‐5060, Bio‐Rad Laboratories) was used for visualization. A chemiluminescence imaging system (Mini HD9, Uvitec, Cambridge, UK) was used for imaging.

### 2.8 Chromatin immunoprecipitation (ChIP)

ChIP was performed with SimpleChIP Plus Sonication Chromatin IP Kit (Cell Signaling Technology, Inc.) following the manufacturer’s instructions. A549 cells were transfected with siCont and siEHMT1 for 48 h, crosslinked with 1% formaldehyde (F8775, Sigma‐Aldrich) for 10 min at room temperature, and quenched with 1X glycine (Cell Signaling Technology, Inc.) for 5 min at room temperature. Subsequently, the cells were washed with cold 1X PBS containing 1X Protease inhibitor Cocktail II (Cell Signaling Technology, Inc.). After nuclear extraction, the chromatin solution was sonicated with a Bioruptor^®^ Pico sonication device (B01060010, Diagenode, Liège, Belgium) for 20 cycles: 30 s ON followed by 30 s OFF, to obtain chromatin fragments of 200–1000 bp. The sheared chromatin mixtures were incubated with 2 μg of H3K9me2 antibody (ab1220; Abcam) overnight at 4 °C with rotation. ChIP‐Grade Protein G Magnetic Beads were then added to the mixtures, and rotation for 2 h at 4 °C was followed. According to the user guide, low salt and high salt wash were prepared and used for wash steps. The mixtures were incubated with 1X ChIP elution buffer and RNase A for 30 min at 37 °C, followed by incubation with proteinase K for 2 h at 62 °C. After purification with DNA Purification Buffers and Spin Columns (Cell Signaling Technology, Inc.), the samples were analyzed by semiquantitative RT‐PCR with CDKN1A primers. The primers were as follows: CDKN1A‐P1 (forward, 5′‐GGGCACATTTAGACATAGCAGG‐3′ and reverse, 5′‐CTCTATGAGAGTCCTTGTGGGC‐3′) and CDKN1A‐P2 (forward, 5′‐CCACCTG AATACCTGGGACTAC‐3′ and reverse, 5′‐GGTGAAACCCCGTCTCCATTA‐3′).

### 2.9 Immunocytochemistry

Cultured cells were fixed in 4% paraformaldehyde at room temperature for 10 min, permeabilized with 0.5% Triton X‐100 (Sigma‐Aldrich) in PBS for 10 min, and blocked with 5% bovine serum albumin in PBS for 30 min. Fixed cells were incubated with anti‐p21 antibody (sc‐6246, Santa Cruz Biotechnology, Inc.) overnight at 4 °C and stained with an Alexa Fluor‐conjugated secondary antibody (Life Technologies). Fluorescent images were obtained by using the CELENA^®^ S Digital Imaging System (Logos Biosystems).

### 2.10 3D cell culture

To perform spheroid culture of lung cancer cell lines, ultra‐low‐attachment microplates were used (Corning, Cat. 7007; Corning, NY, USA). Gene knockdown was performed by siRNA transfection; after transfection, cells were seeded into plates at concentrations of 1 × 10^5^ and 1.2 × 10^5^ cells per well for the H1299 and A549 cell lines, respectively. The spheroids were cultured for 3 days and observed under a microscope (CKX53, Olympus Corporation, Tokyo, Japan).

## 3 Results

### 3.1 EHMT1 is overexpressed in lung cancer tissues

**Fig. 1:**
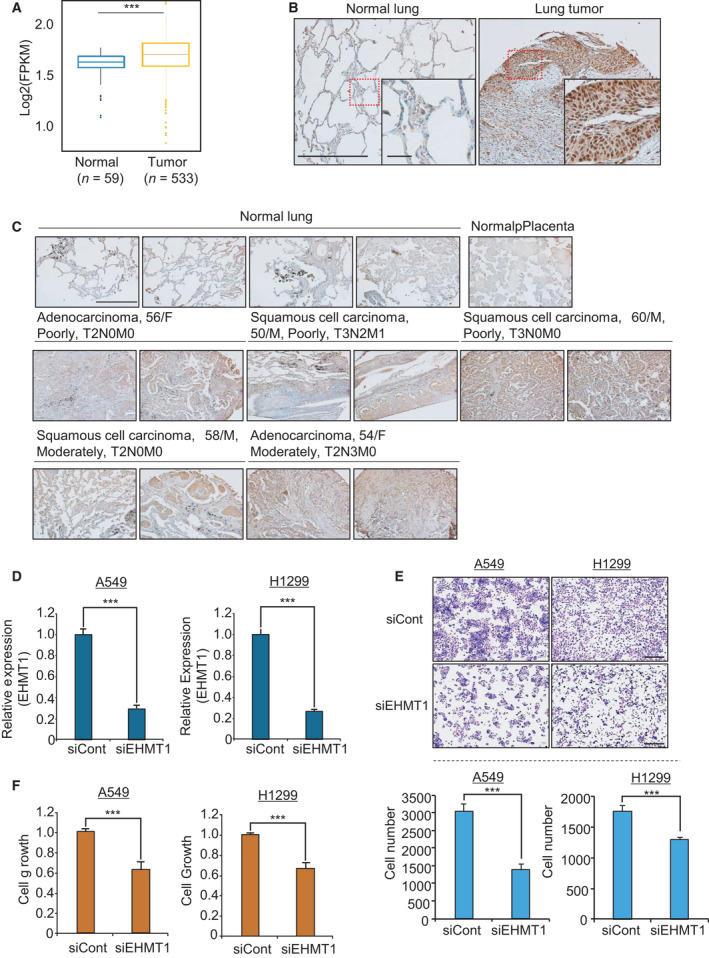
EHMT1 is overexpressed in lung cancer tissues. (A) Expression levels of *EHMT1* in normal and lung cancer samples derived from the TCGA database. *P* values were calculated using Student’s *t*‐tests (***P < 0.001). (B) and (C) Immunohistochemical analysis of EHMT1. Lung cancer tissues were purchased from BioChain (https://www.biochain.com). Scale bar, 500 μm. Scale bar, 50 μm in magnified figure (B). (D) qRT‐PCR analysis of *EHMT1* expression after transfection of cells with siEHMT1. *P* values were calculated using Student’s *t*‐tests (***P < 0.001). (E) Cell growth assay after transfection with siEHMT1 and siCont for 48 h. A549 and H1299 cells were fixed in 100% methanol and stained with CV solution. Scale bar, 500 μm (upper). Quantification of cell numbers in CV staining assay. The *P* values were calculated using Student’s *t*‐tests (***P < 0.001) (below). (F) CCK‐8 solution was added to the culture medium, and the cells were incubated for 5 min at 37 °C. Cell growth was assessed by detecting the absorbance with a microplate reader (450 nm). Mean ± SD of three independent experiments. *P* values were calculated using Student’s *t*‐test (***P < 0.001).

To assess EHMT1 expression levels, we performed tissue‐wide expression profile analysis of EHMT1 in the Gene Expression Database of Normal and Tumor Tissues (GENT) (http://gent2.appex.kr/gent2/) and showed that the expression of EHMT1 was higher in several types of cancers than in normal tissues (Fig. S1). Additionally, EHMT1 was significantly overexpressed in lung cancer samples (*n* = 533) compared to normal lung samples (*n* = 59) according to RNA sequencing (RNA‐seq) data derived from The Cancer Genome Atlas (TCGA) portal (Fig. 1A). In addition, to verify the overexpression of EHMT1 in lung cancer, we performed immunohistochemical analysis with a tissue microarray of lung cancer and normal lung tissues. As shown in Fig. 1B,C, we clearly observed the nuclear staining pattern in lung cancer tissue, and EHMT1 was significantly overexpressed in lung cancer tissues compared with normal lung and placental tissues. Taken together, these results indicate that EHMT1 is significantly overexpressed in lung cancer at both transcript and protein levels. Thus, we suggest that EHMT1 overexpression is closely associated with lung cancer proliferation.

### 3.2 EHMT1 knockdown suppressed proliferation in lung cancer cell lines

**Fig. 2:**
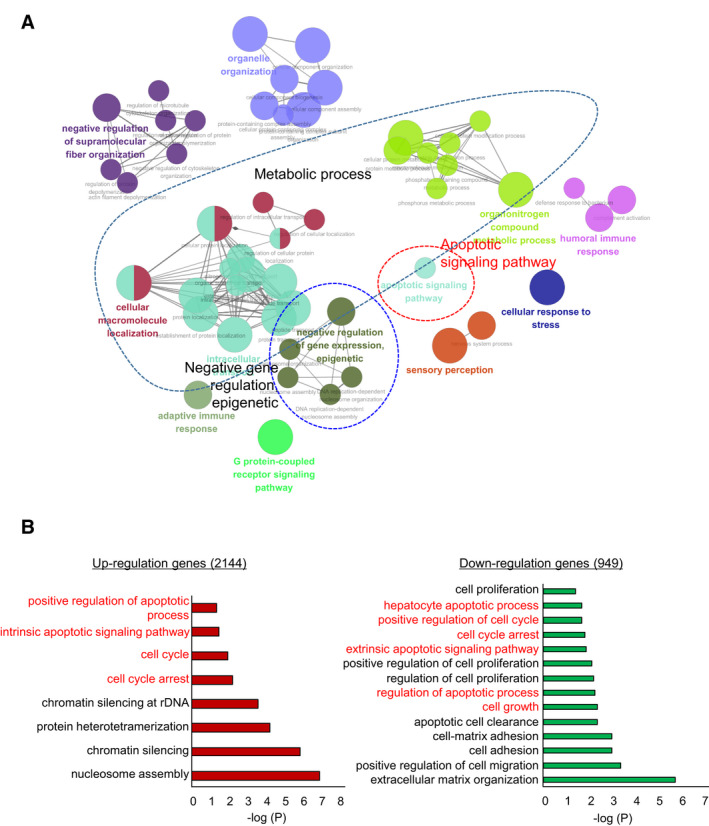
EHMT1 knockdown‐related cell apoptosis and cell cycle arrest terms in GO analysis. (A) GO pathway term enrichment networks. GO pathway term networks in the EHMT1 knockdown and control groups were functionally grouped by ClueGO. The cutoff value was set at *P* value > 0.05. (B) DAVID‐based GO analysis of the RNA‐seq results, including 3093 DEGs (david ver. 6.8).

We applied siEHMT1 and siCont to cells and confirmed the knockdown of EHMT1 (Fig. 1D); next, we assessed cell growth by CCK‐8 assay and crystal violet (CV) staining. The A549 and H1299 cell lines were treated with siEHMT1, and the growth of these lung cancer cell lines was suppressed by EHMT1 knockdown compared with siCont transfection (Fig. 1E,F). Next, to verify the function of EHMT1 in lung cancer, we performed RNA‐seq analysis of siEHMT1‐ versus siCont‐transfected H1299 cells. A total of 3093 dysregulated genes (2144 upregulated genes and 949 downregulated genes) (cutoff, 1.5‐fold; *P* > 0.05) were identified (Table S1). We performed gene ontology (GO) term analysis of these differentially expressed genes (DEGs) using the ClueGO program in Cytoscape and found enrichment of apoptotic‐related terms and metabolic process terms with EHMT1 knockdown (Fig. 2A). Moreover, using the Database for Annotation, Visualization and Integrated Discovery (david) version 6.8 (https://david.ncifcrf.gov/), we carried out GO term analysis of the genes that were up‐ and downregulated by EHMT1 knockdown. The 949 downregulated genes were enriched for cell cycle‐related terms (positive regulation of cell cycle, cell cycle arrest) and apoptosis‐related terms (apoptotic process, regulation of apoptotic process). In addition, the 2144 upregulated genes also exhibited enrichment in cell cycle‐ and apoptosis‐related terms (Fig. 2B). Thus, we suggest that the function of EHMT1 is deeply related to the process of cell apoptosis and cell cycle in the development of lung cancer.

### 3.3 EHMT1 knockdown induced cell apoptosis and cell cycle arrest

**Fig. 3:**
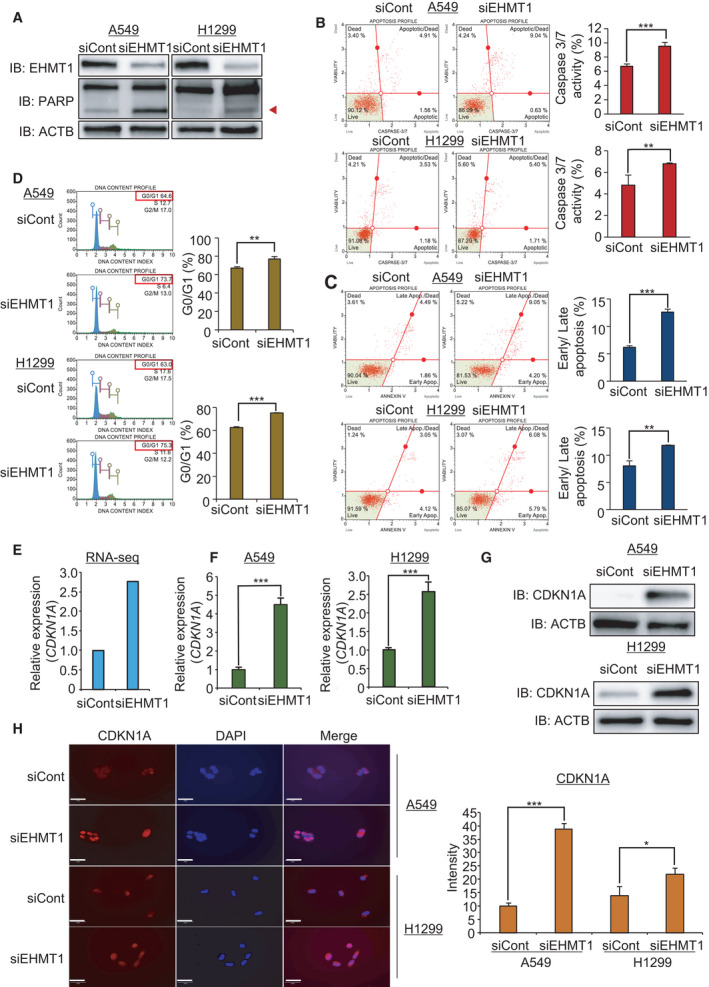
EHMT1 knockdown induces cell apoptosis and cell cycle arrest in A549 and H1299 cell lines. (A) Western blot analysis after siEHMT1 transfection using anti‐PARP and anti‐ACTB antibodies. ACTB was used as the internal control in A549 and H1299 cells. (Arrowhead: cleaved PARP) (B) FACS analysis using Muse Caspase‐3/7 working solution was performed after cells were transfected with siEHMT1 and siCont. The upper right panel indicates the apoptotic and dead cell proportions (left). The quantification of caspase‐3/7 activity. Mean ± SD of three independent experiments. The *P* values were calculated using Student’s *t*‐tests (***P < 0.001 and **P < 0.01) (right). (C) FACS analysis of Annexin V staining was carried out after cells were transfected with siEHMT1 and siCont. The lower right and upper right quadrants indicate early apoptosis and late apoptosis, respectively (left). The quantification of apoptosis. Mean ± SD of three independent experiments. The *P* values were calculated using Student’s *t*‐tests (***P < 0.001 and **P < 0.01) (right). (D) FACS analysis using propidium iodide after cells were transfected with siEHMT1 and siCont (left). The quantification of cell cycle. Mean ± SD of three independent experiments. The *P* values were calculated using Student’s *t*‐tests (**P < 0.01 and ***P < 0.001) (right). (E) RNA‐seq result of *CDKN1A* by EHMT1 knockdown. (F) qRT‐PCR analysis of *CDKN1A* expression after transfection of siEHMT1. Mean ± SD of three independent experiments. *P* values were calculated using Student’s *t*‐tests (***P < 0.001). (G) Western blot analysis after siEHMT1 transfection using anti‐CDKN1A and anti‐ACTB antibodies. ACTB was used as the internal control in A549 and H1299 cells. (H) Immunocytochemical analysis of CDKN1A. A549 and H1299 cells treated with siEHMT1 and siCont were fixed with 100% methanol and stained with anti‐CDKN1A (Alexa Fluor 568, red) and DAPI (blue). Scale bar, 50 μm (left). Quantification of CDKN1A expression in immunocytochemical analysis. Mean ± SD of three independent experiments. The *P* values were calculated using Student’s *t*‐tests (***P < 0.001 and *P < 0.05) (right).

To validate whether the suppression of cell growth by EHMT1 knockdown was related to apoptosis, we performed western blot analysis using an anti‐PARP antibody. In Fig. 3A, the level of cleaved PARP was significantly increased by EHMT1 knockdown. Moreover, the caspase‐3/7 activity assay revealed that the activity of caspase‐3/7 was increased by EHMT1 knockdown compared with siCont transfection according to FACS analysis (Fig. 3B). Additionally, Annexin V staining showed that the rate of early and late apoptosis was increased in the EHMT1 knockdown groups compared to the siCont groups (Fig. 3C).

Next, to assess the effect of EHMT1 on the cell cycle process, we performed FACS analysis in A549 and H1299 cell lines after transfection with siEHMT1. Figure 3D shows that G1 arrest was induced by EHMT1 knockdown in both cell lines compared to siCont. Taken together, these results indicate that EHMT1 is highly associated with the regulation of lung cancer proliferation. We hypothesized that EHMT1 plays an important role in lung cancer proliferation and could be a therapeutic target for lung cancer.

### 3.4 CDKN1A expression is regulated by EHMT1 in lung cancer cell lines

Next, we searched the RNA‐seq results for potential EHMT1 targets, and we selected *CDKN1A* as a candidate that was upregulated by EHMT1 knockdown (Fig. 3E). CDKN1A is associated with cell cycle regulation and cell apoptosis in several types of cancers. As a cyclin‐dependent kinase (CDK) inhibitor, CDKN1A plays an important role in the cell cycle, promoting cell cycle arrest and apoptosis in response to DNA damage. In cancer, CDKN1A acts as a tumor suppressor gene [12, 13]. Under conditions of DNA damage, upregulation of CDKN1A by p53 suppresses tumor cell proliferation [14]. Thus, we established the hypothesis that induction of CDKN1A expression by EHMT1 knockdown induces cell cycle arrest and apoptosis in lung cancer cells. To confirm the upregulation of CDKN1A after EHMT1 knockdown, we performed qRT‐PCR and western blot analysis after knockdown of EHMT1 in A549 and H1299 cell lines. As shown in Fig. 3F,G, we validated the upregulation of CDKN1A expression after EHMT1 knockdown in both cell lines. Moreover, in the immunocytochemical analysis, induction of CDKN1A expression by EHMT1 knockdown was observed in both cell lines (Fig. 3H). Taken together, these results demonstrated that EHMT1 knockdown increased CDKN1A expression in lung cancer cell lines and that upregulation of CDKN1A expression induced cell cycle arrest and apoptosis.

### 3.5 CDKN1A is a direct target of EHMT1 in lung cancer

**Fig. 4:**
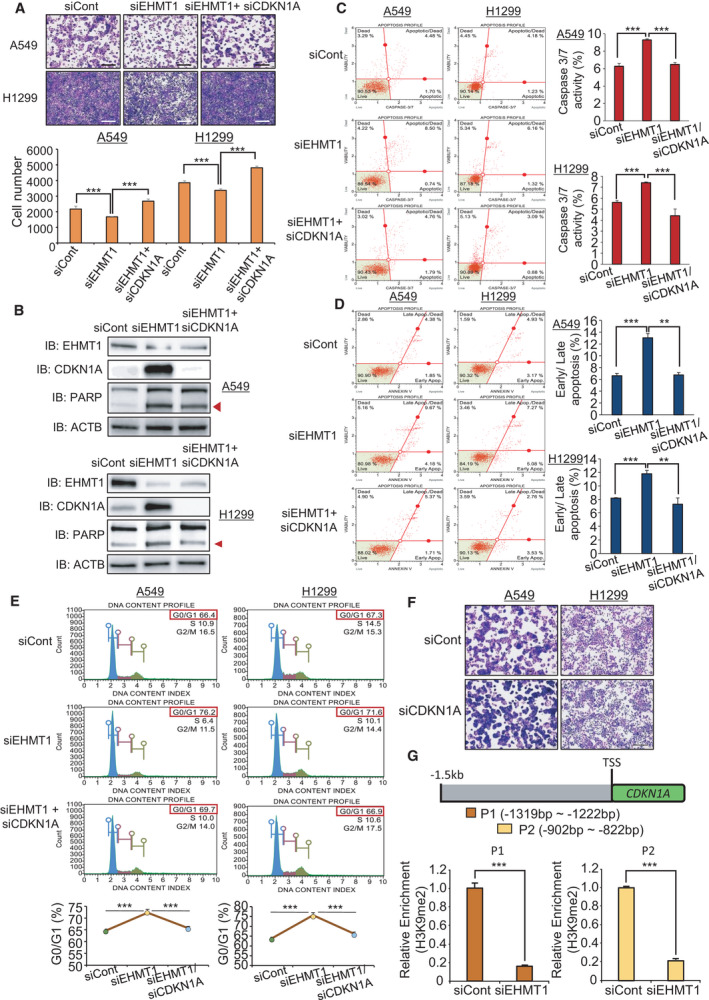
CDKN1A is a direct target of EHMT1 in lung cancer. (A) Cell growth assay after transfection of the cells with siEHMT1, siCont, and siEHMT1/siCDKN1A for 48 h. A549 and H1299 cells were fixed in 100% methanol and stained with CV solution. Scale bar, 500 μm (upper). Quantification of cell numbers in CV staining assay. Mean ± SD of three independent experiments. The *P* values were calculated using Student’s *t*‐tests (***P < 0.001) (below). (B) Western blot analysis after siEHMT1, siCont, and siEHMT1/siCDKN1A transfection using anti‐EHMT1, anti‐PARP, and anti‐ACTB antibodies. ACTB was used as the internal control in A549 and H1299 cells. (Arrowhead: cleaved PARP) (C) FACS analysis using Muse Caspase‐3/7 working solution was performed after transfection of siEHMT1, siCont, and siEHMT1/siCDKN1A. The upper right panel indicates the apoptotic and dead cell proportions (left). Quantification of caspase‐3/7 activity. Mean ± SD of three independent experiments. The *P* values were calculated using Student’s *t*‐tests (***P < 0.001) (right). (D) FACS analysis of Annexin V staining was carried out after transfection of the cells with siEHMT1, siCont, and siEHMT1/siCDKN1A. The lower right and upper right quadrants indicate early apoptosis and late apoptosis, respectively (left). Quantification of apoptosis. Mean ± SD of three independent experiments. The *P* values were calculated using Student’s *t*‐tests (***P < 0.001 and **P < 0.01) (right). (E) FACS analysis using PI staining was performed after transfection of A549 and H1299 cells with siEHMT1, siCont, and siEHMT1/siCDKN1A (upper). Quantification of cell cycle analysis. Mean ± SD of three independent experiments. The *P* values were calculated using Student’s *t*‐tests (***P < 0.001) (below). (F) Cell growth assay after transfection with siCDKN1A and siCont for 48 h. A549 and H1299 cells were fixed in 100% methanol and stained with CV solution. Scale bar, 500 μm. (G) Graphical abstract for ChIP primer design on the CDKN1A promoter region (upper). The ChIP assay was performed with anti‐H3K9me2 antibody. The result is shown as a relative enrichment compared to the control in A549 cells after siEHMT1 transfection. Mean ± SD of three independent experiments. *P* values were calculated using Student’s *t*‐test (***P < 0.001).

Next, to validate the relationship between EHMT1 and CDKN1A, we performed recovery analysis by siEHMT1 and siCDKN1A cotransfection in A549 and H1299 cell lines. In the cell growth analysis, we clearly observed that the cotransfection with siCDKN1A attenuated the growth suppression mediated by EHMT1 knockdown (Fig. 4A). In the western blot analysis, the expression of cleaved PARP was reduced by cotransfection of EHMT1 and CDKN1A (Fig. 4B). In the FACS analysis, we found the same recovery patterns with CDKN1A knockdown. Caspase‐3/7 activity and Annexin V staining assays showed that the elevated caspase‐3/7 activity and apoptosis rates were decreased to the levels of the siCont group upon cotransfection (Fig. 4C,D). Moreover, cell cycle analysis revealed that G1 arrest by EHMT1 knockdown was attenuated by CDKN1A knockdown in lung cancer cell lines (Fig. 4E). However, the knockdown of CDKN1A alone did not affect cell growth in either cell line (Fig. 4F).

Next, to assess whether CDKN1A is a direct target for EHMT1, we performed a ChIP assay using a histone H3 lysine 9 dimethylation antibody after EHMT1 knockdown in A549 and H1299 cells. Figure 4G shows the ChIP primers for the promoter region of CDKN1A; our results showed that the status of H3K9 dimethylation was decreased in the promoter region of CDKN1A in EHMT1 knockdown cells compared to siCont cells (Fig. 4G). Thus, we suggest that downregulation of CDKN1A expression by epigenetic regulation of EHMT1 plays an important role in lung cancer proliferation.

### 3.6 EHMT1 knockdown reduced spheroid formation in 3D culture

**Fig. 5:**
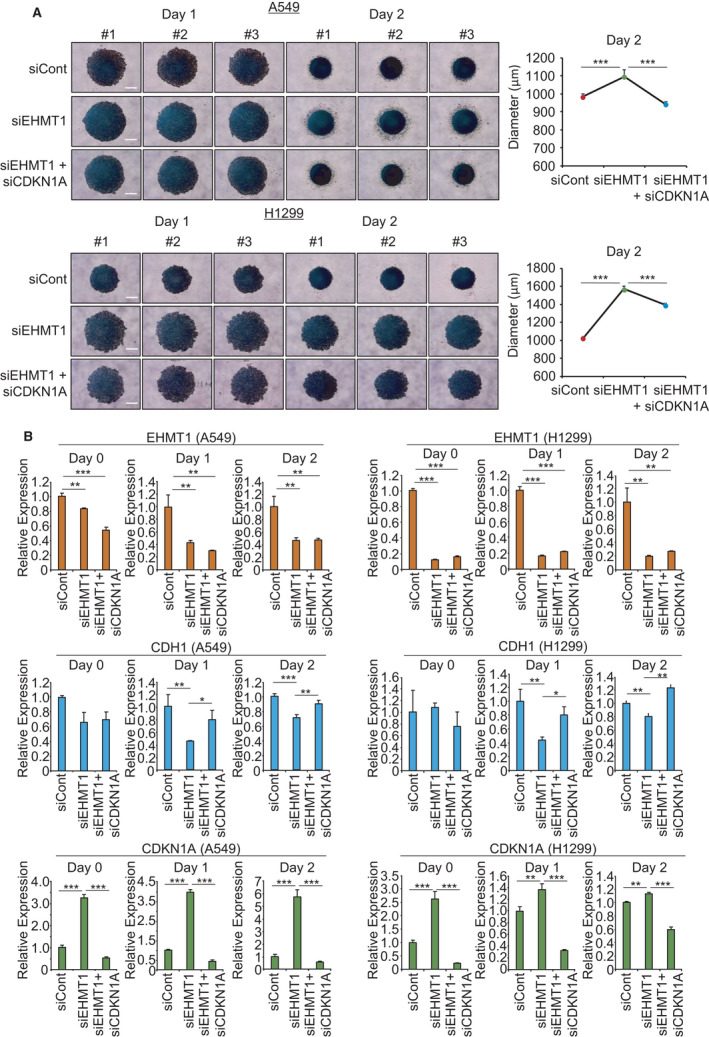
CDKN1A is regulated by EHMT1 in 3D spheroid culture system. (A) 3D spheroid formation assay. Cells transfected with siEHMT1, siCont, and siEHMT1/siCDKN1A were loaded onto ULA plates and incubated for 48 h. The cells were photographed under a microscope each day, Scale bar, 500 μm (left), and the size of spheroids was measured by the ImageView software program (right). Mean ± SD of three independent experiments. *P* values were calculated using Student’s *t*‐test (***P < 0.001). (B) qRT‐PCR analysis of *CDKN1A, CDH1, and EHMT1* expression after transfection with siEHMT1, siCont, and siEHMT1/siCDKN1A. Mean ± SD of three independent experiments. *P* values were calculated using Student’s *t*‐tests (**P < 0.01 and ***P < 0.001). ‘Day 0’ means the samples treated with siEHMT1 for 24 h.

The 3D culture system can reflect the structural complexity of cancers and the tumor environment. Thus, 3D culture systems, such as spheroid culture and patient‐derived cancer organoids, can be used as successful models for drug screening and target gene studies for cancer research [15, 16]. In this study, we constructed 3D spheroid models with lung cancer cell lines using an ultra‐low‐attachment (ULA) plate system and assessed spheroid formation and target interactions with an EHMT1 knockdown model. Figure 5A shows that the cells treated with siCont formed spheroids well in 3D culture, but the EHMT1 knockdown cells exhibited a loosening of this 3D formation. Moreover, in the qRT‐PCR analysis, the expression of E‐cadherin increased in siCont cells compared to knockdown cells, suggesting that the cells were well aggregated for spheroid formation. However, in EHMT1 knockdown samples, E‐cadherin expression was decreased in accordance with the decrease in spheroid formation. Additionally, as shown by 2D culture assays, CDKN1A expression was induced by EHMT1 knockdown (Fig. 5B). Next, to validate rescue by siCDKN1A, we cotransfected cells with siEHMT1 and siCDKN1A in 3D culture and found that the aggregation of spheroids was restored (Fig. 5A). Moreover, the expression level of E‐cadherin was restored by cotransfection (Fig. 5B). Thus, we suggest that dysregulation of EHMT1 is clearly associated with lung cancer proliferation via regulation of CDKN1A.

## 4 Discussion

We previously reported that dysregulation of several histone methyltransferases is associated with carcinogenesis [17, 18, 19, 20, 21, 22, 23] and that these histone methyltransferases and demethylases participate in cancer growth via the methylation of nonhistone proteins [24]. When complexed with G9a, EHMT1 is mainly responsible for monomethylation‐ and dimethylation‐related modifications of H3K9 in euchromatin regions [2, 3], and EHMT1 overexpression is associated with poor cancer‐specific survival in esophageal squamous cell cancer [25]. Additionally, EHMT1 directly regulates NF‐kB gene expression via H3K9 dimethylation [26].

Here, we confirmed that EHMT1 expression is upregulated in lung cancers via immunohistochemistry and that EHMT1 expression is upregulated in several types of cancers via analysis of GENT and RNA‐seq lung cancer data from the TCGA portal. In addition, we observed growth suppression in lung cancer cell lines upon knockdown of EHMT1 via FACS analysis of Annexin V staining and caspase‐3/7 activity. Moreover, we confirmed the effects of EHMT1 on lung cancer proliferation via GO analyses of RNA‐seq data and found that it is particularly involved in the cell cycle and cell apoptosis. Collectively, our findings indicate that dysregulation of EHMT1 may play an important role in the process of lung cancer cell growth, and suggest that EHMT1 may be a rational drug target in the treatment of lung cancer.

The results of the current study indicate that CDKN1A expression is epigenetically controlled by EHMT1 expression in lung cancer. Mono‐, di‐, and trimethylation of H3K9 are associated with the formation of the heterochromatin structure and with the transcriptional repression of activated genes. Thus, the direct targets of EHMT1 were upregulated upon EHMT1 knockdown. Herein, we found that EHMT1 knockdown upregulated CDKN1A expression and confirmed the reduction in H3K9 dimethylation at the promoter region of *CDKN1A*.

3D culture models have been recognized as alternative methods for anticancer drug development that mimic the physiological status and tumor formation process of cancer [15, 16, 27]. We used a 3D spheroid system to confirm the results from the 2D culture system study, implying that we could expect a similar anti‐EHMT1 effect in a xenograft model derived from lung cancer cell lines. However, patient‐derived lung cancer organoids are more effective than 3D spheroid systems for estimating the clinical effect of EHMT1 expression in lung cancer patients because cancer organoids more accurately resemble cancer cells and the tumor environment [28, 29].

## 5 Conclusion

**Fig. 6:**
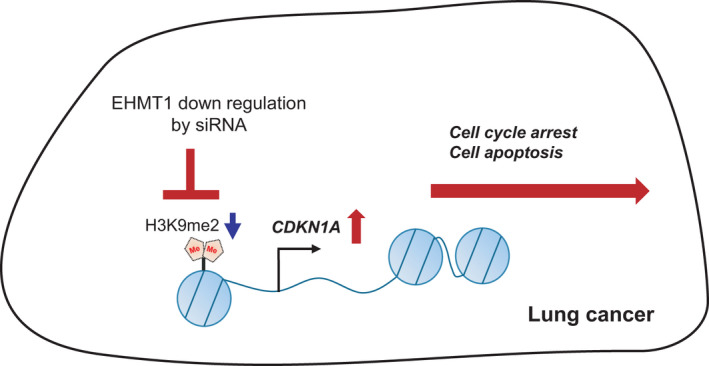
Schematic summarizing the role of EHMT1 in lung cancer. Overexpressed EHMT1 suppressed *CDKN1A* expression by epigenetic regulation. EHMT1‐specific inhibitors or siRNAs could induce the apoptosis and cell cycle arrest via upregulation of CDKN1A expression in lung cancer.

In this study, we found that EHMT1 is overexpressed in lung cancer and that EHMT1 modulates CDKN1A gene expression through the regulation of chromatin functions, subsequently promoting the proliferation of cancer cells by allowing them to evade apoptosis and cell cycle arrest (Fig. 6). Therefore, the development of EHMT1‐specific inhibitors is needed for lung cancer and other cancers.

## Conflict of interest

The authors declare no conflicts of interest.

## Author contributions

RH, MYS, DSK, and HSC conceived and designed the study. JL, KK, TYR, CRJ, JHL, and MSL developed the methodology. DSK, KP, MSL, and HSC analyzed and interpreted the data. CRJ, DSK, RH, MYS, and HSC wrote and reviewed the manuscript. DSK, RH, MYS, and HSC supervised the study.

### Peer Review

The peer review history for this article is available at https://publons.com/publon/10.1002/1878‐0261.13050.

## Supporting information


**Fig. S1**. Overexpression of EHMT1 in cancers.

**Table S1**. DEG list of RNA‐seq (red; upregulation and green; downregulation).

## Data Availability

The data that support the findings of this study are available from the corresponding author (chohs@kribb.re.kr) upon reasonable request.

## References

[1] Kouzarides T (2002) Histone methylation in transcriptional control. Curr Opin Genet Dev 12, 198–209. 11893494 10.1016/s0959-437x(02)00287-3

[2] Tachibana M , Matsumura Y , Fukuda M , Kimura H & Shinkai Y (2008) G9a/GLP complexes independently mediate H3K9 and DNA methylation to silence transcription. EMBO J 27, 2681–2690. 18818694 10.1038/emboj.2008.192 PMC2572175

[3] Tachibana M , Ueda J , Fukuda M , Takeda N , Ohta T , Iwanari H , Sakihama T , Kodama T , Hamakubo T & Shinkai Y (2005) Histone methyltransferases G9a and GLP form heteromeric complexes and are both crucial for methylation of euchromatin at H3–K9. Genes Dev 19, 815–826. 15774718 10.1101/gad.1284005 PMC1074319

[4] McGraw S , Vigneault C & Sirard MA (2007) Temporal expression of factors involved in chromatin remodeling and in gene regulation during early bovine in vitro embryo development. Reproduction 133, 597–608. 17379654 10.1530/REP-06-0251

[5] Shi Y , Sawada J , Sui G , el Affar B , Whetstine JR , Lan F , Ogawa H , Luke MP & Nakatani Y (2003) Coordinated histone modifications mediated by a CtBP co‐repressor complex. Nature 422, 735–738. 12700765 10.1038/nature01550

[6] Tachibana M , Sugimoto K , Nozaki M , Ueda J , Ohta T , Ohki M , Fukuda M , Takeda N , Niida H , Kato H *et al*. (2002) G9a histone methyltransferase plays a dominant role in euchromatic histone H3 lysine 9 methylation and is essential for early embryogenesis. Genes Dev 16, 1779–1791. 12130538 10.1101/gad.989402 PMC186403

[7] Kleefstra T , Brunner HG , Amiel J , Oudakker AR , Nillesen WM , Magee A , Genevieve D , Cormier‐Daire V , van Esch H , Fryns JP *et al*. (2006) Loss‐of‐function mutations in euchromatin histone methyl transferase 1 (EHMT1) cause the 9q34 subtelomeric deletion syndrome. Am J Hum Genet 79, 370–377. 16826528 10.1086/505693 PMC1559478

[8] Kleefstra T , Smidt M , Banning MJ , Oudakker AR , Van Esch H , de Brouwer AP , Nillesen W , Sistermans EA , Hamel BC , de Bruijn D *et al*. (2005) Disruption of the gene Euchromatin Histone Methyl Transferase1 (Eu‐HMTase1) is associated with the 9q34 subtelomeric deletion syndrome. J Med Genet 42, 299–306. 15805155 10.1136/jmg.2004.028464 PMC1736026

[9] Yatsenko SA , Brundage EK , Roney EK , Cheung SW , Chinault AC & Lupski JR (2009) Molecular mechanisms for subtelomeric rearrangements associated with the 9q34.3 microdeletion syndrome. Hum Mol Genet 18, 1924–1936. 19293338 10.1093/hmg/ddp114 PMC2678925

[10] Cebrian A , Pharoah PD , Ahmed S , Ropero S , Fraga MF , Smith PL , Conroy D , Luben R , Perkins B , Easton DF *et al*. (2006) Genetic variants in epigenetic genes and breast cancer risk. Carcinogenesis 27, 1661–1669. 16501248 10.1093/carcin/bgi375

[11] Lee H , Son YS , Lee MO , Ryu JW , Park K , Kwon O , Jung KB , Kim K , Ryu TY , Baek A *et al*. (2020) Low‐dose interleukin‐2 alleviates dextran sodium sulfate‐induced colitis in mice by recovering intestinal integrity and inhibiting AKT‐dependent pathways. Theranostics 10, 5048–5063. 32308767 10.7150/thno.41534 PMC7163458

[12] Abbas T & Dutta A (2009) p21 in cancer: intricate networks and multiple activities. Nat Rev Cancer 9, 400–414. 19440234 10.1038/nrc2657 PMC2722839

[13] Kleinsimon S , Longmuss E , Rolff J , Jager S , Eggert A , Delebinski C & Seifert G (2018) GADD45A and CDKN1A are involved in apoptosis and cell cycle modulatory effects of viscumTT with further inactivation of the STAT3 pathway. Sci Rep 8, 5750. 29636527 10.1038/s41598-018-24075-x PMC5893628

[14] Pitolli C , Wang Y , Candi E , Shi Y , Melino G & Amelio I (2019) p53‐mediated tumor suppression: DNA‐damage response and alternative mechanisms. Cancers (Basel) 11, 1983. 10.3390/cancers11121983 PMC6966539 31835405

[15] Lv D , Hu Z , Lu L , Lu H & Xu X (2017) Three‐dimensional cell culture: a powerful tool in tumor research and drug discovery. Oncol Lett 14, 6999–7010. 29344128 10.3892/ol.2017.7134 PMC5754907

[16] Nath S & Devi GR (2016) Three‐dimensional culture systems in cancer research: focus on tumor spheroid model. Pharmacol Ther 163, 94–108. 27063403 10.1016/j.pharmthera.2016.03.013 PMC4961208

[17] Ban HS , Han TS , Hur K & Cho HS (2019) Epigenetic alterations of Heat Shock Proteins (HSPs) in cancer. Int J Mol Sci 20, 4758. 10.3390/ijms20194758 PMC6801855 31557887

[18] Han TS , Ban HS , Hur K & Cho HS (2018) The epigenetic regulation of HCC metastasis. Int J Mol Sci 19, 3978. 10.3390/ijms19123978 PMC6321007 30544763

[19] Kim K , Ryu TY , Ryu JW , Han TS , Jung CR , Son MY , Kim DS & Cho HS (2020) RNA‐seq based transcriptome analysis of EHMT2 functions in breast cancer. Biochem Biophys Res Commun 524, 672–676. 32033749 10.1016/j.bbrc.2020.01.128

[20] Kim K , Son MY , Jung CR , Kim DS & Cho HS (2018) EHMT2 is a metastasis regulator in breast cancer. Biochem Biophys Res Commun 496, 758–762. 29337058 10.1016/j.bbrc.2018.01.074

[21] Kim SK , Kim K , Ryu JW , Ryu TY , Lim JH , Oh JH , Min JK , Jung CR , Hamamoto R , Son MY *et al*. (2019) The novel prognostic marker, EHMT2, is involved in cell proliferation via HSPD1 regulation in breast cancer. Int J Oncol 54, 65–76. 30365075 10.3892/ijo.2018.4608 PMC6254934

[22] Ryu TY , Kim K , Kim SK , Oh JH , Min JK , Jung CR , Son MY , Kim DS & Cho HS (2019) SETDB1 regulates SMAD7 expression for breast cancer metastasis. BMB Rep 52, 139–144. 30545440 10.5483/BMBRep.2019.52.2.235 PMC6443319

[23] Ryu TY , Kim K , Son MY , Min JK , Kim J , Han TS , Kim DS & Cho HS (2019) Downregulation of PRMT1, a histone arginine methyltransferase, by sodium propionate induces cell apoptosis in colon cancer. Oncol Rep 41, 1691–1699. 30569144 10.3892/or.2018.6938 PMC6365698

[24] Hamamoto R , Saloura V & Nakamura Y (2015) Critical roles of non‐histone protein lysine methylation in human tumorigenesis. Nat Rev Cancer 15, 110–124. 25614009 10.1038/nrc3884

[25] Guan X , Zhong X , Men W , Gong S , Zhang L & Han Y (2014) Analysis of EHMT1 expression and its correlations with clinical significance in esophageal squamous cell cancer. Mol Clin Oncol 2, 76–80. 24649311 10.3892/mco.2013.207 PMC3916188

[26] Ea CK , Hao S , Yeo KS & Baltimore D (2012) EHMT1 protein binds to nuclear factor‐kappaB p50 and represses gene expression. J Biol Chem 287, 31207–31217. 22801426 10.1074/jbc.M112.365601 PMC3438952

[27] Jung HR , Kang HM , Ryu JW , Kim DS , Noh KH , Kim ES , Lee HJ , Chung KS , Cho HS , Kim NS *et al*. (2017) Cell spheroids with enhanced aggressiveness to mimic human liver cancer in vitro and in vivo. Sci Rep 7, 10499. 28874716 10.1038/s41598-017-10828-7 PMC5585316

[28] Dijkstra KK , Monkhorst K , Schipper LJ , Hartemink KJ , Smit EF , Kaing S , de Groot R , Wolkers MC , Clevers H , Cuppen E *et al*. (2020) Challenges in establishing pure lung cancer organoids limit their utility for personalized medicine. Cell Rep 31, 107588. 32375033 10.1016/j.celrep.2020.107588

[29] Li Z , Qian Y , Li W , Liu L , Yu L , Liu X , Wu G , Wang Y , Luo W , Fang F *et al*. (2020) Human lung adenocarcinoma‐derived organoid models for drug screening. iScience 23, 101411. 32771979 10.1016/j.isci.2020.101411 PMC7415928

