# Supplementary material for: EHMT1 knockdown induces apoptosis and cell cycle arrest in lung cancer cells by increasing CDKN1A expression
Source: Mol Oncol. 2021 Jul 16;15(11):2989–3002. doi: 10.1002/1878-0261.13050 (PMC8564652; doi:10.1002/1878-0261.13050)
Supplement: Supplementary file 1 — Fig. S1. Overexpression of EHMT1 in cancers. [file MOL2-15-2989-s002.docx]

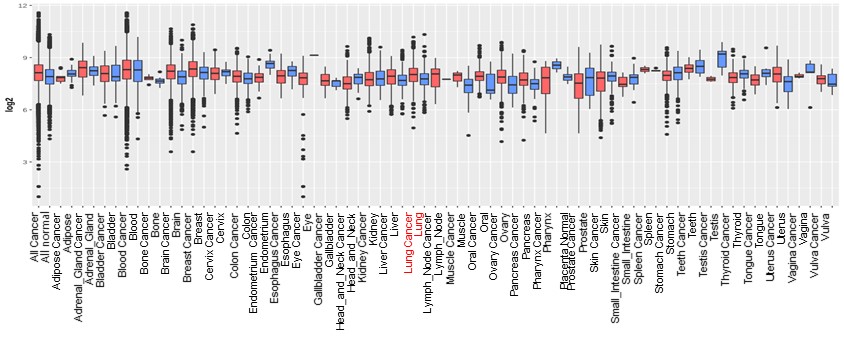


**Supplementary Fig. 1. Overexpression of EHMT1 in cancers.**

Tissue-wide expression profile of *EHMT1* in normal/cancer tissues using GENT2 analysis (http://gene2.appex.kr/gent2/)
