# Supplementary material for: EHMT1 knockdown induces apoptosis and cell cycle arrest in lung cancer cells by increasing CDKN1A expression
Source: Mol Oncol. 2021 Jul 16;15(11):2989–3002. doi: 10.1002/1878-0261.13050 (PMC8564652; doi:10.1002/1878-0261.13050)
Supplement: Supplementary file 2 — Table S1. DEG list of RNA‐seq (red; upregulation and green; downregulation). [file MOL2-15-2989-s001.docx]

**Supplementary table 1S. DEG list of RNA-seq** (Red : Up regulation, Green : Down regulation)

| ESG | Symbol | Fold (siEHMT1/siCont) | Log2 |
| --- | --- | --- | --- |
| ENSG00000262526 | AC120057.2 | 19.93414 | 4.317169461 |
| ENSG00000243709 | LEFTY1 | 9.9 | 3.307428525 |
| ENSG00000255526 | NEDD8-MDP1 | 7.81345 | 2.965959706 |
| ENSG00000257315 | ZBED6 | 7.396317906 | 2.886807235 |
| ENSG00000125954 | CHURC1-FNTB | 6.616321682 | 2.726029379 |
| ENSG00000105583 | WDR83OS | 6.178662748 | 2.627294628 |
| ENSG00000184524 | CEND1 | 6.039151041 | 2.594345756 |
| ENSG00000257065 | AL049844.1 | 5.291701276 | 2.403731622 |
| ENSG00000140044 | JDP2 | 5.174767722 | 2.371494106 |
| ENSG00000134590 | RTL8C | 5.002129193 | 2.322542319 |
| ENSG00000116731 | PRDM2 | 4.675941309 | 2.225256822 |
| ENSG00000188573 | FBLL1 | 4.325281216 | 2.112793935 |
| ENSG00000285304 | Z83844.3 | 4.3018815 | 2.104967785 |
| ENSG00000181704 | YIPF6 | 4.282058515 | 2.098304511 |
| ENSG00000261915 | AC026954.2 | 4.23557319 | 2.082557219 |
| ENSG00000248098 | BCKDHA | 4.227452761 | 2.079788633 |
| ENSG00000168374 | ARF4 | 4.227416524 | 2.079776267 |
| ENSG00000281039 | AC005154.5 | 4.20665 | 2.072671789 |
| ENSG00000067445 | TRO | 4.193674057 | 2.068214736 |
| ENSG00000175348 | TMEM9B | 4.05153456 | 2.018468447 |
| ENSG00000164104 | HMGB2 | 4.024660724 | 2.00886717 |
| ENSG00000267952 | AC008878.1 | 3.90635 | 1.965821217 |
| ENSG00000165806 | CASP7 | 3.867399165 | 1.951363677 |
| ENSG00000120738 | EGR1 | 3.796730346 | 1.924757539 |
| ENSG00000158164 | TMSB15A | 3.733324103 | 1.90046076 |
| ENSG00000181458 | TMEM45A | 3.680492697 | 1.879898909 |
| ENSG00000143320 | CRABP2 | 3.669320607 | 1.875512966 |
| ENSG00000148803 | FUOM | 3.564760057 | 1.833804973 |
| ENSG00000125898 | FAM110A | 3.557204043 | 1.83074373 |
| ENSG00000283782 | AC116366.3 | 3.54772 | 1.826892151 |
| ENSG00000187867 | PALM3 | 3.530885706 | 1.820030122 |
| ENSG00000197415 | VEPH1 | 3.507832295 | 1.810579776 |
| ENSG00000113558 | SKP1 | 3.487247599 | 1.802088801 |
| ENSG00000128564 | VGF | 3.470115261 | 1.794983583 |
| ENSG00000129946 | SHC2 | 3.43509099 | 1.780348314 |
| ENSG00000187837 | HIST1H1C | 3.417798362 | 1.773067285 |
| ENSG00000273802 | HIST1H2BG | 3.399356754 | 1.765261777 |
| ENSG00000089335 | ZNF302 | 3.330121414 | 1.735574778 |
| ENSG00000137965 | IFI44 | 3.322393372 | 1.732222899 |
| ENSG00000134548 | SPX | 3.317848891 | 1.730248181 |
| ENSG00000101255 | TRIB3 | 3.252432206 | 1.701518986 |
| ENSG00000139645 | ANKRD52 | 3.237152671 | 1.694725408 |
| ENSG00000215845 | TSTD1 | 3.202259329 | 1.679090147 |
| ENSG00000168872 | DDX19A | 3.190814264 | 1.673924633 |
| ENSG00000198003 | CCDC151 | 3.189983037 | 1.673548753 |
| ENSG00000143882 | ATP6V1C2 | 3.163540077 | 1.661539873 |
| ENSG00000103145 | HCFC1R1 | 3.159139503 | 1.659531646 |
| ENSG00000084070 | SMAP2 | 3.159034114 | 1.659483517 |
| ENSG00000155099 | PIP4P2 | 3.140392044 | 1.650944675 |
| ENSG00000278828 | HIST1H3H | 3.127884503 | 1.645187242 |
| ENSG00000158373 | HIST1H2BD | 3.112599451 | 1.638119934 |
| ENSG00000133134 | BEX2 | 3.112209941 | 1.637939384 |
| ENSG00000127870 | RNF6 | 3.085404613 | 1.625459694 |
| ENSG00000181031 | RPH3AL | 3.039270939 | 1.603725291 |
| ENSG00000175575 | PAAF1 | 3.027542437 | 1.598147182 |
| ENSG00000085831 | TTC39A | 3.026128125 | 1.597473072 |
| ENSG00000175567 | UCP2 | 3.015108695 | 1.592210012 |
| ENSG00000234511 | C5orf58 | 3.01231768 | 1.590873925 |
| ENSG00000130513 | GDF15 | 2.98582545 | 1.578129829 |
| ENSG00000127452 | FBXL12 | 2.981412572 | 1.575996031 |
| ENSG00000241489 | AC244197.3 | 2.975338188 | 1.57305366 |
| ENSG00000086548 | CEACAM6 | 2.974815855 | 1.572800366 |
| ENSG00000187634 | SAMD11 | 2.940285598 | 1.555956295 |
| ENSG00000259171 | AL163636.2 | 2.93841 | 1.555035711 |
| ENSG00000164620 | RELL2 | 2.932974036 | 1.5523643 |
| ENSG00000196361 | ELAVL3 | 2.93198537 | 1.551877905 |
| ENSG00000143622 | RIT1 | 2.919809759 | 1.545874373 |
| ENSG00000189367 | KIAA0408 | 2.891609736 | 1.531872853 |
| ENSG00000135406 | PRPH | 2.88975 | 1.530944687 |
| ENSG00000254772 | EEF1G | 2.867501746 | 1.519794364 |
| ENSG00000176383 | B3GNT4 | 2.859292587 | 1.515658256 |
| ENSG00000197965 | MPZL1 | 2.846486475 | 1.509182245 |
| ENSG00000175197 | DDIT3 | 2.843509061 | 1.507672401 |
| ENSG00000133169 | BEX1 | 2.838123847 | 1.504937546 |
| ENSG00000171345 | KRT19 | 2.829348889 | 1.500470088 |
| ENSG00000186480 | INSIG1 | 2.828040381 | 1.49980272 |
| ENSG00000149474 | KAT14 | 2.823083939 | 1.497272025 |
| ENSG00000119922 | IFIT2 | 2.81510534 | 1.493188909 |
| ENSG00000186462 | NAP1L2 | 2.814875909 | 1.493071324 |
| ENSG00000114698 | PLSCR4 | 2.811440155 | 1.491309337 |
| ENSG00000273003 | ARL2-SNX15 | 2.80922 | 1.490169611 |
| ENSG00000257529 | RPL36A-HNRNPH2 | 2.802744287 | 1.486840124 |
| ENSG00000105664 | COMP | 2.796529395 | 1.483637495 |
| ENSG00000123562 | MORF4L2 | 2.790581312 | 1.480565684 |
| ENSG00000173163 | COMMD1 | 2.789352985 | 1.479930515 |
| ENSG00000169169 | CPT1C | 2.77402 | 1.471978189 |
| ENSG00000184838 | PRR16 | 2.771564037 | 1.470700342 |
| ENSG00000124762 | CDKN1A | 2.768746459 | 1.469232948 |
| ENSG00000248167 | TRIM39-RPP21 | 2.76155 | 1.465478249 |
| ENSG00000205642 | VCX3B | 2.761469246 | 1.465436061 |
| ENSG00000159713 | TPPP3 | 2.76127095 | 1.46533246 |
| ENSG00000270276 | HIST2H4B | 2.758045611 | 1.463646315 |
| ENSG00000110921 | MVK | 2.755367182 | 1.462244586 |
| ENSG00000108433 | GOSR2 | 2.750403454 | 1.459643262 |
| ENSG00000140718 | FTO | 2.749598833 | 1.459221145 |
| ENSG00000090612 | ZNF268 | 2.748209672 | 1.458492077 |
| ENSG00000197956 | S100A6 | 2.743972713 | 1.456266135 |
| ENSG00000270882 | HIST2H4A | 2.742709865 | 1.455602016 |
| ENSG00000256061 | DNAAF4 | 2.736423461 | 1.452291504 |
| ENSG00000283706 | PRSS50 | 2.73093 | 1.449392335 |
| ENSG00000063322 | MED29 | 2.726884287 | 1.447253482 |
| ENSG00000198300 | PEG3 | 2.719279987 | 1.443224704 |
| ENSG00000007516 | BAIAP3 | 2.711393663 | 1.439034591 |
| ENSG00000105711 | SCN1B | 2.710274973 | 1.438439229 |
| ENSG00000135439 | AGAP2 | 2.706572741 | 1.436467162 |
| ENSG00000154553 | PDLIM3 | 2.702489639 | 1.434289087 |
| ENSG00000196787 | HIST1H2AG | 2.702176538 | 1.434121932 |
| ENSG00000077935 | SMC1B | 2.701415103 | 1.433715343 |
| ENSG00000064199 | SPA17 | 2.701353365 | 1.433682372 |
| ENSG00000168517 | HEXIM2 | 2.685905614 | 1.425408607 |
| ENSG00000088766 | CRLS1 | 2.68062147 | 1.422567511 |
| ENSG00000111725 | PRKAB1 | 2.680170434 | 1.422324745 |
| ENSG00000102109 | PCSK1N | 2.675995753 | 1.420075827 |
| ENSG00000134107 | BHLHE40 | 2.672197601 | 1.418026695 |
| ENSG00000189060 | H1F0 | 2.670227713 | 1.416962778 |
| ENSG00000114054 | PCCB | 2.665155724 | 1.414219832 |
| ENSG00000122694 | GLIPR2 | 2.659861925 | 1.411351357 |
| ENSG00000267740 | AC024592.3 | 2.659631497 | 1.411226368 |
| ENSG00000116678 | LEPR | 2.656402341 | 1.409473675 |
| ENSG00000178381 | ZFAND2A | 2.653893275 | 1.408110355 |
| ENSG00000084764 | MAPRE3 | 2.650266213 | 1.406137282 |
| ENSG00000184785 | SMIM10 | 2.648251328 | 1.405040045 |
| ENSG00000136490 | LIMD2 | 2.636112643 | 1.398412019 |
| ENSG00000007255 | TRAPPC6A | 2.632840943 | 1.396620367 |
| ENSG00000158006 | PAFAH2 | 2.625864153 | 1.392792281 |
| ENSG00000164284 | GRPEL2 | 2.623840308 | 1.391679917 |
| ENSG00000132692 | BCAN | 2.617794414 | 1.388351801 |
| ENSG00000184076 | UQCR10 | 2.614950424 | 1.386783595 |
| ENSG00000108821 | COL1A1 | 2.614395539 | 1.386477427 |
| ENSG00000128596 | CCDC136 | 2.611858016 | 1.385076472 |
| ENSG00000010310 | GIPR | 2.606821583 | 1.382291845 |
| ENSG00000100865 | CINP | 2.606412682 | 1.382065529 |
| ENSG00000241962 | AC079447.1 | 2.606254954 | 1.381978221 |
| ENSG00000188283 | ZNF383 | 2.603446106 | 1.380422542 |
| ENSG00000280571 | AC006059.2 | 2.601689455 | 1.379448768 |
| ENSG00000141682 | PMAIP1 | 2.599864891 | 1.378436651 |
| ENSG00000180425 | C11orf71 | 2.588834877 | 1.372302949 |
| ENSG00000228144 | AC078927.1 | 2.58536092 | 1.370365697 |
| ENSG00000121753 | ADGRB2 | 2.585017178 | 1.370173868 |
| ENSG00000159111 | MRPL10 | 2.578806347 | 1.366703439 |
| ENSG00000203778 | FAM229B | 2.574485626 | 1.364284215 |
| ENSG00000113597 | TRAPPC13 | 2.573040906 | 1.363474393 |
| ENSG00000115318 | LOXL3 | 2.558960542 | 1.355557902 |
| ENSG00000149557 | FEZ1 | 2.556309692 | 1.354062626 |
| ENSG00000150722 | PPP1R1C | 2.538888782 | 1.344197198 |
| ENSG00000008517 | IL32 | 2.536498087 | 1.342838073 |
| ENSG00000177989 | ODF3B | 2.534058435 | 1.341449793 |
| ENSG00000162174 | ASRGL1 | 2.529858036 | 1.33905643 |
| ENSG00000087842 | PIR | 2.52502349 | 1.336296809 |
| ENSG00000157036 | EXOG | 2.524245389 | 1.335852166 |
| ENSG00000130224 | LRCH2 | 2.523779152 | 1.33558567 |
| ENSG00000113742 | CPEB4 | 2.521354938 | 1.334199224 |
| ENSG00000107020 | PLGRKT | 2.517747707 | 1.332133724 |
| ENSG00000196754 | S100A2 | 2.500223908 | 1.322057301 |
| ENSG00000159200 | RCAN1 | 2.499631682 | 1.321715531 |
| ENSG00000104892 | KLC3 | 2.494043249 | 1.318486483 |
| ENSG00000154328 | NEIL2 | 2.491096296 | 1.316780791 |
| ENSG00000087076 | HSD17B14 | 2.487600622 | 1.314754883 |
| ENSG00000161642 | ZNF385A | 2.484431369 | 1.312915689 |
| ENSG00000188938 | FAM120AOS | 2.482057018 | 1.311536258 |
| ENSG00000005486 | RHBDD2 | 2.471915299 | 1.30562931 |
| ENSG00000095752 | IL11 | 2.469999328 | 1.304510649 |
| ENSG00000196150 | ZNF250 | 2.469101299 | 1.303986027 |
| ENSG00000131242 | RAB11FIP4 | 2.45997315 | 1.298642569 |
| ENSG00000180596 | HIST1H2BC | 2.453864443 | 1.295055554 |
| ENSG00000244187 | TMEM141 | 2.447896967 | 1.291542836 |
| ENSG00000105696 | AC003112.1 | 2.446695404 | 1.290834508 |
| ENSG00000238269 | PAGE2B | 2.444231076 | 1.289380683 |
| ENSG00000213625 | LEPROT | 2.442995435 | 1.288651167 |
| ENSG00000137713 | PPP2R1B | 2.441174451 | 1.287575397 |
| ENSG00000169894 | MUC3A | 2.440816932 | 1.287364093 |
| ENSG00000155961 | RAB39B | 2.438366661 | 1.285915082 |
| ENSG00000203812 | HIST2H2AA3 | 2.437350327 | 1.285313628 |
| ENSG00000103485 | QPRT | 2.433982511 | 1.283318802 |
| ENSG00000176410 | DNAJC30 | 2.433164064 | 1.282833602 |
| ENSG00000112972 | HMGCS1 | 2.432379023 | 1.282368053 |
| ENSG00000113068 | PFDN1 | 2.432289465 | 1.282314933 |
| ENSG00000198947 | DMD | 2.429922189 | 1.280910117 |
| ENSG00000143878 | RHOB | 2.426491307 | 1.278871692 |
| ENSG00000158985 | CDC42SE2 | 2.420538775 | 1.275328205 |
| ENSG00000133101 | CCNA1 | 2.418857025 | 1.274325496 |
| ENSG00000197409 | HIST1H3D | 2.418037186 | 1.273836431 |
| ENSG00000100478 | AP4S1 | 2.417725332 | 1.273650355 |
| ENSG00000145817 | YIPF5 | 2.416130598 | 1.272698438 |
| ENSG00000166847 | DCTN5 | 2.410481188 | 1.269321171 |
| ENSG00000158406 | HIST1H4H | 2.409909939 | 1.268979232 |
| ENSG00000154917 | RAB6B | 2.409766454 | 1.268893333 |
| ENSG00000111981 | ULBP1 | 2.408577173 | 1.26818115 |
| ENSG00000055208 | TAB2 | 2.408120762 | 1.267907742 |
| ENSG00000102879 | CORO1A | 2.403550393 | 1.265167052 |
| ENSG00000130707 | ASS1 | 2.40342679 | 1.265092859 |
| ENSG00000147316 | MCPH1 | 2.403406451 | 1.26508065 |
| ENSG00000115540 | MOB4 | 2.393107054 | 1.258884936 |
| ENSG00000134291 | TMEM106C | 2.38976447 | 1.256868436 |
| ENSG00000159917 | ZNF235 | 2.385889404 | 1.25452717 |
| ENSG00000278677 | HIST1H2AM | 2.384529274 | 1.253704495 |
| ENSG00000197355 | UAP1L1 | 2.383662178 | 1.253179786 |
| ENSG00000147642 | SYBU | 2.378987108 | 1.250347454 |
| ENSG00000076344 | RGS11 | 2.36622962 | 1.24259008 |
| ENSG00000162545 | CAMK2N1 | 2.365266854 | 1.242002961 |
| ENSG00000256591 | AP003108.2 | 2.362602328 | 1.240376816 |
| ENSG00000066923 | STAG3 | 2.362085699 | 1.240061308 |
| ENSG00000137504 | CREBZF | 2.361237443 | 1.239543124 |
| ENSG00000115935 | WIPF1 | 2.357520386 | 1.237270246 |
| ENSG00000151882 | CCL28 | 2.356165067 | 1.236440614 |
| ENSG00000243749 | TMEM35B | 2.351007583 | 1.233279192 |
| ENSG00000124208 | TMEM189-UBE2V1 | 2.35075482 | 1.233124076 |
| ENSG00000111206 | FOXM1 | 2.350475021 | 1.232952349 |
| ENSG00000137804 | NUSAP1 | 2.349004088 | 1.232049224 |
| ENSG00000125962 | ARMCX5 | 2.348457873 | 1.231713714 |
| ENSG00000140543 | DET1 | 2.346317925 | 1.230398511 |
| ENSG00000165272 | AQP3 | 2.345912708 | 1.230149331 |
| ENSG00000197363 | ZNF517 | 2.339158167 | 1.225989416 |
| ENSG00000163633 | C4orf36 | 2.33686 | 1.224571306 |
| ENSG00000108309 | RUNDC3A | 2.33484958 | 1.223329609 |
| ENSG00000175294 | CATSPER1 | 2.332337293 | 1.22177644 |
| ENSG00000119812 | FAM98A | 2.321160042 | 1.214845999 |
| ENSG00000076826 | CAMSAP3 | 2.320534924 | 1.21445741 |
| ENSG00000102144 | PGK1 | 2.31902768 | 1.21352004 |
| ENSG00000165416 | SUGT1 | 2.317166161 | 1.212361501 |
| ENSG00000168280 | KIF5C | 2.316874426 | 1.212179852 |
| ENSG00000127325 | BEST3 | 2.316168285 | 1.211740079 |
| ENSG00000130294 | KIF1A | 2.315481718 | 1.211312366 |
| ENSG00000271741 | AC114490.2 | 2.313533748 | 1.210098144 |
| ENSG00000020426 | MNAT1 | 2.312369546 | 1.209371977 |
| ENSG00000006432 | MAP3K9 | 2.305493865 | 1.205075826 |
| ENSG00000160181 | TFF2 | 2.304818057 | 1.204652868 |
| ENSG00000123353 | ORMDL2 | 2.303169505 | 1.203620592 |
| ENSG00000163866 | SMIM12 | 2.302242288 | 1.20303967 |
| ENSG00000154548 | SRSF12 | 2.298012203 | 1.200386459 |
| ENSG00000114200 | BCHE | 2.294333837 | 1.198075326 |
| ENSG00000286905 | AC108488.2 | 2.292373739 | 1.196842274 |
| ENSG00000255529 | POLR2M | 2.290919847 | 1.195926984 |
| ENSG00000146707 | POMZP3 | 2.290178435 | 1.195460008 |
| ENSG00000175175 | PPM1E | 2.289924586 | 1.195300087 |
| ENSG00000124749 | COL21A1 | 2.289484684 | 1.195022914 |
| ENSG00000149679 | CABLES2 | 2.28918183 | 1.194832061 |
| ENSG00000149016 | TUT1 | 2.288683698 | 1.194518093 |
| ENSG00000115884 | SDC1 | 2.286021646 | 1.192839064 |
| ENSG00000176909 | MAMSTR | 2.280407449 | 1.189291619 |
| ENSG00000112561 | TFEB | 2.278164525 | 1.18787194 |
| ENSG00000164967 | RPP25L | 2.277756007 | 1.187613214 |
| ENSG00000173267 | SNCG | 2.274791429 | 1.185734274 |
| ENSG00000071967 | CYBRD1 | 2.274751887 | 1.185709195 |
| ENSG00000285437 | POLR2J3 | 2.273013285 | 1.184606116 |
| ENSG00000105270 | CLIP3 | 2.271655577 | 1.183744113 |
| ENSG00000114503 | NCBP2 | 2.271515078 | 1.183654882 |
| ENSG00000100604 | CHGA | 2.270519565 | 1.183022468 |
| ENSG00000180573 | HIST1H2AC | 2.268161637 | 1.181523456 |
| ENSG00000187189 | TSPYL4 | 2.267198418 | 1.180910657 |
| ENSG00000124228 | DDX27 | 2.266531639 | 1.1804863 |
| ENSG00000103034 | NDRG4 | 2.256759995 | 1.174252997 |
| ENSG00000157693 | TMEM268 | 2.255517262 | 1.173458327 |
| ENSG00000267748 | AC011479.1 | 2.25368 | 1.172282682 |
| ENSG00000198832 | SELENOM | 2.249529338 | 1.169623182 |
| ENSG00000072682 | P4HA2 | 2.249282004 | 1.16946455 |
| ENSG00000197568 | HHLA3 | 2.248734653 | 1.169113436 |
| ENSG00000272196 | HIST2H2AA4 | 2.248151605 | 1.168739328 |
| ENSG00000100106 | TRIOBP | 2.247836374 | 1.168537022 |
| ENSG00000143727 | ACP1 | 2.246832461 | 1.167892551 |
| ENSG00000145781 | COMMD10 | 2.24663256 | 1.167764189 |
| ENSG00000127824 | TUBA4A | 2.246304071 | 1.167553231 |
| ENSG00000127324 | TSPAN8 | 2.245047727 | 1.166746115 |
| ENSG00000137707 | BTG4 | 2.242447778 | 1.165074388 |
| ENSG00000153879 | CEBPG | 2.241809271 | 1.164663541 |
| ENSG00000134769 | DTNA | 2.239412367 | 1.163120212 |
| ENSG00000118518 | RNF146 | 2.239131112 | 1.162939007 |
| ENSG00000140465 | CYP1A1 | 2.237823102 | 1.162095997 |
| ENSG00000263620 | AC129492.4 | 2.234170367 | 1.159739203 |
| ENSG00000151470 | C4orf33 | 2.233326025 | 1.159193874 |
| ENSG00000113140 | SPARC | 2.232273175 | 1.158513588 |
| ENSG00000088280 | ASAP3 | 2.231802245 | 1.158209199 |
| ENSG00000196793 | ZNF239 | 2.23103344 | 1.157712138 |
| ENSG00000156711 | MAPK13 | 2.227494239 | 1.155421701 |
| ENSG00000134193 | REG4 | 2.22639 | 1.154706334 |
| ENSG00000167100 | SAMD14 | 2.226238768 | 1.154608333 |
| ENSG00000118402 | ELOVL4 | 2.225033981 | 1.153827369 |
| ENSG00000173402 | DAG1 | 2.220356065 | 1.150791051 |
| ENSG00000133142 | TCEAL4 | 2.210908749 | 1.144639482 |
| ENSG00000243207 | PPAN-P2RY11 | 2.210552618 | 1.144407075 |
| ENSG00000131373 | HACL1 | 2.206967337 | 1.142065278 |
| ENSG00000088367 | EPB41L1 | 2.205580573 | 1.141158465 |
| ENSG00000164438 | TLX3 | 2.205053939 | 1.140813947 |
| ENSG00000142235 | LMTK3 | 2.203838691 | 1.14001863 |
| ENSG00000152700 | SAR1B | 2.19920789 | 1.136983988 |
| ENSG00000111254 | AKAP3 | 2.192680219 | 1.132695425 |
| ENSG00000262481 | TMEM256-PLSCR3 | 2.191754079 | 1.132085933 |
| ENSG00000196747 | HIST1H2AI | 2.190347983 | 1.13116009 |
| ENSG00000166002 | SMCO4 | 2.188821037 | 1.130154001 |
| ENSG00000187908 | DMBT1 | 2.188594717 | 1.130004821 |
| ENSG00000182247 | UBE2E2 | 2.188091663 | 1.129673176 |
| ENSG00000104888 | SLC17A7 | 2.184796325 | 1.127498792 |
| ENSG00000233041 | PHGR1 | 2.183996381 | 1.126970466 |
| ENSG00000237289 | CKMT1B | 2.183521285 | 1.126656595 |
| ENSG00000119729 | RHOQ | 2.180589017 | 1.124717886 |
| ENSG00000174370 | C11orf45 | 2.179726713 | 1.124147266 |
| ENSG00000169629 | RGPD8 | 2.177803801 | 1.122873987 |
| ENSG00000137628 | DDX60 | 2.177228311 | 1.122492701 |
| ENSG00000148019 | CEP78 | 2.176534094 | 1.122032619 |
| ENSG00000048392 | RRM2B | 2.17594996 | 1.121645379 |
| ENSG00000078177 | N4BP2 | 2.175651201 | 1.121447283 |
| ENSG00000119844 | AFTPH | 2.174221701 | 1.120499057 |
| ENSG00000134287 | ARF3 | 2.173802457 | 1.120220842 |
| ENSG00000176049 | JAKMIP2 | 2.17304286 | 1.11971663 |
| ENSG00000250479 | CHCHD10 | 2.172178544 | 1.119142691 |
| ENSG00000197183 | NOL4L | 2.171592062 | 1.118753115 |
| ENSG00000132000 | PODNL1 | 2.171540267 | 1.118718705 |
| ENSG00000165259 | HDX | 2.170492993 | 1.118022765 |
| ENSG00000099977 | DDT | 2.168737421 | 1.11685539 |
| ENSG00000082458 | DLG3 | 2.167409789 | 1.115971948 |
| ENSG00000129355 | CDKN2D | 2.164053134 | 1.113735922 |
| ENSG00000248712 | CCDC153 | 2.163864518 | 1.113610173 |
| ENSG00000100934 | SEC23A | 2.163755643 | 1.113537582 |
| ENSG00000130517 | PGPEP1 | 2.162223714 | 1.112515799 |
| ENSG00000204264 | PSMB8 | 2.156649267 | 1.108791572 |
| ENSG00000154040 | CABYR | 2.156438534 | 1.108650595 |
| ENSG00000184060 | ADAP2 | 2.156009286 | 1.108363392 |
| ENSG00000148341 | SH3GLB2 | 2.15586675 | 1.108268011 |
| ENSG00000115828 | QPCT | 2.155427683 | 1.10797416 |
| ENSG00000132581 | SDF2 | 2.1550699 | 1.107734664 |
| ENSG00000167861 | HID1 | 2.152010337 | 1.105685008 |
| ENSG00000176884 | GRIN1 | 2.150992821 | 1.10500271 |
| ENSG00000145022 | TCTA | 2.150943227 | 1.104969446 |
| ENSG00000157423 | HYDIN | 2.150346633 | 1.104569239 |
| ENSG00000182379 | NXPH4 | 2.148430412 | 1.103283049 |
| ENSG00000180758 | GPR157 | 2.147785965 | 1.10285023 |
| ENSG00000213523 | SRA1 | 2.145803111 | 1.101517707 |
| ENSG00000166922 | SCG5 | 2.142452375 | 1.099263135 |
| ENSG00000158856 | DMTN | 2.14210399 | 1.099028518 |
| ENSG00000172922 | RNASEH2C | 2.141376665 | 1.098538586 |
| ENSG00000167797 | CDK2AP2 | 2.141280046 | 1.09847349 |
| ENSG00000102409 | BEX4 | 2.137180238 | 1.095708582 |
| ENSG00000104267 | CA2 | 2.135630161 | 1.094661829 |
| ENSG00000136404 | TM6SF1 | 2.135363021 | 1.094481355 |
| ENSG00000133739 | LRRCC1 | 2.133373939 | 1.093136864 |
| ENSG00000269096 | CT45A3 | 2.129249956 | 1.09034532 |
| ENSG00000136059 | VILL | 2.129165994 | 1.09028843 |
| ENSG00000165215 | CLDN3 | 2.128974048 | 1.090158364 |
| ENSG00000077380 | DYNC1I2 | 2.127348692 | 1.089056524 |
| ENSG00000164176 | EDIL3 | 2.126882075 | 1.088740045 |
| ENSG00000128610 | FEZF1 | 2.126218782 | 1.088290054 |
| ENSG00000272398 | CD24 | 2.125114367 | 1.087540485 |
| ENSG00000167460 | TPM4 | 2.122971619 | 1.086085085 |
| ENSG00000101294 | HM13 | 2.122396768 | 1.085694384 |
| ENSG00000165943 | MOAP1 | 2.122243019 | 1.08558987 |
| ENSG00000204396 | VWA7 | 2.120959974 | 1.084717395 |
| ENSG00000114127 | XRN1 | 2.120187143 | 1.084191613 |
| ENSG00000181218 | HIST3H2A | 2.119898786 | 1.083995385 |
| ENSG00000180964 | TCEAL8 | 2.119645803 | 1.083823208 |
| ENSG00000140941 | MAP1LC3B | 2.11691227 | 1.081961482 |
| ENSG00000105409 | ATP1A3 | 2.116118985 | 1.081420749 |
| ENSG00000137285 | TUBB2B | 2.114119837 | 1.080057157 |
| ENSG00000144674 | GOLGA4 | 2.112981743 | 1.079280302 |
| ENSG00000175155 | YPEL2 | 2.112905199 | 1.079228038 |
| ENSG00000158769 | F11R | 2.112270779 | 1.07879479 |
| ENSG00000213654 | GPSM3 | 2.108767851 | 1.076400281 |
| ENSG00000176401 | EID2B | 2.108040828 | 1.075902809 |
| ENSG00000173436 | MICOS10 | 2.105576329 | 1.074215175 |
| ENSG00000187608 | ISG15 | 2.105472534 | 1.074144055 |
| ENSG00000162772 | ATF3 | 2.105207988 | 1.073962774 |
| ENSG00000128165 | ADM2 | 2.104473077 | 1.073459053 |
| ENSG00000162522 | KIAA1522 | 2.104199209 | 1.073271294 |
| ENSG00000137814 | HAUS2 | 2.103975989 | 1.07311824 |
| ENSG00000166681 | BEX3 | 2.103403095 | 1.072725354 |
| ENSG00000166780 | BMERB1 | 2.102961644 | 1.072422537 |
| ENSG00000154358 | OBSCN | 2.101237473 | 1.071239218 |
| ENSG00000109738 | GLRB | 2.101006997 | 1.071080966 |
| ENSG00000141179 | PCTP | 2.100928403 | 1.071026998 |
| ENSG00000197279 | ZNF165 | 2.100168218 | 1.070504889 |
| ENSG00000111142 | METAP2 | 2.097246205 | 1.068496236 |
| ENSG00000111540 | AC034102.1 | 2.094460325 | 1.066578556 |
| ENSG00000262660 | AC139530.3 | 2.09304 | 1.065599883 |
| ENSG00000171224 | FAM241B | 2.092852205 | 1.065470434 |
| ENSG00000186088 | GSAP | 2.092138933 | 1.06497866 |
| ENSG00000196072 | BLOC1S2 | 2.09097218 | 1.064173867 |
| ENSG00000071189 | SNX13 | 2.090694355 | 1.063982165 |
| ENSG00000105427 | CNFN | 2.089984961 | 1.063492561 |
| ENSG00000179674 | ARL14 | 2.089462891 | 1.063132136 |
| ENSG00000154589 | LY96 | 2.087359295 | 1.061678951 |
| ENSG00000205457 | TP53TG3C | 2.08582437 | 1.060617686 |
| ENSG00000203950 | RTL8A | 2.084901495 | 1.059979222 |
| ENSG00000090013 | BLVRB | 2.081586853 | 1.057683755 |
| ENSG00000134758 | RNF138 | 2.078222366 | 1.055350028 |
| ENSG00000151012 | SLC7A11 | 2.077192856 | 1.054635169 |
| ENSG00000130479 | MAP1S | 2.075674258 | 1.053580055 |
| ENSG00000108448 | TRIM16L | 2.075456548 | 1.053428728 |
| ENSG00000185437 | SH3BGR | 2.075437791 | 1.053415689 |
| ENSG00000072609 | CHFR | 2.072888735 | 1.05164268 |
| ENSG00000188610 | FAM72B | 2.070893656 | 1.05025347 |
| ENSG00000162241 | SLC25A45 | 2.070305421 | 1.049843617 |
| ENSG00000163596 | ICA1L | 2.069819709 | 1.049505108 |
| ENSG00000138669 | PRKG2 | 2.068488989 | 1.048577278 |
| ENSG00000118971 | CCND2 | 2.068222862 | 1.048391652 |
| ENSG00000219545 | UMAD1 | 2.068120307 | 1.048320112 |
| ENSG00000153975 | ZUP1 | 2.06727746 | 1.047732033 |
| ENSG00000198001 | IRAK4 | 2.065361268 | 1.046394156 |
| ENSG00000197959 | DNM3 | 2.064580395 | 1.045848599 |
| ENSG00000135924 | DNAJB2 | 2.06448153 | 1.045779511 |
| ENSG00000163738 | MTHFD2L | 2.063919266 | 1.045386538 |
| ENSG00000196544 | BORCS6 | 2.06347075 | 1.045072988 |
| ENSG00000060762 | MPC1 | 2.05651303 | 1.040200213 |
| ENSG00000223572 | CKMT1A | 2.055579528 | 1.039545189 |
| ENSG00000178053 | MLF1 | 2.05405807 | 1.038476969 |
| ENSG00000140678 | ITGAX | 2.053842757 | 1.038325732 |
| ENSG00000100292 | HMOX1 | 2.051563049 | 1.036723492 |
| ENSG00000132510 | KDM6B | 2.051523094 | 1.036695395 |
| ENSG00000006128 | TAC1 | 2.05148 | 1.036665089 |
| ENSG00000243279 | PRAF2 | 2.050446316 | 1.035937972 |
| ENSG00000188620 | HMX3 | 2.049915673 | 1.035564563 |
| ENSG00000139428 | MMAB | 2.049595144 | 1.035338963 |
| ENSG00000186314 | PRELID2 | 2.049446913 | 1.03523462 |
| ENSG00000153048 | CARHSP1 | 2.047125577 | 1.033599605 |
| ENSG00000176087 | SLC35A4 | 2.04688054 | 1.033426906 |
| ENSG00000184281 | TSSC4 | 2.046752105 | 1.033336379 |
| ENSG00000101400 | SNTA1 | 2.046258646 | 1.032988512 |
| ENSG00000163521 | GLB1L | 2.043118524 | 1.030772899 |
| ENSG00000206538 | VGLL3 | 2.042926557 | 1.03063734 |
| ENSG00000163848 | ZNF148 | 2.042094102 | 1.030049349 |
| ENSG00000127561 | SYNGR3 | 2.04126356 | 1.02946247 |
| ENSG00000121931 | LRIF1 | 2.037076479 | 1.026500145 |
| ENSG00000188419 | CHM | 2.036524887 | 1.026109445 |
| ENSG00000105499 | PLA2G4C | 2.032834862 | 1.023493022 |
| ENSG00000130208 | APOC1 | 2.032380904 | 1.023170814 |
| ENSG00000070540 | WIPI1 | 2.032298518 | 1.02311233 |
| ENSG00000168528 | SERINC2 | 2.030270877 | 1.021672224 |
| ENSG00000197045 | GMFB | 2.02918395 | 1.020899655 |
| ENSG00000159479 | MED8 | 2.028889614 | 1.020690375 |
| ENSG00000268193 | AC002985.1 | 2.028727663 | 1.020575211 |
| ENSG00000136156 | ITM2B | 2.028472472 | 1.020393724 |
| ENSG00000134202 | GSTM3 | 2.022109482 | 1.01586111 |
| ENSG00000134594 | RAB33A | 2.02166 | 1.015540387 |
| ENSG00000152464 | RPP38 | 2.021393983 | 1.01535054 |
| ENSG00000182208 | MOB2 | 2.016608858 | 1.011931286 |
| ENSG00000213928 | IRF9 | 2.016478857 | 1.011838279 |
| ENSG00000068078 | FGFR3 | 2.01617735 | 1.011622549 |
| ENSG00000164463 | CREBRF | 2.015688554 | 1.011272744 |
| ENSG00000078804 | TP53INP2 | 2.013999914 | 1.010063622 |
| ENSG00000131389 | SLC6A6 | 2.013514604 | 1.009715936 |
| ENSG00000135631 | RAB11FIP5 | 2.011990812 | 1.008623717 |
| ENSG00000104381 | GDAP1 | 2.011901677 | 1.008559801 |
| ENSG00000064012 | CASP8 | 2.010935243 | 1.007866624 |
| ENSG00000166797 | CIAO2A | 2.010189479 | 1.007331495 |
| ENSG00000063854 | HAGH | 2.010138031 | 1.007294571 |
| ENSG00000135245 | HILPDA | 2.009396676 | 1.006762395 |
| ENSG00000163531 | NFASC | 2.008991285 | 1.006471306 |
| ENSG00000105419 | MEIS3 | 2.008375906 | 1.006029323 |
| ENSG00000183054 | RGPD6 | 2.007731476 | 1.005566329 |
| ENSG00000108370 | RGS9 | 2.007272548 | 1.00523652 |
| ENSG00000143653 | SCCPDH | 2.005267957 | 1.003795032 |
| ENSG00000117477 | CCDC181 | 2.004539995 | 1.003271203 |
| ENSG00000015568 | RGPD5 | 2.004382547 | 1.00315788 |
| ENSG00000117758 | STX12 | 2.003778507 | 1.002723045 |
| ENSG00000182472 | CAPN12 | 2.003603735 | 1.002597206 |
| ENSG00000198551 | ZNF627 | 2.003524943 | 1.002540471 |
| ENSG00000125124 | BBS2 | 2.002386752 | 1.001720651 |
| ENSG00000163798 | SLC4A1AP | 2.001984351 | 1.001430697 |
| ENSG00000180304 | OAZ2 | 2.001895705 | 1.001366815 |
| ENSG00000116032 | GRIN3B | 2.001682376 | 1.001213067 |
| ENSG00000151332 | MBIP | 2.000918721 | 1.000662565 |
| ENSG00000156515 | HK1 | 2.000173082 | 1.000124847 |
| ENSG00000132849 | PATJ | 1.998536755 | 0.998944105 |
| ENSG00000165060 | FXN | 1.998231258 | 0.998723558 |
| ENSG00000105926 | MPP6 | 1.998158371 | 0.998670934 |
| ENSG00000114405 | C3orf14 | 1.997951162 | 0.998521318 |
| ENSG00000171219 | CDC42BPG | 1.997659034 | 0.998310361 |
| ENSG00000106772 | PRUNE2 | 1.996834302 | 0.997714622 |
| ENSG00000249242 | TMEM150C | 1.995125678 | 0.996479628 |
| ENSG00000196507 | TCEAL3 | 1.994662489 | 0.996144653 |
| ENSG00000103528 | SYT17 | 1.9930607 | 0.994985649 |
| ENSG00000132522 | GPS2 | 1.991589015 | 0.993919963 |
| ENSG00000124380 | SNRNP27 | 1.990544327 | 0.993162999 |
| ENSG00000127526 | SLC35E1 | 1.990529036 | 0.993151916 |
| ENSG00000197580 | BCO2 | 1.990515051 | 0.99314178 |
| ENSG00000161082 | CELF5 | 1.98841002 | 0.991615279 |
| ENSG00000078018 | MAP2 | 1.985481266 | 0.989488748 |
| ENSG00000138138 | ATAD1 | 1.98475372 | 0.98896 |
| ENSG00000184160 | ADRA2C | 1.983105934 | 0.987761745 |
| ENSG00000176396 | EID2 | 1.981527593 | 0.986613057 |
| ENSG00000136147 | PHF11 | 1.979342964 | 0.985021612 |
| ENSG00000092096 | SLC22A17 | 1.978420487 | 0.984349084 |
| ENSG00000138413 | IDH1 | 1.978354028 | 0.984300621 |
| ENSG00000101363 | MANBAL | 1.977994555 | 0.984038455 |
| ENSG00000157601 | MX1 | 1.977690523 | 0.983816685 |
| ENSG00000151150 | ANK3 | 1.976530641 | 0.98297032 |
| ENSG00000137135 | ARHGEF39 | 1.975665649 | 0.982338813 |
| ENSG00000133678 | TMEM254 | 1.975597009 | 0.98228869 |
| ENSG00000167306 | MYO5B | 1.975119636 | 0.981940042 |
| ENSG00000143452 | HORMAD1 | 1.973310398 | 0.980617907 |
| ENSG00000160446 | ZDHHC12 | 1.97292902 | 0.980339053 |
| ENSG00000214413 | BBIP1 | 1.972632875 | 0.980122483 |
| ENSG00000176845 | METRNL | 1.971712783 | 0.979449411 |
| ENSG00000116698 | SMG7 | 1.971063275 | 0.978974091 |
| ENSG00000205302 | SNX2 | 1.970462589 | 0.978534359 |
| ENSG00000153790 | C7orf31 | 1.969145813 | 0.977569945 |
| ENSG00000153485 | TMEM251 | 1.968621827 | 0.977185995 |
| ENSG00000162702 | ZNF281 | 1.968391953 | 0.977017524 |
| ENSG00000211445 | GPX3 | 1.966731787 | 0.975800224 |
| ENSG00000104549 | SQLE | 1.966049106 | 0.975299357 |
| ENSG00000143369 | ECM1 | 1.965953876 | 0.975229474 |
| ENSG00000255339 | AL133352.1 | 1.965594451 | 0.97496569 |
| ENSG00000155729 | KCTD18 | 1.964695573 | 0.974305786 |
| ENSG00000135451 | TROAP | 1.964686747 | 0.974299305 |
| ENSG00000163888 | CAMK2N2 | 1.963905087 | 0.973725208 |
| ENSG00000105668 | UPK1A | 1.962824583 | 0.972931245 |
| ENSG00000119139 | TJP2 | 1.961865348 | 0.972226026 |
| ENSG00000198420 | TCAF1 | 1.959841958 | 0.97073732 |
| ENSG00000115266 | APC2 | 1.958914888 | 0.970054716 |
| ENSG00000153531 | ADPRHL1 | 1.957884148 | 0.9692954 |
| ENSG00000138867 | GUCD1 | 1.957034403 | 0.968669117 |
| ENSG00000104976 | SNAPC2 | 1.956136846 | 0.968007301 |
| ENSG00000061656 | SPAG4 | 1.955715036 | 0.967696173 |
| ENSG00000078070 | MCCC1 | 1.954505334 | 0.966803522 |
| ENSG00000129675 | ARHGEF6 | 1.953604768 | 0.966138627 |
| ENSG00000173930 | SLCO4C1 | 1.950725069 | 0.964010462 |
| ENSG00000170264 | FAM161A | 1.950552255 | 0.963882649 |
| ENSG00000128185 | DGCR6L | 1.949988905 | 0.963465916 |
| ENSG00000151466 | SCLT1 | 1.94979491 | 0.963322381 |
| ENSG00000127995 | CASD1 | 1.949369006 | 0.963007211 |
| ENSG00000103723 | AP3B2 | 1.949172309 | 0.962861632 |
| ENSG00000198814 | GK | 1.948101811 | 0.962069077 |
| ENSG00000177000 | MTHFR | 1.945805952 | 0.960367843 |
| ENSG00000059758 | CDK17 | 1.945451444 | 0.960104973 |
| ENSG00000169926 | KLF13 | 1.943422965 | 0.958599922 |
| ENSG00000134874 | DZIP1 | 1.94212092 | 0.957633029 |
| ENSG00000277203 | F8A1 | 1.941964843 | 0.957517083 |
| ENSG00000185187 | SIGIRR | 1.941862543 | 0.957441082 |
| ENSG00000140992 | PDPK1 | 1.941039266 | 0.956829303 |
| ENSG00000119782 | FKBP1B | 1.940739174 | 0.95660624 |
| ENSG00000167996 | FTH1 | 1.940606925 | 0.956507926 |
| ENSG00000177875 | CCDC184 | 1.94056285 | 0.95647516 |
| ENSG00000160161 | CILP2 | 1.94017533 | 0.956187032 |
| ENSG00000101247 | NDUFAF5 | 1.939817071 | 0.955920609 |
| ENSG00000149792 | MRPL49 | 1.939441416 | 0.955641198 |
| ENSG00000237651 | C2orf74 | 1.938602492 | 0.955017011 |
| ENSG00000108813 | DLX4 | 1.934757478 | 0.952152736 |
| ENSG00000168038 | ULK4 | 1.934744621 | 0.952143149 |
| ENSG00000178761 | FAM219B | 1.934716706 | 0.952122333 |
| ENSG00000136603 | SKIL | 1.934315328 | 0.951822999 |
| ENSG00000008735 | MAPK8IP2 | 1.931909526 | 0.950027532 |
| ENSG00000188428 | BLOC1S5 | 1.931615971 | 0.949808297 |
| ENSG00000067840 | PDZD4 | 1.931516047 | 0.949733663 |
| ENSG00000185130 | HIST1H2BL | 1.931201061 | 0.949498374 |
| ENSG00000108771 | DHX58 | 1.930673416 | 0.949104146 |
| ENSG00000185787 | MORF4L1 | 1.930211807 | 0.948759167 |
| ENSG00000169955 | ZNF747 | 1.929905873 | 0.948530485 |
| ENSG00000112305 | SMAP1 | 1.928819254 | 0.947717957 |
| ENSG00000162520 | SYNC | 1.928723211 | 0.947646118 |
| ENSG00000251503 | CENPS-CORT | 1.926412107 | 0.945916364 |
| ENSG00000042317 | AL049834.1 | 1.925578136 | 0.945291666 |
| ENSG00000155629 | PIK3AP1 | 1.925179132 | 0.94499269 |
| ENSG00000106633 | GCK | 1.923873851 | 0.944014204 |
| ENSG00000185519 | FAM131C | 1.923806764 | 0.943963896 |
| ENSG00000277150 | F8A3 | 1.923070801 | 0.943411879 |
| ENSG00000129595 | EPB41L4A | 1.9222125 | 0.942767834 |
| ENSG00000135899 | SP110 | 1.920785884 | 0.941696706 |
| ENSG00000163961 | RNF168 | 1.920629829 | 0.941579489 |
| ENSG00000100554 | ATP6V1D | 1.920531631 | 0.941505725 |
| ENSG00000138738 | PRDM5 | 1.919678422 | 0.940864656 |
| ENSG00000158553 | POM121L2 | 1.919659058 | 0.940850103 |
| ENSG00000196748 | CLPSL2 | 1.91927 | 0.940557682 |
| ENSG00000132563 | REEP2 | 1.918449326 | 0.939940658 |
| ENSG00000254999 | BRK1 | 1.918400868 | 0.939904217 |
| ENSG00000135931 | ARMC9 | 1.918364831 | 0.939877115 |
| ENSG00000128311 | TST | 1.918069462 | 0.939654968 |
| ENSG00000076248 | UNG | 1.91768959 | 0.939369215 |
| ENSG00000087085 | ACHE | 1.91720966 | 0.939008115 |
| ENSG00000165996 | HACD1 | 1.916813667 | 0.9387101 |
| ENSG00000137821 | LRRC49 | 1.916076396 | 0.938155084 |
| ENSG00000128000 | ZNF780B | 1.915670881 | 0.937849722 |
| ENSG00000115194 | SLC30A3 | 1.915475665 | 0.937702697 |
| ENSG00000197903 | HIST1H2BK | 1.915423153 | 0.937663145 |
| ENSG00000169903 | TM4SF4 | 1.915045391 | 0.937378588 |
| ENSG00000265818 | EEF1E1-BLOC1S5 | 1.91485 | 0.937231383 |
| ENSG00000168214 | RBPJ | 1.914283819 | 0.936804745 |
| ENSG00000081087 | OSTM1 | 1.914095596 | 0.936662884 |
| ENSG00000104093 | DMXL2 | 1.914073617 | 0.936646318 |
| ENSG00000100593 | ISM2 | 1.91396 | 0.936560679 |
| ENSG00000225950 | NTF4 | 1.913300749 | 0.936063667 |
| ENSG00000196967 | ZNF585A | 1.913141036 | 0.935943233 |
| ENSG00000160460 | SPTBN4 | 1.912820155 | 0.935701237 |
| ENSG00000112379 | ARFGEF3 | 1.912352411 | 0.93534841 |
| ENSG00000092758 | COL9A3 | 1.911096926 | 0.93440095 |
| ENSG00000139970 | RTN1 | 1.911041288 | 0.934358948 |
| ENSG00000135604 | STX11 | 1.910503992 | 0.933953272 |
| ENSG00000164211 | STARD4 | 1.910323064 | 0.93381664 |
| ENSG00000126432 | PRDX5 | 1.909975939 | 0.933554464 |
| ENSG00000166200 | COPS2 | 1.909769456 | 0.933398489 |
| ENSG00000111912 | NCOA7 | 1.909606756 | 0.933275575 |
| ENSG00000155330 | C16orf87 | 1.909294423 | 0.933039591 |
| ENSG00000105613 | MAST1 | 1.90919471 | 0.932964244 |
| ENSG00000178449 | COX14 | 1.909031373 | 0.932840812 |
| ENSG00000214309 | MBLAC1 | 1.907588686 | 0.931750131 |
| ENSG00000110871 | COQ5 | 1.907241933 | 0.931487861 |
| ENSG00000160282 | FTCD | 1.906794244 | 0.931149175 |
| ENSG00000171130 | ATP6V0E2 | 1.90667007 | 0.931055221 |
| ENSG00000264058 | AC073508.2 | 1.906228618 | 0.930721155 |
| ENSG00000109944 | JHY | 1.906149239 | 0.930661077 |
| ENSG00000183628 | DGCR6 | 1.905365326 | 0.93006764 |
| ENSG00000178927 | CYBC1 | 1.904601842 | 0.929489433 |
| ENSG00000161955 | TNFSF13 | 1.903936297 | 0.928985209 |
| ENSG00000172667 | ZMAT3 | 1.902898418 | 0.928198549 |
| ENSG00000138764 | CCNG2 | 1.902650433 | 0.928010525 |
| ENSG00000123472 | ATPAF1 | 1.902533048 | 0.927921514 |
| ENSG00000082701 | GSK3B | 1.902267645 | 0.927720244 |
| ENSG00000109046 | WSB1 | 1.901238021 | 0.926939158 |
| ENSG00000214595 | EML6 | 1.899957794 | 0.925967371 |
| ENSG00000065882 | TBC1D1 | 1.899950003 | 0.925961455 |
| ENSG00000140682 | TGFB1I1 | 1.899853695 | 0.925888323 |
| ENSG00000091164 | TXNL1 | 1.899320424 | 0.925483315 |
| ENSG00000136449 | MYCBPAP | 1.898903298 | 0.925166438 |
| ENSG00000166831 | RBPMS2 | 1.897399685 | 0.924023613 |
| ENSG00000213390 | ARHGAP19 | 1.897382562 | 0.924010593 |
| ENSG00000160233 | LRRC3 | 1.897303602 | 0.923950554 |
| ENSG00000181773 | GPR3 | 1.897282415 | 0.923934444 |
| ENSG00000100445 | SDR39U1 | 1.896523552 | 0.923357288 |
| ENSG00000183248 | PRR36 | 1.896485145 | 0.923328071 |
| ENSG00000260001 | TGFBR3L | 1.895878751 | 0.922866701 |
| ENSG00000006283 | CACNA1G | 1.894934766 | 0.922148184 |
| ENSG00000179292 | TMEM151A | 1.894894582 | 0.922117589 |
| ENSG00000120868 | APAF1 | 1.894574549 | 0.921873909 |
| ENSG00000156587 | UBE2L6 | 1.893479297 | 0.921039647 |
| ENSG00000145743 | FBXL17 | 1.893310905 | 0.920911339 |
| ENSG00000253276 | CCDC71L | 1.893290212 | 0.920895571 |
| ENSG00000132205 | EMILIN2 | 1.892448172 | 0.92025379 |
| ENSG00000164093 | PITX2 | 1.892428714 | 0.920238957 |
| ENSG00000120899 | PTK2B | 1.892410801 | 0.9202253 |
| ENSG00000150281 | CTF1 | 1.891440356 | 0.919485284 |
| ENSG00000109089 | CDR2L | 1.891049278 | 0.919186958 |
| ENSG00000083750 | RRAGB | 1.890942416 | 0.91910543 |
| ENSG00000086061 | DNAJA1 | 1.890519586 | 0.918782796 |
| ENSG00000105792 | CFAP69 | 1.890336979 | 0.918643438 |
| ENSG00000134480 | CCNH | 1.890152536 | 0.918502665 |
| ENSG00000187601 | MAGEH1 | 1.889537902 | 0.918033458 |
| ENSG00000178537 | SLC25A20 | 1.889106058 | 0.9177037 |
| ENSG00000250317 | SMIM20 | 1.888746838 | 0.91742934 |
| ENSG00000147251 | DOCK11 | 1.888646158 | 0.917352435 |
| ENSG00000137267 | TUBB2A | 1.888197768 | 0.917009879 |
| ENSG00000078900 | TP73 | 1.887513524 | 0.916486981 |
| ENSG00000156639 | ZFAND3 | 1.887417059 | 0.916413248 |
| ENSG00000133256 | PDE6B | 1.887248203 | 0.916284172 |
| ENSG00000109832 | DDX25 | 1.886981805 | 0.916080512 |
| ENSG00000104823 | ECH1 | 1.886674521 | 0.915845558 |
| ENSG00000107537 | PHYH | 1.886536809 | 0.915740249 |
| ENSG00000088356 | PDRG1 | 1.885939101 | 0.91528309 |
| ENSG00000237172 | B3GNT9 | 1.885845276 | 0.915211315 |
| ENSG00000197124 | ZNF682 | 1.883925462 | 0.913741885 |
| ENSG00000109576 | AADAT | 1.883243424 | 0.913219492 |
| ENSG00000151725 | CENPU | 1.88310327 | 0.91311212 |
| ENSG00000283761 | AC118553.2 | 1.882745157 | 0.912837734 |
| ENSG00000196376 | SLC35F1 | 1.882354117 | 0.91253806 |
| ENSG00000173898 | SPTBN2 | 1.881460228 | 0.911852792 |
| ENSG00000248751 | AC004997.1 | 1.881004093 | 0.911502988 |
| ENSG00000122735 | DNAI1 | 1.879362984 | 0.910243738 |
| ENSG00000161249 | DMKN | 1.878914604 | 0.909899498 |
| ENSG00000052802 | MSMO1 | 1.878371275 | 0.909482251 |
| ENSG00000137261 | KIAA0319 | 1.878035976 | 0.9092247 |
| ENSG00000123496 | IL13RA2 | 1.877540911 | 0.908844344 |
| ENSG00000111087 | GLI1 | 1.876773448 | 0.908254508 |
| ENSG00000173531 | MST1 | 1.876511746 | 0.908053321 |
| ENSG00000204301 | NOTCH4 | 1.875538127 | 0.907304591 |
| ENSG00000166833 | NAV2 | 1.875315373 | 0.907133235 |
| ENSG00000137500 | CCDC90B | 1.874914069 | 0.906824475 |
| ENSG00000169599 | NFU1 | 1.873782293 | 0.905953342 |
| ENSG00000276368 | HIST1H2AJ | 1.87281304 | 0.905206884 |
| ENSG00000204822 | MRPL53 | 1.872588935 | 0.905034238 |
| ENSG00000182628 | SKA2 | 1.871627683 | 0.904293473 |
| ENSG00000054179 | ENTPD2 | 1.871082346 | 0.903873053 |
| ENSG00000246705 | H2AFJ | 1.86968966 | 0.902798824 |
| ENSG00000137880 | GCHFR | 1.868661324 | 0.902005119 |
| ENSG00000011454 | RABGAP1 | 1.868308271 | 0.90173252 |
| ENSG00000180773 | SLC36A4 | 1.86830314 | 0.901728557 |
| ENSG00000120324 | PCDHB10 | 1.867584204 | 0.901173291 |
| ENSG00000072062 | PRKACA | 1.867500821 | 0.901108877 |
| ENSG00000176105 | YES1 | 1.867239252 | 0.900906794 |
| ENSG00000131711 | MAP1B | 1.867087228 | 0.90078933 |
| ENSG00000214575 | CPEB1 | 1.867004205 | 0.900725177 |
| ENSG00000073969 | NSF | 1.866962584 | 0.900693015 |
| ENSG00000204356 | NELFE | 1.865802194 | 0.899796045 |
| ENSG00000105290 | APLP1 | 1.864530502 | 0.898812399 |
| ENSG00000175416 | CLTB | 1.864183093 | 0.898543563 |
| ENSG00000166313 | APBB1 | 1.863001154 | 0.897628568 |
| ENSG00000001629 | ANKIB1 | 1.8627359 | 0.897423142 |
| ENSG00000152102 | FAM168B | 1.862641452 | 0.897349991 |
| ENSG00000143742 | SRP9 | 1.862176174 | 0.896989567 |
| ENSG00000254995 | STX16-NPEPL1 | 1.860509645 | 0.895697869 |
| ENSG00000168564 | CDKN2AIP | 1.860399358 | 0.895612347 |
| ENSG00000151743 | AMN1 | 1.859403741 | 0.894840064 |
| ENSG00000273983 | HIST1H3G | 1.858479463 | 0.894122746 |
| ENSG00000163683 | SMIM14 | 1.858255298 | 0.893948721 |
| ENSG00000135596 | MICAL1 | 1.858114973 | 0.893839773 |
| ENSG00000152527 | PLEKHH2 | 1.858028223 | 0.893772416 |
| ENSG00000112977 | DAP | 1.855630582 | 0.891909528 |
| ENSG00000158301 | GPRASP2 | 1.85555839 | 0.8918534 |
| ENSG00000258643 | BCL2L2-PABPN1 | 1.854981075 | 0.891404468 |
| ENSG00000133789 | SWAP70 | 1.854284872 | 0.890862901 |
| ENSG00000138650 | PCDH10 | 1.854279371 | 0.890858621 |
| ENSG00000135127 | BICDL1 | 1.853240361 | 0.890050008 |
| ENSG00000129480 | DTD2 | 1.85256504 | 0.889524194 |
| ENSG00000169249 | ZRSR2 | 1.852261561 | 0.889287839 |
| ENSG00000184979 | USP18 | 1.85193236 | 0.889031407 |
| ENSG00000182621 | PLCB1 | 1.851735883 | 0.888878338 |
| ENSG00000125743 | SNRPD2 | 1.851115544 | 0.888394949 |
| ENSG00000133131 | MORC4 | 1.851003493 | 0.888307618 |
| ENSG00000179083 | FAM133A | 1.850864024 | 0.888198909 |
| ENSG00000161860 | SYCE2 | 1.850793662 | 0.888144063 |
| ENSG00000127952 | STYXL1 | 1.850763653 | 0.888120671 |
| ENSG00000164418 | GRIK2 | 1.85020172 | 0.887682571 |
| ENSG00000203667 | COX20 | 1.849920574 | 0.88746333 |
| ENSG00000165434 | PGM2L1 | 1.849157571 | 0.886868165 |
| ENSG00000220205 | VAMP2 | 1.84875859 | 0.886556851 |
| ENSG00000232859 | LYRM9 | 1.84833752 | 0.886228227 |
| ENSG00000174099 | MSRB3 | 1.847671707 | 0.885708442 |
| ENSG00000123636 | BAZ2B | 1.847177696 | 0.885322659 |
| ENSG00000073803 | MAP3K13 | 1.846956314 | 0.885149742 |
| ENSG00000184702 | SEPT5 | 1.846576441 | 0.884852986 |
| ENSG00000231887 | PRH1 | 1.846522254 | 0.88481065 |
| ENSG00000273542 | HIST1H4K | 1.846369734 | 0.884691481 |
| ENSG00000213859 | KCTD11 | 1.845751504 | 0.884208334 |
| ENSG00000141854 | MISP3 | 1.845339796 | 0.883886495 |
| ENSG00000160097 | FNDC5 | 1.845280823 | 0.883840389 |
| ENSG00000196368 | NUDT11 | 1.845254855 | 0.883820086 |
| ENSG00000241127 | YAE1 | 1.8450043 | 0.883624179 |
| ENSG00000074370 | ATP2A3 | 1.844533515 | 0.883256003 |
| ENSG00000082014 | SMARCD3 | 1.84437431 | 0.883131476 |
| ENSG00000198585 | NUDT16 | 1.843067804 | 0.882109147 |
| ENSG00000108946 | PRKAR1A | 1.842369571 | 0.881562489 |
| ENSG00000147065 | MSN | 1.842325799 | 0.881528211 |
| ENSG00000164294 | GPX8 | 1.842260847 | 0.881477348 |
| ENSG00000151414 | NEK7 | 1.841621072 | 0.880976246 |
| ENSG00000240694 | PNMA2 | 1.841173787 | 0.880625808 |
| ENSG00000274791 | F8A2 | 1.841079905 | 0.880552242 |
| ENSG00000145020 | AMT | 1.840050758 | 0.879745564 |
| ENSG00000078081 | LAMP3 | 1.840035132 | 0.879733312 |
| ENSG00000124635 | HIST1H2BJ | 1.838813584 | 0.878775229 |
| ENSG00000117020 | AKT3 | 1.83868642 | 0.878675455 |
| ENSG00000130303 | BST2 | 1.838357076 | 0.878417018 |
| ENSG00000143443 | C1orf56 | 1.836901048 | 0.877273912 |
| ENSG00000198353 | HOXC4 | 1.836741856 | 0.877148878 |
| ENSG00000164220 | F2RL2 | 1.836114153 | 0.876655756 |
| ENSG00000183569 | SERHL2 | 1.836067926 | 0.876619432 |
| ENSG00000173421 | CCDC36 | 1.835778709 | 0.876392162 |
| ENSG00000277363 | SRCIN1 | 1.834956518 | 0.875745877 |
| ENSG00000060140 | STYK1 | 1.834416028 | 0.875320866 |
| ENSG00000164506 | STXBP5 | 1.83440547 | 0.875312562 |
| ENSG00000171368 | TPPP | 1.834279712 | 0.875213655 |
| ENSG00000233822 | HIST1H2BN | 1.833623901 | 0.874697755 |
| ENSG00000286132 | AC022415.2 | 1.833391489 | 0.874514881 |
| ENSG00000203797 | DDO | 1.832747109 | 0.87400773 |
| ENSG00000213145 | CRIP1 | 1.832169014 | 0.873552595 |
| ENSG00000132326 | PER2 | 1.832006373 | 0.873424522 |
| ENSG00000119698 | PPP4R4 | 1.830806017 | 0.872478938 |
| ENSG00000278224 | PRICKLE4 | 1.830262455 | 0.872050542 |
| ENSG00000100867 | DHRS2 | 1.829039505 | 0.871086236 |
| ENSG00000197021 | CXorf40B | 1.828300442 | 0.870503166 |
| ENSG00000141376 | BCAS3 | 1.827016124 | 0.869489366 |
| ENSG00000166275 | BORCS7 | 1.826975423 | 0.869457226 |
| ENSG00000157873 | TNFRSF14 | 1.826263021 | 0.868894559 |
| ENSG00000185880 | TRIM69 | 1.826061345 | 0.868735232 |
| ENSG00000105619 | TFPT | 1.824881997 | 0.867803177 |
| ENSG00000060718 | COL11A1 | 1.824476396 | 0.867482486 |
| ENSG00000140057 | AK7 | 1.823320714 | 0.866568347 |
| ENSG00000164749 | HNF4G | 1.823256637 | 0.866517646 |
| ENSG00000105784 | RUNDC3B | 1.821048908 | 0.864769669 |
| ENSG00000155329 | ZCCHC10 | 1.820098811 | 0.864016775 |
| ENSG00000171016 | PYGO1 | 1.81971394 | 0.863711676 |
| ENSG00000184203 | PPP1R2 | 1.819477605 | 0.863524294 |
| ENSG00000147144 | CCDC120 | 1.819175661 | 0.863284858 |
| ENSG00000126247 | CAPNS1 | 1.818646979 | 0.862865526 |
| ENSG00000069424 | KCNAB2 | 1.81830174 | 0.862591629 |
| ENSG00000128609 | NDUFA5 | 1.818217949 | 0.862525146 |
| ENSG00000135423 | GLS2 | 1.818113969 | 0.862442638 |
| ENSG00000131094 | C1QL1 | 1.817911784 | 0.862282193 |
| ENSG00000137710 | RDX | 1.81764255 | 0.862068513 |
| ENSG00000161921 | CXCL16 | 1.816866605 | 0.8614525 |
| ENSG00000155868 | MED7 | 1.816199416 | 0.860922617 |
| ENSG00000163754 | GYG1 | 1.815834052 | 0.860632362 |
| ENSG00000147041 | SYTL5 | 1.815709161 | 0.860533132 |
| ENSG00000124120 | TTPAL | 1.815581379 | 0.860431597 |
| ENSG00000184898 | RBM43 | 1.814766274 | 0.859783754 |
| ENSG00000214194 | SMIM30 | 1.813497919 | 0.85877509 |
| ENSG00000175279 | CENPS | 1.813387482 | 0.858687231 |
| ENSG00000117481 | NSUN4 | 1.812893321 | 0.858294033 |
| ENSG00000242259 | C22orf39 | 1.812138851 | 0.857693503 |
| ENSG00000116663 | FBXO6 | 1.811789831 | 0.857415611 |
| ENSG00000167508 | MVD | 1.811449854 | 0.857144869 |
| ENSG00000005436 | GCFC2 | 1.810887551 | 0.856696964 |
| ENSG00000109189 | USP46 | 1.810570323 | 0.856444213 |
| ENSG00000122299 | ZC3H7A | 1.81009551 | 0.856065823 |
| ENSG00000168734 | PKIG | 1.810042694 | 0.856023727 |
| ENSG00000154065 | ANKRD29 | 1.809758177 | 0.855796935 |
| ENSG00000162641 | AKNAD1 | 1.808557614 | 0.854839558 |
| ENSG00000156500 | FAM122C | 1.807640193 | 0.854107541 |
| ENSG00000107611 | CUBN | 1.807515339 | 0.85400789 |
| ENSG00000091428 | RAPGEF4 | 1.80681077 | 0.853445419 |
| ENSG00000089163 | SIRT4 | 1.806730171 | 0.853381061 |
| ENSG00000177383 | MAGEF1 | 1.806542158 | 0.853230923 |
| ENSG00000132963 | POMP | 1.806473315 | 0.853175944 |
| ENSG00000104231 | ZFAND1 | 1.80562797 | 0.852500672 |
| ENSG00000240065 | PSMB9 | 1.804986545 | 0.851988083 |
| ENSG00000170689 | HOXB9 | 1.804677432 | 0.851740993 |
| ENSG00000135828 | RNASEL | 1.803949492 | 0.851158945 |
| ENSG00000124257 | NEURL2 | 1.803922813 | 0.851137609 |
| ENSG00000134996 | OSTF1 | 1.803600167 | 0.850879549 |
| ENSG00000136238 | RAC1 | 1.802239474 | 0.849790723 |
| ENSG00000138071 | ACTR2 | 1.801759141 | 0.849406164 |
| ENSG00000100075 | SLC25A1 | 1.800631628 | 0.848503066 |
| ENSG00000092871 | RFFL | 1.800622966 | 0.848496125 |
| ENSG00000188629 | ZNF177 | 1.800413074 | 0.848327946 |
| ENSG00000155111 | CDK19 | 1.7993165 | 0.847448979 |
| ENSG00000102081 | FMR1 | 1.799181273 | 0.84734055 |
| ENSG00000010404 | IDS | 1.799038474 | 0.84722604 |
| ENSG00000171291 | ZNF439 | 1.798328143 | 0.846656295 |
| ENSG00000165905 | LARGE2 | 1.798126787 | 0.846494749 |
| ENSG00000214050 | FBXO16 | 1.79703249 | 0.845616493 |
| ENSG00000163743 | RCHY1 | 1.796891141 | 0.84550301 |
| ENSG00000166145 | SPINT1 | 1.796847129 | 0.845467673 |
| ENSG00000151640 | DPYSL4 | 1.79679 | 0.845421804 |
| ENSG00000186260 | MRTFB | 1.796681667 | 0.845334818 |
| ENSG00000047249 | ATP6V1H | 1.796194544 | 0.844943615 |
| ENSG00000286185 | AC242842.3 | 1.795767935 | 0.844600924 |
| ENSG00000168676 | KCTD19 | 1.795074748 | 0.84404392 |
| ENSG00000065609 | SNAP91 | 1.794844739 | 0.843859051 |
| ENSG00000109062 | SLC9A3R1 | 1.793996662 | 0.843177206 |
| ENSG00000188760 | TMEM198 | 1.793898677 | 0.843098406 |
| ENSG00000273611 | ZNHIT3 | 1.793583278 | 0.842844732 |
| ENSG00000022840 | RNF10 | 1.793513532 | 0.842788631 |
| ENSG00000004848 | ARX | 1.793505419 | 0.842782104 |
| ENSG00000118004 | COLEC11 | 1.793275696 | 0.842597303 |
| ENSG00000157077 | ZFYVE9 | 1.793200957 | 0.842537175 |
| ENSG00000196605 | ZNF846 | 1.793135553 | 0.842484554 |
| ENSG00000159761 | C16orf86 | 1.792968531 | 0.842350167 |
| ENSG00000109586 | GALNT7 | 1.792762867 | 0.842184672 |
| ENSG00000183597 | TANGO2 | 1.792540154 | 0.842005436 |
| ENSG00000155876 | RRAGA | 1.791684826 | 0.841316877 |
| ENSG00000166173 | LARP6 | 1.791176452 | 0.840907467 |
| ENSG00000010219 | DYRK4 | 1.791050403 | 0.840805938 |
| ENSG00000151553 | FAM160B1 | 1.79072515 | 0.840543922 |
| ENSG00000163156 | SCNM1 | 1.79049059 | 0.840354936 |
| ENSG00000189159 | JPT1 | 1.789902727 | 0.839881186 |
| ENSG00000122729 | ACO1 | 1.789803273 | 0.839801021 |
| ENSG00000120262 | CCDC170 | 1.789573566 | 0.839615851 |
| ENSG00000176108 | CHMP6 | 1.789363624 | 0.839446593 |
| ENSG00000124107 | SLPI | 1.789322108 | 0.83941312 |
| ENSG00000181381 | DDX60L | 1.788956439 | 0.839118258 |
| ENSG00000171160 | MORN4 | 1.788511986 | 0.838759787 |
| ENSG00000091129 | NRCAM | 1.788463259 | 0.838720481 |
| ENSG00000196132 | MYT1 | 1.788322399 | 0.838606849 |
| ENSG00000078687 | TNRC6C | 1.788041915 | 0.838380556 |
| ENSG00000181652 | ATG9B | 1.787307276 | 0.837787686 |
| ENSG00000266967 | AARSD1 | 1.787126525 | 0.837641778 |
| ENSG00000115145 | STAM2 | 1.787019989 | 0.837555772 |
| ENSG00000104722 | NEFM | 1.786985046 | 0.837527561 |
| ENSG00000277075 | HIST1H2AE | 1.786680334 | 0.837281536 |
| ENSG00000177483 | RBM44 | 1.786327762 | 0.836996816 |
| ENSG00000173210 | ABLIM3 | 1.785467228 | 0.836301654 |
| ENSG00000154640 | BTG3 | 1.785428345 | 0.836270235 |
| ENSG00000068079 | IFI35 | 1.785230591 | 0.836110434 |
| ENSG00000163565 | IFI16 | 1.785161959 | 0.836054969 |
| ENSG00000175556 | LONRF3 | 1.784925818 | 0.835864116 |
| ENSG00000122644 | ARL4A | 1.784907679 | 0.835849456 |
| ENSG00000141510 | TP53 | 1.784487428 | 0.835509737 |
| ENSG00000164867 | NOS3 | 1.784112004 | 0.835206189 |
| ENSG00000198429 | ZNF69 | 1.783818439 | 0.834968782 |
| ENSG00000006704 | GTF2IRD1 | 1.783125182 | 0.834407989 |
| ENSG00000166262 | FAM227B | 1.782752011 | 0.834106032 |
| ENSG00000114107 | CEP70 | 1.782548219 | 0.833941103 |
| ENSG00000160058 | BSDC1 | 1.781894151 | 0.83341164 |
| ENSG00000119431 | HDHD3 | 1.781113491 | 0.832779447 |
| ENSG00000189180 | ZNF33A | 1.780752193 | 0.832486767 |
| ENSG00000185522 | LMNTD2 | 1.780681149 | 0.832429209 |
| ENSG00000197380 | DACT3 | 1.78057502 | 0.832343221 |
| ENSG00000284308 | C2orf81 | 1.780534847 | 0.832310671 |
| ENSG00000197841 | ZNF181 | 1.77999114 | 0.83187006 |
| ENSG00000076258 | FMO4 | 1.778988131 | 0.831056886 |
| ENSG00000073050 | XRCC1 | 1.778806252 | 0.83090938 |
| ENSG00000249709 | ZNF564 | 1.778195149 | 0.830413662 |
| ENSG00000198836 | OPA1 | 1.778023637 | 0.830274504 |
| ENSG00000203666 | EFCAB2 | 1.77798721 | 0.830244946 |
| ENSG00000181061 | HIGD1A | 1.777227756 | 0.829628578 |
| ENSG00000149485 | FADS1 | 1.777191946 | 0.829599508 |
| ENSG00000188452 | CERKL | 1.776240484 | 0.82882692 |
| ENSG00000100167 | SEPT3 | 1.775982304 | 0.828617207 |
| ENSG00000130203 | APOE | 1.775572174 | 0.828284004 |
| ENSG00000099785 | MARCH2 | 1.775542127 | 0.82825959 |
| ENSG00000168938 | PPIC | 1.775436111 | 0.828173446 |
| ENSG00000070669 | ASNS | 1.775161094 | 0.827949954 |
| ENSG00000170396 | ZNF804A | 1.773696465 | 0.82675914 |
| ENSG00000099860 | GADD45B | 1.772715834 | 0.825961291 |
| ENSG00000115204 | MPV17 | 1.77135344 | 0.824852103 |
| ENSG00000124092 | CTCFL | 1.771124033 | 0.824665248 |
| ENSG00000123552 | USP45 | 1.770993523 | 0.824558936 |
| ENSG00000135108 | FBXO21 | 1.770962448 | 0.824533621 |
| ENSG00000136371 | MTHFS | 1.770649614 | 0.824278752 |
| ENSG00000257103 | LSM14A | 1.770350262 | 0.824034824 |
| ENSG00000067606 | PRKCZ | 1.770143138 | 0.823866025 |
| ENSG00000140961 | OSGIN1 | 1.769099487 | 0.823015181 |
| ENSG00000188322 | SBK1 | 1.769049127 | 0.822974113 |
| ENSG00000120509 | PDZD11 | 1.769042635 | 0.822968818 |
| ENSG00000089737 | DDX24 | 1.768540172 | 0.82255899 |
| ENSG00000221821 | C6orf226 | 1.768535512 | 0.822555189 |
| ENSG00000258102 | MAP1LC3B2 | 1.768477798 | 0.822508107 |
| ENSG00000124486 | USP9X | 1.768059571 | 0.822166884 |
| ENSG00000221994 | ZNF630 | 1.767611824 | 0.821801487 |
| ENSG00000124333 | VAMP7 | 1.7675538 | 0.821754128 |
| ENSG00000164332 | UBLCP1 | 1.767333294 | 0.821574137 |
| ENSG00000198513 | ATL1 | 1.767087442 | 0.821373431 |
| ENSG00000167315 | ACAA2 | 1.766357759 | 0.820777577 |
| ENSG00000214357 | NEURL1B | 1.766027701 | 0.820507973 |
| ENSG00000177409 | SAMD9L | 1.765991113 | 0.820478083 |
| ENSG00000112667 | DNPH1 | 1.765699061 | 0.820239477 |
| ENSG00000275493 | AL627230.1 | 1.765367705 | 0.819968711 |
| ENSG00000104904 | OAZ1 | 1.765365294 | 0.81996674 |
| ENSG00000117697 | NSL1 | 1.765072296 | 0.819727276 |
| ENSG00000173258 | ZNF483 | 1.765035625 | 0.819697303 |
| ENSG00000138386 | NAB1 | 1.764818248 | 0.819519614 |
| ENSG00000128918 | ALDH1A2 | 1.764808727 | 0.81951183 |
| ENSG00000175224 | ATG13 | 1.764150006 | 0.818973238 |
| ENSG00000235863 | B3GALT4 | 1.763838803 | 0.818718719 |
| ENSG00000186352 | ANKRD37 | 1.763809224 | 0.818694526 |
| ENSG00000131019 | ULBP3 | 1.763752926 | 0.818648476 |
| ENSG00000169019 | COMMD8 | 1.763720427 | 0.818621892 |
| ENSG00000103353 | UBFD1 | 1.763147628 | 0.818153276 |
| ENSG00000197943 | PLCG2 | 1.762959033 | 0.81799895 |
| ENSG00000204116 | CHIC1 | 1.76185454 | 0.81709482 |
| ENSG00000165533 | TTC8 | 1.761493396 | 0.816799066 |
| ENSG00000141068 | KSR1 | 1.761278726 | 0.816623237 |
| ENSG00000076944 | STXBP2 | 1.760909038 | 0.816320387 |
| ENSG00000167785 | ZNF558 | 1.760892387 | 0.816306745 |
| ENSG00000167554 | ZNF610 | 1.760593521 | 0.816061864 |
| ENSG00000123358 | NR4A1 | 1.760433622 | 0.815930831 |
| ENSG00000144891 | AGTR1 | 1.760363069 | 0.81587301 |
| ENSG00000102021 | LUZP4 | 1.760124403 | 0.8156774 |
| ENSG00000184545 | DUSP8 | 1.760121515 | 0.815675033 |
| ENSG00000127125 | PPCS | 1.759324273 | 0.81502142 |
| ENSG00000153147 | SMARCA5 | 1.758884113 | 0.814660431 |
| ENSG00000170962 | PDGFD | 1.758832413 | 0.814618025 |
| ENSG00000129514 | FOXA1 | 1.758017517 | 0.813949446 |
| ENSG00000151092 | NGLY1 | 1.757536717 | 0.813554829 |
| ENSG00000131370 | SH3BP5 | 1.757373011 | 0.813420443 |
| ENSG00000128581 | IFT22 | 1.757007387 | 0.813120257 |
| ENSG00000169564 | PCBP1 | 1.756422805 | 0.812640172 |
| ENSG00000180185 | FAHD1 | 1.755984061 | 0.812279749 |
| ENSG00000179918 | SEPHS2 | 1.755888548 | 0.812201275 |
| ENSG00000105991 | HOXA1 | 1.755793984 | 0.812123577 |
| ENSG00000113734 | BNIP1 | 1.755791668 | 0.812121673 |
| ENSG00000167380 | ZNF226 | 1.755776658 | 0.812109339 |
| ENSG00000104953 | TLE6 | 1.755457015 | 0.81184667 |
| ENSG00000161956 | SENP3 | 1.755302043 | 0.811719303 |
| ENSG00000188906 | LRRK2 | 1.754842493 | 0.811341546 |
| ENSG00000172239 | PAIP1 | 1.754491859 | 0.811053253 |
| ENSG00000130876 | SLC7A10 | 1.754236025 | 0.810842869 |
| ENSG00000070367 | EXOC5 | 1.753989003 | 0.810639703 |
| ENSG00000185842 | DNAH14 | 1.753863682 | 0.810536619 |
| ENSG00000204308 | RNF5 | 1.753315711 | 0.810085799 |
| ENSG00000196954 | CASP4 | 1.752966113 | 0.809798107 |
| ENSG00000281991 | TMEM265 | 1.7524883 | 0.809404813 |
| ENSG00000083097 | DOP1A | 1.752457901 | 0.809379787 |
| ENSG00000261787 | TCF24 | 1.752405275 | 0.809336463 |
| ENSG00000254996 | ANKHD1-EIF4EBP3 | 1.752238 | 0.809198744 |
| ENSG00000175602 | CCDC85B | 1.751987676 | 0.808992626 |
| ENSG00000148219 | ASTN2 | 1.750961907 | 0.808147698 |
| ENSG00000101670 | LIPG | 1.749555458 | 0.806988396 |
| ENSG00000125841 | NRSN2 | 1.749483154 | 0.806928773 |
| ENSG00000179761 | PIPOX | 1.74942 | 0.806876692 |
| ENSG00000125967 | NECAB3 | 1.747969991 | 0.805680417 |
| ENSG00000100526 | CDKN3 | 1.747911112 | 0.80563182 |
| ENSG00000154118 | JPH3 | 1.747892668 | 0.805616597 |
| ENSG00000170584 | NUDCD2 | 1.747810497 | 0.805548772 |
| ENSG00000115514 | TXNDC9 | 1.747597999 | 0.805373359 |
| ENSG00000163170 | BOLA3 | 1.747170265 | 0.805020209 |
| ENSG00000182400 | TRAPPC6B | 1.746037141 | 0.804084247 |
| ENSG00000253485 | PCDHGA5 | 1.745957291 | 0.804018269 |
| ENSG00000180530 | NRIP1 | 1.745505793 | 0.803645145 |
| ENSG00000122705 | CLTA | 1.745262604 | 0.803444131 |
| ENSG00000137076 | TLN1 | 1.745098904 | 0.803308804 |
| ENSG00000173535 | TNFRSF10C | 1.743909428 | 0.802325114 |
| ENSG00000167555 | ZNF528 | 1.743525607 | 0.802007553 |
| ENSG00000224109 | CENPVL3 | 1.743342176 | 0.801855763 |
| ENSG00000067596 | DHX8 | 1.743266974 | 0.801793529 |
| ENSG00000084453 | SLCO1A2 | 1.743148292 | 0.801695306 |
| ENSG00000212864 | RNF208 | 1.743133817 | 0.801683327 |
| ENSG00000215472 | RPL17-C18orf32 | 1.742882873 | 0.801475619 |
| ENSG00000154359 | LONRF1 | 1.742745291 | 0.801361729 |
| ENSG00000187164 | SHTN1 | 1.742690884 | 0.801316689 |
| ENSG00000179222 | MAGED1 | 1.741377422 | 0.800228923 |
| ENSG00000099899 | TRMT2A | 1.740902709 | 0.79983558 |
| ENSG00000136888 | ATP6V1G1 | 1.740705912 | 0.799672484 |
| ENSG00000111247 | RAD51AP1 | 1.739946435 | 0.799042892 |
| ENSG00000010810 | FYN | 1.739806587 | 0.798926932 |
| ENSG00000003096 | KLHL13 | 1.739634722 | 0.798784409 |
| ENSG00000138594 | TMOD3 | 1.739553519 | 0.798717066 |
| ENSG00000120306 | CYSTM1 | 1.73951255 | 0.798683088 |
| ENSG00000267335 | AC008687.1 | 1.73895 | 0.798216451 |
| ENSG00000148730 | EIF4EBP2 | 1.738678602 | 0.797991272 |
| ENSG00000096746 | HNRNPH3 | 1.738489724 | 0.797834539 |
| ENSG00000125977 | EIF2S2 | 1.73801894 | 0.797443804 |
| ENSG00000128394 | APOBEC3F | 1.737892279 | 0.797338661 |
| ENSG00000054523 | KIF1B | 1.737855036 | 0.797307744 |
| ENSG00000151090 | THRB | 1.737753739 | 0.797223649 |
| ENSG00000118777 | ABCG2 | 1.737636918 | 0.797126661 |
| ENSG00000176490 | DIRAS1 | 1.737593196 | 0.797090359 |
| ENSG00000177873 | ZNF619 | 1.737494769 | 0.797008634 |
| ENSG00000106686 | SPATA6L | 1.737427157 | 0.796952493 |
| ENSG00000175274 | TP53I11 | 1.736753469 | 0.796392979 |
| ENSG00000176853 | FAM91A1 | 1.736416394 | 0.796112949 |
| ENSG00000119661 | DNAL1 | 1.736227059 | 0.795955632 |
| ENSG00000166575 | TMEM135 | 1.735837856 | 0.795632193 |
| ENSG00000163001 | CFAP36 | 1.73563448 | 0.795463152 |
| ENSG00000154545 | MAGED4 | 1.735511262 | 0.795360727 |
| ENSG00000274641 | HIST1H2BO | 1.73526687 | 0.795157555 |
| ENSG00000145975 | FAM217A | 1.734529556 | 0.794544424 |
| ENSG00000061918 | GUCY1B1 | 1.734516976 | 0.794533961 |
| ENSG00000092621 | PHGDH | 1.734241107 | 0.794304487 |
| ENSG00000068366 | ACSL4 | 1.733921316 | 0.794038431 |
| ENSG00000277858 | H2AFB2 | 1.733804298 | 0.793941065 |
| ENSG00000276180 | HIST1H4I | 1.733574466 | 0.793749809 |
| ENSG00000187486 | KCNJ11 | 1.733140223 | 0.793388383 |
| ENSG00000165782 | PIP4P1 | 1.733120109 | 0.79337164 |
| ENSG00000138646 | HERC5 | 1.73309377 | 0.793349714 |
| ENSG00000150782 | IL18 | 1.73294 | 0.793221705 |
| ENSG00000155085 | AK9 | 1.732875777 | 0.793168237 |
| ENSG00000157653 | C9orf43 | 1.732122661 | 0.792541099 |
| ENSG00000112685 | EXOC2 | 1.732088604 | 0.792512732 |
| ENSG00000164258 | NDUFS4 | 1.731733361 | 0.792216812 |
| ENSG00000137714 | FDX1 | 1.731070039 | 0.791664097 |
| ENSG00000111780 | AL021546.1 | 1.72989 | 0.790680303 |
| ENSG00000183979 | NPB | 1.729603138 | 0.790441046 |
| ENSG00000100583 | SAMD15 | 1.729361439 | 0.790239426 |
| ENSG00000186222 | BLOC1S4 | 1.729048247 | 0.789978126 |
| ENSG00000120885 | CLU | 1.729032927 | 0.789965344 |
| ENSG00000150527 | MIA2 | 1.728632729 | 0.789631382 |
| ENSG00000121486 | TRMT1L | 1.728412779 | 0.789447802 |
| ENSG00000157869 | RAB28 | 1.728296127 | 0.78935043 |
| ENSG00000106537 | TSPAN13 | 1.728024822 | 0.789123941 |
| ENSG00000079950 | STX7 | 1.726930101 | 0.78820969 |
| ENSG00000164219 | PGGT1B | 1.726756459 | 0.78806462 |
| ENSG00000155313 | USP25 | 1.72661279 | 0.78794458 |
| ENSG00000163626 | COX18 | 1.726592811 | 0.787927886 |
| ENSG00000115274 | INO80B | 1.725709655 | 0.787189756 |
| ENSG00000152422 | XRCC4 | 1.725634509 | 0.787126933 |
| ENSG00000165156 | ZHX1 | 1.725622219 | 0.787116657 |
| ENSG00000270149 | AL591806.3 | 1.725417323 | 0.786945346 |
| ENSG00000115468 | EFHD1 | 1.725015871 | 0.786609636 |
| ENSG00000105388 | CEACAM5 | 1.724784863 | 0.786416422 |
| ENSG00000178057 | NDUFAF3 | 1.724761762 | 0.786397099 |
| ENSG00000187243 | MAGED4B | 1.72459402 | 0.786256783 |
| ENSG00000005108 | THSD7A | 1.724316834 | 0.786024887 |
| ENSG00000151413 | NUBPL | 1.724168481 | 0.785900758 |
| ENSG00000119471 | HSDL2 | 1.724059061 | 0.785809198 |
| ENSG00000106976 | DNM1 | 1.723167087 | 0.7850626 |
| ENSG00000180423 | HARBI1 | 1.722969577 | 0.784897228 |
| ENSG00000134897 | BIVM | 1.722349066 | 0.784377562 |
| ENSG00000196104 | SPOCK3 | 1.722145211 | 0.784206796 |
| ENSG00000131409 | LRRC4B | 1.722029537 | 0.784109889 |
| ENSG00000255292 | AP002884.3 | 1.721859273 | 0.783967237 |
| ENSG00000146676 | PURB | 1.721686153 | 0.783822177 |
| ENSG00000286261 | AC022137.3 | 1.721416108 | 0.783595874 |
| ENSG00000162194 | LBHD1 | 1.721206914 | 0.783420541 |
| ENSG00000185414 | MRPL30 | 1.72089705 | 0.783160793 |
| ENSG00000111674 | ENO2 | 1.720532382 | 0.782855045 |
| ENSG00000175482 | POLD4 | 1.719592445 | 0.782066677 |
| ENSG00000067334 | DNTTIP2 | 1.719110241 | 0.781662063 |
| ENSG00000115520 | COQ10B | 1.719052903 | 0.781613943 |
| ENSG00000075785 | RAB7A | 1.718879329 | 0.781468266 |
| ENSG00000154079 | SDHAF4 | 1.718281211 | 0.780966165 |
| ENSG00000117266 | CDK18 | 1.718020793 | 0.780747498 |
| ENSG00000115350 | POLE4 | 1.717832302 | 0.780589205 |
| ENSG00000166946 | CCNDBP1 | 1.717378372 | 0.780207928 |
| ENSG00000028277 | POU2F2 | 1.716690442 | 0.779629913 |
| ENSG00000186496 | ZNF396 | 1.716667363 | 0.779610517 |
| ENSG00000175984 | DENND2C | 1.716622586 | 0.779572885 |
| ENSG00000178814 | OPLAH | 1.715647311 | 0.778753006 |
| ENSG00000198843 | SELENOT | 1.715459191 | 0.778594806 |
| ENSG00000163993 | S100P | 1.71408 | 0.777434445 |
| ENSG00000168913 | ENHO | 1.714063504 | 0.777420561 |
| ENSG00000038210 | PI4K2B | 1.714038249 | 0.777399303 |
| ENSG00000197826 | CFAP299 | 1.713924483 | 0.777303545 |
| ENSG00000123595 | RAB9A | 1.713853762 | 0.777244014 |
| ENSG00000158747 | NBL1 | 1.713581809 | 0.77701507 |
| ENSG00000226742 | HSBP1L1 | 1.71353696 | 0.776977311 |
| ENSG00000108039 | XPNPEP1 | 1.713505251 | 0.776950613 |
| ENSG00000124678 | TCP11 | 1.713246527 | 0.776732763 |
| ENSG00000114268 | PFKFB4 | 1.713160119 | 0.776659998 |
| ENSG00000183186 | C2CD4C | 1.713077579 | 0.776590487 |
| ENSG00000179111 | HES7 | 1.712754052 | 0.776317998 |
| ENSG00000175701 | MTLN | 1.712496607 | 0.776101129 |
| ENSG00000129226 | CD68 | 1.712311537 | 0.775945208 |
| ENSG00000094841 | UPRT | 1.712154977 | 0.775813294 |
| ENSG00000168491 | CCDC110 | 1.711990561 | 0.775674747 |
| ENSG00000184611 | KCNH7 | 1.711780957 | 0.775498103 |
| ENSG00000231738 | TSPAN19 | 1.71127 | 0.775067403 |
| ENSG00000215440 | NPEPL1 | 1.710728537 | 0.774610847 |
| ENSG00000110851 | PRDM4 | 1.710510082 | 0.774426607 |
| ENSG00000100101 | Z83844.1 | 1.70912 | 0.773253694 |
| ENSG00000139266 | MARCH9 | 1.708962079 | 0.773120385 |
| ENSG00000139726 | DENR | 1.707159873 | 0.771598171 |
| ENSG00000089127 | AC004551.1 | 1.7071 | 0.771547572 |
| ENSG00000146070 | PLA2G7 | 1.70689012 | 0.771370189 |
| ENSG00000138468 | SENP7 | 1.705634664 | 0.770308664 |
| ENSG00000103404 | USP31 | 1.705343673 | 0.77006251 |
| ENSG00000100626 | GALNT16 | 1.705285778 | 0.770013531 |
| ENSG00000277224 | HIST1H2BF | 1.705262817 | 0.769994106 |
| ENSG00000159459 | UBR1 | 1.705201611 | 0.769942323 |
| ENSG00000081320 | STK17B | 1.705147478 | 0.769896523 |
| ENSG00000138032 | PPM1B | 1.705144242 | 0.769893785 |
| ENSG00000144036 | EXOC6B | 1.704960861 | 0.769738621 |
| ENSG00000198723 | TEX45 | 1.704753591 | 0.769563224 |
| ENSG00000104313 | EYA1 | 1.703859595 | 0.768806457 |
| ENSG00000243317 | STMP1 | 1.70358115 | 0.768570672 |
| ENSG00000136279 | DBNL | 1.703493021 | 0.768496037 |
| ENSG00000156504 | FAM122B | 1.70322845 | 0.768271953 |
| ENSG00000129451 | KLK10 | 1.70312 | 0.768180089 |
| ENSG00000182108 | DEXI | 1.702963732 | 0.76804771 |
| ENSG00000146833 | TRIM4 | 1.702012494 | 0.767241628 |
| ENSG00000173812 | EIF1 | 1.701883676 | 0.767132432 |
| ENSG00000144566 | RAB5A | 1.701802007 | 0.7670632 |
| ENSG00000164674 | SYTL3 | 1.701641325 | 0.766926975 |
| ENSG00000105088 | OLFM2 | 1.701374963 | 0.766701129 |
| ENSG00000141664 | ZCCHC2 | 1.700478975 | 0.765941168 |
| ENSG00000165118 | C9orf64 | 1.700415205 | 0.765887065 |
| ENSG00000188290 | HES4 | 1.700123692 | 0.765639713 |
| ENSG00000136536 | MARCH7 | 1.699815443 | 0.765378115 |
| ENSG00000112406 | HECA | 1.699619025 | 0.765211398 |
| ENSG00000112276 | BVES | 1.698502833 | 0.764263625 |
| ENSG00000107175 | CREB3 | 1.698484473 | 0.76424803 |
| ENSG00000175183 | CSRP2 | 1.698123369 | 0.763941275 |
| ENSG00000223638 | RFPL4A | 1.6972953 | 0.763237591 |
| ENSG00000010610 | CD4 | 1.696591899 | 0.762639578 |
| ENSG00000165626 | BEND7 | 1.69592595 | 0.762073178 |
| ENSG00000184227 | ACOT1 | 1.695766515 | 0.761937544 |
| ENSG00000154760 | SLFN13 | 1.695732543 | 0.761908641 |
| ENSG00000205138 | SDHAF1 | 1.695533296 | 0.761739115 |
| ENSG00000177868 | SVBP | 1.6953097 | 0.761548849 |
| ENSG00000138785 | INTS12 | 1.695010792 | 0.761294459 |
| ENSG00000180998 | GPR137C | 1.694976874 | 0.76126559 |
| ENSG00000205413 | SAMD9 | 1.694692695 | 0.761023687 |
| ENSG00000145861 | C1QTNF2 | 1.694360757 | 0.760741081 |
| ENSG00000137968 | SLC44A5 | 1.694197062 | 0.760601693 |
| ENSG00000099617 | EFNA2 | 1.694009406 | 0.760441886 |
| ENSG00000171848 | RRM2 | 1.693902636 | 0.760350952 |
| ENSG00000115944 | COX7A2L | 1.693347559 | 0.759878116 |
| ENSG00000196440 | ARMCX4 | 1.693086521 | 0.759655701 |
| ENSG00000196814 | MVB12B | 1.693085557 | 0.759654879 |
| ENSG00000166848 | TERF2IP | 1.693040622 | 0.759616589 |
| ENSG00000183644 | C11orf88 | 1.692676753 | 0.759306491 |
| ENSG00000168904 | LRRC28 | 1.692667556 | 0.759298652 |
| ENSG00000204335 | SP5 | 1.69197922 | 0.75871185 |
| ENSG00000277745 | H2AFB3 | 1.691966277 | 0.758700814 |
| ENSG00000173083 | HPSE | 1.691710709 | 0.758482882 |
| ENSG00000011201 | ANOS1 | 1.691449382 | 0.758260004 |
| ENSG00000174446 | SNAPC5 | 1.691137393 | 0.757993874 |
| ENSG00000185043 | CIB1 | 1.690645779 | 0.757574421 |
| ENSG00000170917 | NUDT6 | 1.69014999 | 0.757151282 |
| ENSG00000135968 | GCC2 | 1.689953757 | 0.75698377 |
| ENSG00000109079 | TNFAIP1 | 1.689951008 | 0.756981423 |
| ENSG00000165113 | GKAP1 | 1.689741047 | 0.75680217 |
| ENSG00000134222 | PSRC1 | 1.689403576 | 0.75651401 |
| ENSG00000144048 | DUSP11 | 1.689334338 | 0.756454882 |
| ENSG00000174516 | PELI3 | 1.688577206 | 0.755808145 |
| ENSG00000160209 | PDXK | 1.688104295 | 0.75540404 |
| ENSG00000145945 | FAM50B | 1.687643009 | 0.755009759 |
| ENSG00000134827 | TCN1 | 1.68758 | 0.754955895 |
| ENSG00000126581 | BECN1 | 1.687570496 | 0.75494777 |
| ENSG00000164111 | ANXA5 | 1.687228719 | 0.754655557 |
| ENSG00000105939 | ZC3HAV1 | 1.6860303 | 0.753630463 |
| ENSG00000180448 | AC004151.1 | 1.685896385 | 0.753515871 |
| ENSG00000277586 | NEFL | 1.685385947 | 0.753079001 |
| ENSG00000161798 | AQP5 | 1.68493 | 0.752688656 |
| ENSG00000240204 | SMKR1 | 1.684806634 | 0.752583022 |
| ENSG00000143178 | TBX19 | 1.684674395 | 0.752469782 |
| ENSG00000082068 | WDR70 | 1.684542616 | 0.752356927 |
| ENSG00000100439 | ABHD4 | 1.684359762 | 0.752200316 |
| ENSG00000178922 | HYI | 1.684251592 | 0.752107663 |
| ENSG00000103260 | METRN | 1.684043926 | 0.75192977 |
| ENSG00000122912 | SLC25A16 | 1.683552754 | 0.751508928 |
| ENSG00000119121 | TRPM6 | 1.683413696 | 0.75138976 |
| ENSG00000119979 | FAM45A | 1.683297969 | 0.751290578 |
| ENSG00000153786 | ZDHHC7 | 1.683022568 | 0.751054523 |
| ENSG00000163159 | VPS72 | 1.68293348 | 0.750978154 |
| ENSG00000197147 | LRRC8B | 1.682649521 | 0.750734709 |
| ENSG00000130751 | NPAS1 | 1.682375555 | 0.750499793 |
| ENSG00000090615 | GOLGA3 | 1.681115119 | 0.749418521 |
| ENSG00000175387 | SMAD2 | 1.680730838 | 0.749088702 |
| ENSG00000141556 | TBCD | 1.680640709 | 0.749011335 |
| ENSG00000078967 | UBE2D4 | 1.680509861 | 0.748899009 |
| ENSG00000175198 | PCCA | 1.680322904 | 0.748738499 |
| ENSG00000155368 | DBI | 1.679652996 | 0.748163213 |
| ENSG00000157110 | RBPMS | 1.679591779 | 0.748110632 |
| ENSG00000198932 | GPRASP1 | 1.679473023 | 0.748008622 |
| ENSG00000203760 | CENPW | 1.679239818 | 0.747808282 |
| ENSG00000160712 | IL6R | 1.679203224 | 0.747776842 |
| ENSG00000100216 | TOMM22 | 1.679148035 | 0.747729425 |
| ENSG00000101557 | USP14 | 1.679052703 | 0.747647515 |
| ENSG00000160179 | ABCG1 | 1.679025737 | 0.747624345 |
| ENSG00000102531 | FNDC3A | 1.67901643 | 0.747616348 |
| ENSG00000086506 | HBQ1 | 1.678985327 | 0.747589622 |
| ENSG00000100592 | DAAM1 | 1.678896796 | 0.747513549 |
| ENSG00000184905 | TCEAL2 | 1.67882 | 0.747447555 |
| ENSG00000171703 | TCEA2 | 1.678707979 | 0.747351287 |
| ENSG00000185010 | F8 | 1.678490082 | 0.747164012 |
| ENSG00000074935 | TUBE1 | 1.678448754 | 0.74712849 |
| ENSG00000166183 | ASPG | 1.678439703 | 0.74712071 |
| ENSG00000101489 | CELF4 | 1.6781733 | 0.746891706 |
| ENSG00000188958 | UTS2B | 1.678162381 | 0.746882319 |
| ENSG00000205268 | PDE7A | 1.677887258 | 0.74664578 |
| ENSG00000153814 | JAZF1 | 1.677566155 | 0.746369661 |
| ENSG00000115129 | TP53I3 | 1.677437874 | 0.746259335 |
| ENSG00000139910 | NOVA1 | 1.677295608 | 0.746136973 |
| ENSG00000072736 | NFATC3 | 1.676831512 | 0.745737734 |
| ENSG00000114956 | DGUOK | 1.676683853 | 0.745610688 |
| ENSG00000048540 | LMO3 | 1.676630719 | 0.745564968 |
| ENSG00000182264 | IZUMO1 | 1.676278596 | 0.745261943 |
| ENSG00000269026 | AC003006.1 | 1.676179783 | 0.745176897 |
| ENSG00000174405 | LIG4 | 1.675684546 | 0.744750581 |
| ENSG00000164117 | FBXO8 | 1.67509462 | 0.74424259 |
| ENSG00000086619 | ERO1B | 1.675009201 | 0.74416902 |
| ENSG00000197343 | ZNF655 | 1.674833992 | 0.744018104 |
| ENSG00000106605 | BLVRA | 1.674668108 | 0.743875205 |
| ENSG00000163840 | DTX3L | 1.674575279 | 0.743795232 |
| ENSG00000172824 | CES4A | 1.673941639 | 0.74324923 |
| ENSG00000184515 | BEX5 | 1.673780894 | 0.743110684 |
| ENSG00000109854 | HTATIP2 | 1.673346259 | 0.742736007 |
| ENSG00000151623 | NR3C2 | 1.673248993 | 0.742652146 |
| ENSG00000172733 | PURG | 1.672681849 | 0.742163065 |
| ENSG00000077152 | UBE2T | 1.672453872 | 0.74196642 |
| ENSG00000138028 | CGREF1 | 1.672345695 | 0.741873102 |
| ENSG00000140284 | SLC27A2 | 1.67232311 | 0.741853618 |
| ENSG00000070087 | PFN2 | 1.672311113 | 0.741843269 |
| ENSG00000128524 | ATP6V1F | 1.671653451 | 0.741275795 |
| ENSG00000123178 | SPRYD7 | 1.671598858 | 0.741228678 |
| ENSG00000168275 | COA6 | 1.671561913 | 0.741196792 |
| ENSG00000276644 | DACH1 | 1.671534552 | 0.741173177 |
| ENSG00000106608 | URGCP | 1.671527124 | 0.741166766 |
| ENSG00000197837 | HIST4H4 | 1.671236045 | 0.740915514 |
| ENSG00000130720 | FIBCD1 | 1.670974613 | 0.740689815 |
| ENSG00000169018 | FEM1B | 1.670628039 | 0.740390557 |
| ENSG00000161526 | SAP30BP | 1.670005886 | 0.739853188 |
| ENSG00000177839 | PCDHB9 | 1.669176151 | 0.739136213 |
| ENSG00000102760 | RGCC | 1.66913 | 0.739096323 |
| ENSG00000079156 | OSBPL6 | 1.668900069 | 0.738897571 |
| ENSG00000166840 | GLYATL1 | 1.668838387 | 0.738844248 |
| ENSG00000114021 | NIT2 | 1.668694928 | 0.738720224 |
| ENSG00000110047 | EHD1 | 1.668194787 | 0.738287756 |
| ENSG00000161940 | BCL6B | 1.668148537 | 0.738247756 |
| ENSG00000149260 | CAPN5 | 1.667489617 | 0.737677778 |
| ENSG00000161609 | CCDC155 | 1.667059867 | 0.737305915 |
| ENSG00000101544 | ADNP2 | 1.667026254 | 0.737276825 |
| ENSG00000155115 | GTF3C6 | 1.666838409 | 0.737114249 |
| ENSG00000186862 | PDZD7 | 1.666749079 | 0.73703693 |
| ENSG00000107796 | ACTA2 | 1.666123375 | 0.736495235 |
| ENSG00000184368 | MAP7D2 | 1.666003813 | 0.736391702 |
| ENSG00000083099 | LYRM2 | 1.665848585 | 0.736257275 |
| ENSG00000160539 | PLPP7 | 1.66545833 | 0.735919258 |
| ENSG00000153132 | CLGN | 1.665437456 | 0.735901176 |
| ENSG00000158555 | GDPD5 | 1.664866748 | 0.735406712 |
| ENSG00000176531 | PHLDB3 | 1.664810288 | 0.735357786 |
| ENSG00000196372 | ASB13 | 1.664710222 | 0.735271068 |
| ENSG00000081051 | AFP | 1.66427 | 0.734889506 |
| ENSG00000167434 | CA4 | 1.6641 | 0.734742131 |
| ENSG00000063978 | RNF4 | 1.663874576 | 0.734546687 |
| ENSG00000169764 | UGP2 | 1.663598944 | 0.734307675 |
| ENSG00000205323 | SARNP | 1.663550065 | 0.734265285 |
| ENSG00000112655 | PTK7 | 1.663121448 | 0.733893524 |
| ENSG00000186522 | SEPT10 | 1.663070801 | 0.733849589 |
| ENSG00000138433 | CIR1 | 1.662987961 | 0.733777725 |
| ENSG00000214447 | FAM187A | 1.662698259 | 0.733526377 |
| ENSG00000107651 | SEC23IP | 1.662571343 | 0.73341625 |
| ENSG00000255112 | CHMP1B | 1.661558501 | 0.732537089 |
| ENSG00000168256 | NKIRAS2 | 1.661378147 | 0.732380483 |
| ENSG00000128714 | HOXD13 | 1.661314345 | 0.732325078 |
| ENSG00000159658 | EFCAB14 | 1.660208702 | 0.731364612 |
| ENSG00000151773 | CCDC122 | 1.660192745 | 0.731350745 |
| ENSG00000148225 | WDR31 | 1.660179862 | 0.73133955 |
| ENSG00000170345 | FOS | 1.660175636 | 0.731335877 |
| ENSG00000150433 | TMEM218 | 1.659092252 | 0.730394108 |
| ENSG00000162385 | MAGOH | 1.658267359 | 0.729676629 |
| ENSG00000254901 | BORCS8 | 1.658055329 | 0.729492151 |
| ENSG00000175105 | ZNF654 | 1.657869487 | 0.729330438 |
| ENSG00000272104 | Z84492.2 | 1.65721 | 0.728756431 |
| ENSG00000149609 | C20orf144 | 1.657119061 | 0.728677261 |
| ENSG00000165832 | TRUB1 | 1.656852431 | 0.728445114 |
| ENSG00000126005 | MMP24OS | 1.656555209 | 0.728186285 |
| ENSG00000268041 | AC010616.1 | 1.655560439 | 0.72731968 |
| ENSG00000111906 | HDDC2 | 1.655083364 | 0.726903885 |
| ENSG00000145687 | SSBP2 | 1.65464466 | 0.726521427 |
| ENSG00000185055 | EFCAB10 | 1.654635173 | 0.726513156 |
| ENSG00000100612 | DHRS7 | 1.654501843 | 0.726396899 |
| ENSG00000104524 | PYCR3 | 1.654403027 | 0.726310731 |
| ENSG00000144369 | FAM171B | 1.654231457 | 0.726161108 |
| ENSG00000065618 | COL17A1 | 1.654190732 | 0.72612559 |
| ENSG00000143862 | ARL8A | 1.654169066 | 0.726106694 |
| ENSG00000078618 | NRDC | 1.65414243 | 0.726083463 |
| ENSG00000130881 | LRP3 | 1.654006647 | 0.725965032 |
| ENSG00000171840 | NINJ2 | 1.653580903 | 0.725593633 |
| ENSG00000073150 | PANX2 | 1.65323901 | 0.725295312 |
| ENSG00000117139 | KDM5B | 1.653084638 | 0.725160593 |
| ENSG00000215114 | UBXN2B | 1.652973038 | 0.725063192 |
| ENSG00000099942 | CRKL | 1.652338518 | 0.724509285 |
| ENSG00000117000 | RLF | 1.65178532 | 0.724026194 |
| ENSG00000145198 | VWA5B2 | 1.650999007 | 0.723339252 |
| ENSG00000165312 | OTUD1 | 1.650921879 | 0.723271854 |
| ENSG00000103657 | HERC1 | 1.650840068 | 0.72320036 |
| ENSG00000117859 | OSBPL9 | 1.650667186 | 0.723049267 |
| ENSG00000129028 | THAP10 | 1.65066159 | 0.723044377 |
| ENSG00000145016 | RUBCN | 1.650643244 | 0.723028342 |
| ENSG00000128965 | CHAC1 | 1.650545522 | 0.722942929 |
| ENSG00000196517 | SLC6A9 | 1.650455148 | 0.722863933 |
| ENSG00000152284 | TCF7L1 | 1.650422 | 0.722834958 |
| ENSG00000112837 | TBX18 | 1.649764918 | 0.722260463 |
| ENSG00000088387 | DOCK9 | 1.6495472 | 0.72207006 |
| ENSG00000123609 | NMI | 1.649537105 | 0.72206123 |
| ENSG00000130244 | FAM98C | 1.649488496 | 0.722018716 |
| ENSG00000143590 | EFNA3 | 1.648813623 | 0.72142833 |
| ENSG00000272886 | DCP1A | 1.648581076 | 0.72122484 |
| ENSG00000159753 | CARMIL2 | 1.648579489 | 0.721223451 |
| ENSG00000115902 | SLC1A4 | 1.648388368 | 0.721056188 |
| ENSG00000205531 | NAP1L4 | 1.648191938 | 0.72088426 |
| ENSG00000119650 | IFT43 | 1.64778342 | 0.720526631 |
| ENSG00000004975 | DVL2 | 1.647729985 | 0.720479846 |
| ENSG00000074319 | TSG101 | 1.647726462 | 0.720476762 |
| ENSG00000197238 | HIST1H4J | 1.647672087 | 0.720429152 |
| ENSG00000176473 | WDR25 | 1.647493081 | 0.720272406 |
| ENSG00000136925 | TSTD2 | 1.647444284 | 0.720229674 |
| ENSG00000256060 | TRAPPC2B | 1.64673032 | 0.719604309 |
| ENSG00000108509 | CAMTA2 | 1.645995241 | 0.718960165 |
| ENSG00000215717 | TMEM167B | 1.645523651 | 0.718546762 |
| ENSG00000138674 | SEC31A | 1.645146665 | 0.718216207 |
| ENSG00000162627 | SNX7 | 1.645139544 | 0.718209962 |
| ENSG00000159720 | ATP6V0D1 | 1.644947854 | 0.71804185 |
| ENSG00000143952 | VPS54 | 1.644789393 | 0.717902866 |
| ENSG00000062725 | APPBP2 | 1.644787332 | 0.717901058 |
| ENSG00000128340 | RAC2 | 1.6447 | 0.717824454 |
| ENSG00000197747 | S100A10 | 1.644411685 | 0.717571528 |
| ENSG00000112186 | CAP2 | 1.644218695 | 0.717402202 |
| ENSG00000179399 | GPC5 | 1.644193138 | 0.717379777 |
| ENSG00000188486 | H2AFX | 1.644186124 | 0.717373623 |
| ENSG00000213977 | TAX1BP3 | 1.643844942 | 0.717074221 |
| ENSG00000083799 | CYLD | 1.643612973 | 0.716870623 |
| ENSG00000161202 | DVL3 | 1.643463123 | 0.716739085 |
| ENSG00000172216 | CEBPB | 1.64279677 | 0.716154016 |
| ENSG00000266964 | FXYD1 | 1.642638352 | 0.716014887 |
| ENSG00000178764 | ZHX2 | 1.642454343 | 0.715853267 |
| ENSG00000198954 | KIF1BP | 1.642119057 | 0.715558729 |
| ENSG00000120860 | WASHC3 | 1.642056704 | 0.715503947 |
| ENSG00000105357 | MYH14 | 1.641729258 | 0.715216228 |
| ENSG00000162378 | ZYG11B | 1.641533192 | 0.715043922 |
| ENSG00000213996 | TM6SF2 | 1.64136044 | 0.714892087 |
| ENSG00000135148 | TRAFD1 | 1.641289636 | 0.714829852 |
| ENSG00000121871 | SLITRK3 | 1.641017626 | 0.714590734 |
| ENSG00000112773 | TENT5A | 1.640973359 | 0.714551817 |
| ENSG00000196998 | WDR45 | 1.64061603 | 0.714237629 |
| ENSG00000137720 | C11orf1 | 1.6403643 | 0.714016251 |
| ENSG00000102362 | SYTL4 | 1.639218148 | 0.713007861 |
| ENSG00000151500 | THYN1 | 1.639074481 | 0.712881413 |
| ENSG00000074317 | SNCB | 1.638821518 | 0.712658741 |
| ENSG00000100335 | MIEF1 | 1.638338291 | 0.712233282 |
| ENSG00000122042 | UBL3 | 1.63825618 | 0.712160974 |
| ENSG00000143226 | FCGR2A | 1.638173705 | 0.712088342 |
| ENSG00000083838 | ZNF446 | 1.637961054 | 0.711901054 |
| ENSG00000099246 | RAB18 | 1.637902931 | 0.711849859 |
| ENSG00000136897 | MRPL50 | 1.637770618 | 0.711733311 |
| ENSG00000153310 | FAM49B | 1.637756181 | 0.711720593 |
| ENSG00000157800 | SLC37A3 | 1.637735123 | 0.711702043 |
| ENSG00000196275 | GTF2IRD2 | 1.637599104 | 0.711582218 |
| ENSG00000138663 | COPS4 | 1.637458931 | 0.711458723 |
| ENSG00000122592 | HOXA7 | 1.636763921 | 0.71084625 |
| ENSG00000128573 | FOXP2 | 1.636243557 | 0.710387511 |
| ENSG00000136213 | CHST12 | 1.636133184 | 0.710290191 |
| ENSG00000166484 | MAPK7 | 1.636011291 | 0.710182705 |
| ENSG00000041880 | PARP3 | 1.635692302 | 0.709901381 |
| ENSG00000166888 | STAT6 | 1.635615359 | 0.709833516 |
| ENSG00000151240 | DIP2C | 1.635612509 | 0.709831002 |
| ENSG00000134698 | AGO4 | 1.635151269 | 0.709424106 |
| ENSG00000141582 | CBX4 | 1.634964649 | 0.709259442 |
| ENSG00000142892 | PIGK | 1.634871378 | 0.709177138 |
| ENSG00000159082 | SYNJ1 | 1.63463072 | 0.708964752 |
| ENSG00000196152 | ZNF79 | 1.634591527 | 0.708930162 |
| ENSG00000198055 | GRK6 | 1.63413544 | 0.708527562 |
| ENSG00000188001 | TPRG1 | 1.633995723 | 0.708404207 |
| ENSG00000171121 | KCNMB3 | 1.633736669 | 0.708175464 |
| ENSG00000129116 | PALLD | 1.633573387 | 0.708031269 |
| ENSG00000104825 | NFKBIB | 1.633560239 | 0.708019656 |
| ENSG00000145362 | ANK2 | 1.633358702 | 0.707841656 |
| ENSG00000124116 | WFDC3 | 1.633259709 | 0.707754216 |
| ENSG00000174428 | GTF2IRD2B | 1.633155458 | 0.707662125 |
| ENSG00000233757 | AC092835.1 | 1.632712088 | 0.707270409 |
| ENSG00000104979 | C19orf53 | 1.632682852 | 0.707244575 |
| ENSG00000112182 | BACH2 | 1.632063075 | 0.706696815 |
| ENSG00000134153 | EMC7 | 1.632022765 | 0.706661181 |
| ENSG00000168092 | PAFAH1B2 | 1.631917561 | 0.706568179 |
| ENSG00000180479 | ZNF571 | 1.631813516 | 0.706476195 |
| ENSG00000166801 | FAM111A | 1.631758605 | 0.706427647 |
| ENSG00000205456 | TP53TG3D | 1.631715521 | 0.706389555 |
| ENSG00000108587 | GOSR1 | 1.631337314 | 0.706055121 |
| ENSG00000137601 | NEK1 | 1.63101924 | 0.705773801 |
| ENSG00000198453 | ZNF568 | 1.630843336 | 0.705618199 |
| ENSG00000251369 | ZNF550 | 1.629997179 | 0.704869467 |
| ENSG00000158828 | PINK1 | 1.629392274 | 0.704333972 |
| ENSG00000182504 | CEP97 | 1.629285238 | 0.704239197 |
| ENSG00000089177 | KIF16B | 1.628648043 | 0.703674866 |
| ENSG00000104081 | BMF | 1.628584037 | 0.703618167 |
| ENSG00000164078 | MST1R | 1.628572053 | 0.70360755 |
| ENSG00000174808 | BTC | 1.628542647 | 0.7035815 |
| ENSG00000197114 | ZGPAT | 1.628511318 | 0.703553746 |
| ENSG00000137494 | ANKRD42 | 1.628421095 | 0.703473815 |
| ENSG00000165997 | ARL5B | 1.628318038 | 0.703382509 |
| ENSG00000270170 | NCBP2AS2 | 1.627473174 | 0.702633763 |
| ENSG00000132432 | SEC61G | 1.627359834 | 0.702533288 |
| ENSG00000021826 | CPS1 | 1.627332795 | 0.702509317 |
| ENSG00000152952 | PLOD2 | 1.627196281 | 0.702388287 |
| ENSG00000188766 | SPRED3 | 1.627160653 | 0.702356698 |
| ENSG00000100601 | ALKBH1 | 1.627021832 | 0.70223361 |
| ENSG00000267855 | NDUFA7 | 1.626659027 | 0.701911871 |
| ENSG00000054356 | PTPRN | 1.626590465 | 0.701851062 |
| ENSG00000110619 | CARS | 1.626546814 | 0.701812346 |
| ENSG00000101782 | RIOK3 | 1.626327508 | 0.701617815 |
| ENSG00000122218 | COPA | 1.626251566 | 0.701550446 |
| ENSG00000277157 | HIST1H4D | 1.625877021 | 0.701218138 |
| ENSG00000198855 | FICD | 1.625801002 | 0.701150683 |
| ENSG00000152061 | RABGAP1L | 1.625697609 | 0.701058931 |
| ENSG00000105220 | GPI | 1.625685983 | 0.701048614 |
| ENSG00000047346 | FAM214A | 1.62554499 | 0.700923486 |
| ENSG00000120333 | MRPS14 | 1.62548442 | 0.700869728 |
| ENSG00000048707 | VPS13D | 1.625417923 | 0.700810707 |
| ENSG00000239713 | APOBEC3G | 1.625295081 | 0.700701671 |
| ENSG00000023572 | GLRX2 | 1.62519141 | 0.700609644 |
| ENSG00000085982 | USP40 | 1.625133449 | 0.700558191 |
| ENSG00000136153 | LMO7 | 1.624781796 | 0.700245981 |
| ENSG00000204815 | TTC25 | 1.624363931 | 0.699874898 |
| ENSG00000107242 | PIP5K1B | 1.624262122 | 0.699784472 |
| ENSG00000108830 | RND2 | 1.624235268 | 0.69976062 |
| ENSG00000150628 | SPATA4 | 1.62402051 | 0.699569852 |
| ENSG00000223802 | CERS1 | 1.623730441 | 0.699312148 |
| ENSG00000188833 | ENTPD8 | 1.62356414 | 0.69916438 |
| ENSG00000101166 | PRELID3B | 1.623466326 | 0.699077461 |
| ENSG00000282988 | AL031777.3 | 1.623349741 | 0.698973854 |
| ENSG00000256500 | AL139300.1 | 1.6232 | 0.69884077 |
| ENSG00000203499 | IQANK1 | 1.623182719 | 0.69882541 |
| ENSG00000185361 | TNFAIP8L1 | 1.623090662 | 0.698743587 |
| ENSG00000232119 | MCTS1 | 1.622874841 | 0.698551741 |
| ENSG00000197056 | ZMYM1 | 1.622811071 | 0.69849505 |
| ENSG00000125337 | KIF25 | 1.622765136 | 0.698454213 |
| ENSG00000165821 | SALL2 | 1.622689339 | 0.698386825 |
| ENSG00000100299 | ARSA | 1.622551592 | 0.698264352 |
| ENSG00000086300 | SNX10 | 1.622445752 | 0.698170241 |
| ENSG00000163349 | HIPK1 | 1.622329269 | 0.69806666 |
| ENSG00000180488 | MIGA1 | 1.622093322 | 0.697856823 |
| ENSG00000189067 | LITAF | 1.621799396 | 0.697595381 |
| ENSG00000168944 | CEP120 | 1.621454106 | 0.69728819 |
| ENSG00000131100 | ATP6V1E1 | 1.621395375 | 0.697235933 |
| ENSG00000158062 | UBXN11 | 1.621117708 | 0.696988848 |
| ENSG00000120451 | SNX19 | 1.620934396 | 0.696825702 |
| ENSG00000064763 | FAR2 | 1.620651901 | 0.696574248 |
| ENSG00000143919 | CAMKMT | 1.620508676 | 0.696446745 |
| ENSG00000168806 | LCMT2 | 1.620456935 | 0.69640068 |
| ENSG00000125744 | RTN2 | 1.618763807 | 0.694892498 |
| ENSG00000007968 | E2F2 | 1.618728089 | 0.694860665 |
| ENSG00000105197 | TIMM50 | 1.618123603 | 0.694321815 |
| ENSG00000101558 | VAPA | 1.617920171 | 0.694140427 |
| ENSG00000119616 | FCF1 | 1.617875588 | 0.694100671 |
| ENSG00000148834 | GSTO1 | 1.617630234 | 0.693881867 |
| ENSG00000153721 | CNKSR3 | 1.617521725 | 0.693785089 |
| ENSG00000177150 | FAM210A | 1.617515245 | 0.69377931 |
| ENSG00000158234 | FAIM | 1.617484955 | 0.693752293 |
| ENSG00000089820 | ARHGAP4 | 1.617343785 | 0.693626373 |
| ENSG00000076706 | AP002956.1 | 1.617088893 | 0.693398987 |
| ENSG00000167536 | DHRS13 | 1.616954699 | 0.69327926 |
| ENSG00000168569 | TMEM223 | 1.616707244 | 0.693058457 |
| ENSG00000172508 | CARNS1 | 1.616008118 | 0.692434446 |
| ENSG00000196116 | TDRD7 | 1.616006764 | 0.692433237 |
| ENSG00000101746 | NOL4 | 1.615972417 | 0.692402573 |
| ENSG00000148248 | SURF4 | 1.615769645 | 0.692221533 |
| ENSG00000163513 | TGFBR2 | 1.615703251 | 0.692162249 |
| ENSG00000105849 | TWISTNB | 1.615610499 | 0.692079426 |
| ENSG00000214954 | LRRC69 | 1.615470533 | 0.691954435 |
| ENSG00000120725 | SIL1 | 1.614912624 | 0.691456109 |
| ENSG00000184436 | THAP7 | 1.614802002 | 0.691357281 |
| ENSG00000172572 | PDE3A | 1.613785686 | 0.690448999 |
| ENSG00000108852 | MPP2 | 1.61298027 | 0.689728791 |
| ENSG00000164244 | PRRC1 | 1.612890592 | 0.689648579 |
| ENSG00000167103 | PIP5KL1 | 1.612727912 | 0.689503057 |
| ENSG00000134339 | SAA2 | 1.61269 | 0.689469142 |
| ENSG00000115239 | ASB3 | 1.612553237 | 0.689346791 |
| ENSG00000005073 | HOXA11 | 1.611463891 | 0.688371861 |
| ENSG00000100883 | SRP54 | 1.611349797 | 0.688269713 |
| ENSG00000132141 | CCT6B | 1.611212133 | 0.688146452 |
| ENSG00000027847 | B4GALT7 | 1.611186093 | 0.688123136 |
| ENSG00000250565 | ATP6V1E2 | 1.610716539 | 0.687702624 |
| ENSG00000158560 | DYNC1I1 | 1.610047531 | 0.68710328 |
| ENSG00000111799 | COL12A1 | 1.610009217 | 0.687068947 |
| ENSG00000204052 | LRRC73 | 1.609991951 | 0.687053476 |
| ENSG00000082213 | C5orf22 | 1.60974932 | 0.686836041 |
| ENSG00000214193 | SH3D21 | 1.609500918 | 0.6866134 |
| ENSG00000095787 | WAC | 1.609067894 | 0.686225202 |
| ENSG00000286015 | CR589904.2 | 1.609026836 | 0.686188388 |
| ENSG00000162817 | C1orf115 | 1.608935507 | 0.686106498 |
| ENSG00000258881 | AC007040.2 | 1.608891764 | 0.686067274 |
| ENSG00000122068 | FYTTD1 | 1.608771363 | 0.685959306 |
| ENSG00000186472 | PCLO | 1.608765806 | 0.685954323 |
| ENSG00000014123 | UFL1 | 1.608592137 | 0.685798573 |
| ENSG00000263020 | AL662899.2 | 1.608413478 | 0.685638331 |
| ENSG00000186897 | C1QL4 | 1.608146541 | 0.685398877 |
| ENSG00000164398 | ACSL6 | 1.607460655 | 0.684783426 |
| ENSG00000177889 | UBE2N | 1.607367378 | 0.684699708 |
| ENSG00000115993 | TRAK2 | 1.607332513 | 0.684668414 |
| ENSG00000151348 | EXT2 | 1.606997133 | 0.684367355 |
| ENSG00000002330 | BAD | 1.606911019 | 0.684290043 |
| ENSG00000082146 | STRADB | 1.606774231 | 0.684167229 |
| ENSG00000187688 | TRPV2 | 1.606546289 | 0.683962549 |
| ENSG00000106615 | RHEB | 1.606286666 | 0.683729386 |
| ENSG00000116141 | MARK1 | 1.606105785 | 0.683566918 |
| ENSG00000219626 | FAM228B | 1.605468604 | 0.682994452 |
| ENSG00000160055 | TMEM234 | 1.60545203 | 0.682979559 |
| ENSG00000035687 | ADSS | 1.605286523 | 0.682830823 |
| ENSG00000213638 | ADAT3 | 1.60504388 | 0.68261274 |
| ENSG00000196465 | MYL6B | 1.604979376 | 0.682554759 |
| ENSG00000164855 | TMEM184A | 1.60481975 | 0.682411266 |
| ENSG00000141519 | CCDC40 | 1.604783286 | 0.682378485 |
| ENSG00000102003 | SYP | 1.604716177 | 0.682318153 |
| ENSG00000166311 | SMPD1 | 1.604578459 | 0.682194335 |
| ENSG00000100354 | TNRC6B | 1.604306717 | 0.681949988 |
| ENSG00000286075 | AC009412.1 | 1.60378307 | 0.681479014 |
| ENSG00000184613 | NELL2 | 1.603629993 | 0.681341306 |
| ENSG00000107625 | DDX50 | 1.60337823 | 0.681114791 |
| ENSG00000167123 | CERCAM | 1.603185677 | 0.680941525 |
| ENSG00000104967 | NOVA2 | 1.602920401 | 0.680702785 |
| ENSG00000196388 | INCA1 | 1.602904738 | 0.680688688 |
| ENSG00000106330 | MOSPD3 | 1.602889198 | 0.680674701 |
| ENSG00000073331 | ALPK1 | 1.602887697 | 0.68067335 |
| ENSG00000055130 | CUL1 | 1.602643222 | 0.680453291 |
| ENSG00000109381 | ELF2 | 1.602511171 | 0.680334413 |
| ENSG00000099957 | P2RX6 | 1.60113306 | 0.679093206 |
| ENSG00000130150 | MOSPD2 | 1.600790656 | 0.678784651 |
| ENSG00000164898 | FMC1 | 1.600705716 | 0.678708098 |
| ENSG00000170571 | EMB | 1.600334979 | 0.678373918 |
| ENSG00000126945 | HNRNPH2 | 1.600041358 | 0.678109197 |
| ENSG00000264343 | NOTCH2NLA | 1.599996905 | 0.678069114 |
| ENSG00000165487 | MICU2 | 1.599782768 | 0.677876017 |
| ENSG00000178690 | DYNAP | 1.5997 | 0.677801374 |
| ENSG00000205089 | CCNI2 | 1.599160865 | 0.677315071 |
| ENSG00000196154 | S100A4 | 1.599141148 | 0.677297284 |
| ENSG00000169432 | SCN9A | 1.598945889 | 0.677121116 |
| ENSG00000083844 | ZNF264 | 1.598911758 | 0.67709032 |
| ENSG00000177606 | JUN | 1.598836994 | 0.677022859 |
| ENSG00000204272 | NBDY | 1.598753572 | 0.676947582 |
| ENSG00000188266 | HYKK | 1.598505196 | 0.676723433 |
| ENSG00000120694 | HSPH1 | 1.598278628 | 0.676518935 |
| ENSG00000052126 | PLEKHA5 | 1.597982234 | 0.676251369 |
| ENSG00000189241 | TSPYL1 | 1.597783 | 0.676071484 |
| ENSG00000197557 | TTC30A | 1.597666378 | 0.675966178 |
| ENSG00000104177 | MYEF2 | 1.597617222 | 0.67592179 |
| ENSG00000214517 | PPME1 | 1.597566452 | 0.675875942 |
| ENSG00000128266 | GNAZ | 1.597400342 | 0.675725927 |
| ENSG00000177853 | ZNF518A | 1.596801885 | 0.675185329 |
| ENSG00000268655 | AC008687.4 | 1.596800732 | 0.675184287 |
| ENSG00000025039 | RRAGD | 1.596562796 | 0.674969298 |
| ENSG00000286019 | AC239811.1 | 1.596162277 | 0.674607333 |
| ENSG00000166816 | LDHD | 1.595805169 | 0.674284525 |
| ENSG00000136052 | SLC41A2 | 1.595669419 | 0.674161794 |
| ENSG00000147231 | RADX | 1.595405297 | 0.673922973 |
| ENSG00000205436 | EXOC3L4 | 1.59537 | 0.673891054 |
| ENSG00000244405 | ETV5 | 1.595365242 | 0.673886752 |
| ENSG00000169583 | CLIC3 | 1.595021678 | 0.673576032 |
| ENSG00000161277 | THAP8 | 1.594968536 | 0.673527964 |
| ENSG00000166896 | ATP23 | 1.594925469 | 0.673489008 |
| ENSG00000130703 | OSBPL2 | 1.594691628 | 0.673277472 |
| ENSG00000125995 | ROMO1 | 1.594667939 | 0.67325604 |
| ENSG00000159228 | CBR1 | 1.594504138 | 0.673107842 |
| ENSG00000176393 | RNPEP | 1.594467153 | 0.673074377 |
| ENSG00000112576 | CCND3 | 1.594348661 | 0.672967161 |
| ENSG00000101210 | EEF1A2 | 1.594072702 | 0.672717429 |
| ENSG00000166257 | SCN3B | 1.594055697 | 0.672702039 |
| ENSG00000131018 | SYNE1 | 1.593948258 | 0.672604798 |
| ENSG00000176871 | WSB2 | 1.593818657 | 0.672487491 |
| ENSG00000028116 | VRK2 | 1.593383529 | 0.672093567 |
| ENSG00000032742 | IFT88 | 1.593341641 | 0.67205564 |
| ENSG00000175470 | PPP2R2D | 1.593332627 | 0.672047478 |
| ENSG00000203965 | EFCAB7 | 1.592953795 | 0.671704421 |
| ENSG00000204920 | ZNF155 | 1.59291954 | 0.671673397 |
| ENSG00000160752 | FDPS | 1.592872856 | 0.671631114 |
| ENSG00000128272 | ATF4 | 1.592769883 | 0.671537847 |
| ENSG00000006740 | ARHGAP44 | 1.592208962 | 0.671029688 |
| ENSG00000164039 | BDH2 | 1.592122536 | 0.670951375 |
| ENSG00000133315 | MACROD1 | 1.591820001 | 0.670677209 |
| ENSG00000113389 | NPR3 | 1.591796071 | 0.67065552 |
| ENSG00000185608 | MRPL40 | 1.591486282 | 0.670374722 |
| ENSG00000163155 | LYSMD1 | 1.591309286 | 0.670214264 |
| ENSG00000101000 | PROCR | 1.591064455 | 0.669992281 |
| ENSG00000187838 | PLSCR3 | 1.590560729 | 0.669535456 |
| ENSG00000137434 | C6orf52 | 1.590543717 | 0.669520026 |
| ENSG00000106351 | AGFG2 | 1.590498522 | 0.669479032 |
| ENSG00000182768 | NGRN | 1.59032082 | 0.669317834 |
| ENSG00000189184 | PCDH18 | 1.590253925 | 0.669257147 |
| ENSG00000169884 | WNT10B | 1.590146568 | 0.669159749 |
| ENSG00000114026 | OGG1 | 1.59007359 | 0.669093537 |
| ENSG00000135597 | REPS1 | 1.589833621 | 0.668875793 |
| ENSG00000167034 | NKX3-1 | 1.589774435 | 0.668822084 |
| ENSG00000111237 | VPS29 | 1.589744554 | 0.668794967 |
| ENSG00000117222 | RBBP5 | 1.589586576 | 0.668651594 |
| ENSG00000145794 | MEGF10 | 1.589558975 | 0.668626544 |
| ENSG00000077092 | RARB | 1.589547339 | 0.668615983 |
| ENSG00000129910 | CDH15 | 1.589516469 | 0.668587964 |
| ENSG00000259316 | AC087632.1 | 1.589353737 | 0.668440256 |
| ENSG00000141367 | CLTC | 1.589020466 | 0.668137706 |
| ENSG00000137941 | TTLL7 | 1.588366321 | 0.667543676 |
| ENSG00000160588 | MPZL3 | 1.588000252 | 0.667211142 |
| ENSG00000259494 | MRPL46 | 1.587889248 | 0.667110291 |
| ENSG00000159792 | PSKH1 | 1.587690186 | 0.666929419 |
| ENSG00000048052 | HDAC9 | 1.587576772 | 0.66682636 |
| ENSG00000139218 | SCAF11 | 1.587304605 | 0.666579009 |
| ENSG00000102316 | MAGED2 | 1.587178164 | 0.666464083 |
| ENSG00000133805 | AMPD3 | 1.587147471 | 0.666436184 |
| ENSG00000198934 | MAGEE1 | 1.586828596 | 0.666146302 |
| ENSG00000135838 | NPL | 1.586784626 | 0.666106325 |
| ENSG00000099219 | ERMP1 | 1.586585589 | 0.66592535 |
| ENSG00000096093 | EFHC1 | 1.586336281 | 0.665698635 |
| ENSG00000103811 | CTSH | 1.586081548 | 0.665466949 |
| ENSG00000005961 | ITGA2B | 1.585839272 | 0.665246558 |
| ENSG00000095209 | TMEM38B | 1.585685927 | 0.665107048 |
| ENSG00000167608 | TMC4 | 1.585567465 | 0.664999265 |
| ENSG00000109618 | SEPSECS | 1.585278126 | 0.664735973 |
| ENSG00000120008 | WDR11 | 1.584548741 | 0.664072038 |
| ENSG00000172901 | LVRN | 1.5844762 | 0.664005989 |
| ENSG00000167244 | IGF2 | 1.584325433 | 0.663868707 |
| ENSG00000106348 | IMPDH1 | 1.583972035 | 0.663546865 |
| ENSG00000178691 | SUZ12 | 1.583866505 | 0.663450744 |
| ENSG00000184500 | PROS1 | 1.583807953 | 0.66339741 |
| ENSG00000137691 | CFAP300 | 1.583792249 | 0.663383105 |
| ENSG00000070159 | PTPN3 | 1.583629244 | 0.663234614 |
| ENSG00000163812 | ZDHHC3 | 1.583604258 | 0.663211851 |
| ENSG00000180318 | ALX1 | 1.583445834 | 0.663067517 |
| ENSG00000188816 | HMX2 | 1.583225758 | 0.662866989 |
| ENSG00000128708 | HAT1 | 1.583000848 | 0.662662028 |
| ENSG00000243649 | CFB | 1.582971148 | 0.66263496 |
| ENSG00000182575 | NXPH3 | 1.582966232 | 0.66263048 |
| ENSG00000131152 | AC010531.1 | 1.58262 | 0.662314894 |
| ENSG00000131584 | ACAP3 | 1.582572447 | 0.662271545 |
| ENSG00000146267 | FAXC | 1.582208413 | 0.661939648 |
| ENSG00000182831 | C16orf72 | 1.58189438 | 0.661653277 |
| ENSG00000136478 | TEX2 | 1.581843302 | 0.661606693 |
| ENSG00000161533 | ACOX1 | 1.581614093 | 0.661397631 |
| ENSG00000135070 | ISCA1 | 1.581605146 | 0.66138947 |
| ENSG00000165795 | NDRG2 | 1.581405011 | 0.661206901 |
| ENSG00000148925 | BTBD10 | 1.58093015 | 0.660773627 |
| ENSG00000177721 | ANXA2R | 1.580806067 | 0.660660389 |
| ENSG00000253719 | ATXN7L3B | 1.580790123 | 0.660645837 |
| ENSG00000213085 | CFAP45 | 1.580149221 | 0.660060805 |
| ENSG00000275183 | LENG9 | 1.579788783 | 0.659731684 |
| ENSG00000184216 | IRAK1 | 1.579770754 | 0.659715219 |
| ENSG00000117602 | RCAN3 | 1.579698092 | 0.659648861 |
| ENSG00000072506 | HSD17B10 | 1.579585555 | 0.659546079 |
| ENSG00000156853 | ZNF689 | 1.579225635 | 0.659217314 |
| ENSG00000139323 | POC1B | 1.579188838 | 0.659183698 |
| ENSG00000132661 | NXT1 | 1.578850944 | 0.658874976 |
| ENSG00000120137 | PANK3 | 1.578770699 | 0.658801649 |
| ENSG00000171503 | ETFDH | 1.578731079 | 0.658765443 |
| ENSG00000040608 | RTN4R | 1.578709813 | 0.658746009 |
| ENSG00000161513 | FDXR | 1.578553939 | 0.658603558 |
| ENSG00000138641 | HERC3 | 1.578494992 | 0.658549684 |
| ENSG00000115271 | GCA | 1.57842115 | 0.658482193 |
| ENSG00000119888 | EPCAM | 1.578301291 | 0.658372636 |
| ENSG00000198846 | TOX | 1.578252169 | 0.658327734 |
| ENSG00000166128 | RAB8B | 1.577862038 | 0.657971068 |
| ENSG00000158716 | DUSP23 | 1.577825837 | 0.657937967 |
| ENSG00000144182 | LIPT1 | 1.577714004 | 0.657835708 |
| ENSG00000130762 | ARHGEF16 | 1.577444163 | 0.657588939 |
| ENSG00000196428 | TSC22D2 | 1.576993299 | 0.65717653 |
| ENSG00000171450 | CDK5R2 | 1.576811287 | 0.657010009 |
| ENSG00000128604 | IRF5 | 1.576769922 | 0.656972161 |
| ENSG00000113312 | TTC1 | 1.576757646 | 0.656960929 |
| ENSG00000285446 | Z84488.2 | 1.576720771 | 0.656927189 |
| ENSG00000110321 | EIF4G2 | 1.576639036 | 0.656852399 |
| ENSG00000166796 | LDHC | 1.576626807 | 0.65684121 |
| ENSG00000160321 | ZNF208 | 1.576571446 | 0.65679055 |
| ENSG00000148541 | FAM13C | 1.576394553 | 0.65662867 |
| ENSG00000005801 | ZNF195 | 1.57637028 | 0.656606455 |
| ENSG00000274183 | H2AFB1 | 1.576209427 | 0.656459235 |
| ENSG00000014641 | MDH1 | 1.575901762 | 0.656177604 |
| ENSG00000144550 | CPNE9 | 1.575628305 | 0.655927239 |
| ENSG00000146592 | CREB5 | 1.575480696 | 0.655792077 |
| ENSG00000027869 | SH2D2A | 1.575363758 | 0.655684991 |
| ENSG00000276966 | HIST1H4E | 1.575295287 | 0.655622285 |
| ENSG00000148411 | NACC2 | 1.574945162 | 0.655301597 |
| ENSG00000172264 | MACROD2 | 1.574900428 | 0.655260619 |
| ENSG00000118276 | B4GALT6 | 1.574274383 | 0.654687013 |
| ENSG00000095932 | SMIM24 | 1.57404 | 0.654472204 |
| ENSG00000160469 | BRSK1 | 1.57400495 | 0.654440078 |
| ENSG00000164088 | PPM1M | 1.573909432 | 0.654352525 |
| ENSG00000181929 | PRKAG1 | 1.57388603 | 0.654331075 |
| ENSG00000119686 | FLVCR2 | 1.573363018 | 0.653851578 |
| ENSG00000198930 | CSAG1 | 1.57327977 | 0.653775242 |
| ENSG00000139687 | RB1 | 1.573236046 | 0.653735146 |
| ENSG00000073670 | ADAM11 | 1.573040941 | 0.653556219 |
| ENSG00000181915 | ADO | 1.572412816 | 0.652980028 |
| ENSG00000136874 | STX17 | 1.572311607 | 0.652887165 |
| ENSG00000075043 | KCNQ2 | 1.572249805 | 0.652830456 |
| ENSG00000172831 | CES2 | 1.57218144 | 0.652767724 |
| ENSG00000124104 | SNX21 | 1.572096251 | 0.652689549 |
| ENSG00000179151 | EDC3 | 1.572061209 | 0.652657391 |
| ENSG00000144597 | EAF1 | 1.572051273 | 0.652648272 |
| ENSG00000166548 | TK2 | 1.571973592 | 0.652576981 |
| ENSG00000196188 | CTSE | 1.571795184 | 0.652413237 |
| ENSG00000103496 | STX4 | 1.571775269 | 0.652394957 |
| ENSG00000131165 | CHMP1A | 1.571708362 | 0.652333544 |
| ENSG00000134265 | NAPG | 1.571682109 | 0.652309445 |
| ENSG00000178028 | DMAP1 | 1.57152594 | 0.652166086 |
| ENSG00000119673 | ACOT2 | 1.571251878 | 0.651914469 |
| ENSG00000101654 | RNMT | 1.570868902 | 0.651562785 |
| ENSG00000145864 | GABRB2 | 1.570616406 | 0.651330872 |
| ENSG00000108559 | NUP88 | 1.570239651 | 0.650984761 |
| ENSG00000143771 | CNIH4 | 1.570173106 | 0.65092362 |
| ENSG00000151422 | FER | 1.569959676 | 0.650727504 |
| ENSG00000119574 | ZBTB45 | 1.569788862 | 0.650570528 |
| ENSG00000118058 | KMT2A | 1.569543888 | 0.650345371 |
| ENSG00000002586 | CD99 | 1.569468099 | 0.650275705 |
| ENSG00000173141 | MRPL57 | 1.569364078 | 0.650180083 |
| ENSG00000181135 | ZNF707 | 1.569312161 | 0.650132356 |
| ENSG00000274391 | TPTE | 1.569283792 | 0.650106275 |
| ENSG00000162433 | AK4 | 1.569209927 | 0.650038367 |
| ENSG00000204899 | MZT1 | 1.568853709 | 0.649710831 |
| ENSG00000170310 | STX8 | 1.568657029 | 0.649529956 |
| ENSG00000029993 | HMGB3 | 1.568613201 | 0.649489647 |
| ENSG00000116750 | UCHL5 | 1.568493505 | 0.649379555 |
| ENSG00000101605 | MYOM1 | 1.567377954 | 0.64835311 |
| ENSG00000129103 | SUMF2 | 1.567284986 | 0.648267535 |
| ENSG00000073756 | PTGS2 | 1.567264916 | 0.64824906 |
| ENSG00000132763 | MMACHC | 1.567185511 | 0.648175965 |
| ENSG00000015153 | YAF2 | 1.566804026 | 0.647824741 |
| ENSG00000105617 | LENG1 | 1.566803965 | 0.647824684 |
| ENSG00000164695 | CHMP4C | 1.56646539 | 0.647512895 |
| ENSG00000244731 | C4A | 1.56618116 | 0.647251099 |
| ENSG00000137561 | TTPA | 1.566163678 | 0.647234995 |
| ENSG00000119718 | EIF2B2 | 1.566084344 | 0.647161914 |
| ENSG00000054598 | FOXC1 | 1.565893688 | 0.646986268 |
| ENSG00000152503 | TRIM36 | 1.565391587 | 0.646523596 |
| ENSG00000243943 | ZNF512 | 1.565327923 | 0.646464921 |
| ENSG00000176624 | MEX3C | 1.565301629 | 0.646440687 |
| ENSG00000213782 | DDX47 | 1.565023195 | 0.64618404 |
| ENSG00000011275 | RNF216 | 1.564841858 | 0.646016866 |
| ENSG00000189052 | CGB5 | 1.564595863 | 0.645790056 |
| ENSG00000186153 | WWOX | 1.564423621 | 0.645631225 |
| ENSG00000234545 | FAM133B | 1.564282983 | 0.645501523 |
| ENSG00000087884 | AAMDC | 1.563984567 | 0.645226276 |
| ENSG00000104427 | ZC2HC1A | 1.563957512 | 0.64520132 |
| ENSG00000108641 | B9D1 | 1.56389207 | 0.64514095 |
| ENSG00000070778 | AL162171.1 | 1.563888065 | 0.645137256 |
| ENSG00000181638 | ZFP41 | 1.563433865 | 0.644718193 |
| ENSG00000142875 | PRKACB | 1.563430691 | 0.644715264 |
| ENSG00000109689 | STIM2 | 1.563186151 | 0.644489591 |
| ENSG00000285976 | AL135905.2 | 1.563088717 | 0.644399664 |
| ENSG00000165240 | ATP7A | 1.562955187 | 0.644276414 |
| ENSG00000185633 | NDUFA4L2 | 1.562929403 | 0.644252614 |
| ENSG00000177679 | SRRM3 | 1.562838371 | 0.644168582 |
| ENSG00000183741 | CBX6 | 1.562533323 | 0.643886958 |
| ENSG00000164241 | C5orf63 | 1.562474855 | 0.643832972 |
| ENSG00000130940 | CASZ1 | 1.562313381 | 0.643683869 |
| ENSG00000106785 | TRIM14 | 1.562195536 | 0.643575043 |
| ENSG00000128283 | CDC42EP1 | 1.562151193 | 0.643534091 |
| ENSG00000137509 | PRCP | 1.561249652 | 0.64270125 |
| ENSG00000136866 | ZFP37 | 1.561188519 | 0.642644758 |
| ENSG00000241935 | HOGA1 | 1.561026387 | 0.642494925 |
| ENSG00000167881 | SRP68 | 1.560838489 | 0.642321259 |
| ENSG00000008869 | HEATR5B | 1.560823974 | 0.642307843 |
| ENSG00000055732 | MCOLN3 | 1.560683788 | 0.642178261 |
| ENSG00000187566 | NHLRC1 | 1.560438708 | 0.641951691 |
| ENSG00000159640 | ACE | 1.560210216 | 0.641740425 |
| ENSG00000147457 | CHMP7 | 1.560150806 | 0.641685488 |
| ENSG00000183963 | SMTN | 1.560053214 | 0.641595241 |
| ENSG00000187546 | AGMO | 1.559748014 | 0.641312973 |
| ENSG00000102471 | NDFIP2 | 1.559738839 | 0.641304486 |
| ENSG00000174521 | TTC9B | 1.559533947 | 0.641114957 |
| ENSG00000158792 | SPATA2L | 1.559145888 | 0.640755926 |
| ENSG00000172059 | KLF11 | 1.558729591 | 0.640370671 |
| ENSG00000186868 | MAPT | 1.558641701 | 0.640289321 |
| ENSG00000156052 | GNAQ | 1.558582163 | 0.640234211 |
| ENSG00000136247 | ZDHHC4 | 1.558414646 | 0.640079141 |
| ENSG00000138180 | CEP55 | 1.558259362 | 0.63993538 |
| ENSG00000130396 | AFDN | 1.558254258 | 0.639930655 |
| ENSG00000124920 | MYRF | 1.557655193 | 0.63937591 |
| ENSG00000113013 | HSPA9 | 1.557392655 | 0.639132727 |
| ENSG00000100902 | PSMA6 | 1.557067818 | 0.638831782 |
| ENSG00000088854 | C20orf194 | 1.556970813 | 0.6387419 |
| ENSG00000136819 | C9orf78 | 1.556825645 | 0.638607381 |
| ENSG00000159055 | MIS18A | 1.556687037 | 0.638478928 |
| ENSG00000144120 | TMEM177 | 1.556588135 | 0.638387265 |
| ENSG00000185019 | UBOX5 | 1.556504524 | 0.63830977 |
| ENSG00000113209 | PCDHB5 | 1.556343718 | 0.638160714 |
| ENSG00000181396 | OGFOD3 | 1.556182804 | 0.638011543 |
| ENSG00000075399 | VPS9D1 | 1.555430358 | 0.637313801 |
| ENSG00000089486 | CDIP1 | 1.555379713 | 0.637266827 |
| ENSG00000171174 | RBKS | 1.55504393 | 0.636955337 |
| ENSG00000165548 | TMEM63C | 1.554847422 | 0.636773015 |
| ENSG00000122481 | RWDD3 | 1.554764347 | 0.63669593 |
| ENSG00000115226 | FNDC4 | 1.554540734 | 0.63648842 |
| ENSG00000175764 | TTLL11 | 1.554349506 | 0.63631094 |
| ENSG00000166164 | BRD7 | 1.554342545 | 0.636304479 |
| ENSG00000278053 | DDX52 | 1.55422555 | 0.636195884 |
| ENSG00000119965 | C10orf88 | 1.553989658 | 0.635976902 |
| ENSG00000179085 | DPM3 | 1.553882941 | 0.635877825 |
| ENSG00000100564 | PIGH | 1.553756847 | 0.635760749 |
| ENSG00000162409 | PRKAA2 | 1.55332993 | 0.635364293 |
| ENSG00000133193 | FAM104A | 1.55283845 | 0.634907746 |
| ENSG00000179889 | PDXDC1 | 1.552782449 | 0.634855717 |
| ENSG00000162946 | DISC1 | 1.552471148 | 0.634566456 |
| ENSG00000136521 | NDUFB5 | 1.55245008 | 0.634546879 |
| ENSG00000273049 | AC012531.3 | 1.5524 | 0.634500338 |
| ENSG00000114354 | TFG | 1.552134028 | 0.63425314 |
| ENSG00000225190 | PLEKHM1 | 1.552126283 | 0.634245941 |
| ENSG00000160703 | NLRX1 | 1.552077375 | 0.634200481 |
| ENSG00000109881 | CCDC34 | 1.55205844 | 0.63418288 |
| ENSG00000185532 | PRKG1 | 1.551541133 | 0.633701945 |
| ENSG00000167157 | PRRX2 | 1.551335888 | 0.633511085 |
| ENSG00000145337 | PYURF | 1.551308174 | 0.633485312 |
| ENSG00000153714 | LURAP1L | 1.550821444 | 0.63303259 |
| ENSG00000185640 | KRT79 | 1.55081 | 0.633021943 |
| ENSG00000167525 | PROCA1 | 1.550787066 | 0.633000607 |
| ENSG00000146066 | HIGD2A | 1.550678627 | 0.632899723 |
| ENSG00000065457 | ADAT1 | 1.550449796 | 0.632686812 |
| ENSG00000168062 | BATF2 | 1.550258647 | 0.632508937 |
| ENSG00000134072 | CAMK1 | 1.550203737 | 0.632457836 |
| ENSG00000204568 | MRPS18B | 1.550013525 | 0.632280804 |
| ENSG00000139180 | NDUFA9 | 1.549554502 | 0.631853499 |
| ENSG00000168438 | CDC40 | 1.549409106 | 0.631718124 |
| ENSG00000181284 | TMEM102 | 1.549122713 | 0.631451431 |
| ENSG00000138175 | ARL3 | 1.548906941 | 0.631250469 |
| ENSG00000139734 | DIAPH3 | 1.548758227 | 0.631111946 |
| ENSG00000245848 | CEBPA | 1.548735463 | 0.63109074 |
| ENSG00000161542 | PRPSAP1 | 1.548604775 | 0.630968996 |
| ENSG00000134755 | DSC2 | 1.548507785 | 0.630878636 |
| ENSG00000134255 | CEPT1 | 1.548275906 | 0.630662586 |
| ENSG00000149212 | SESN3 | 1.548016158 | 0.63042053 |
| ENSG00000133710 | SPINK5 | 1.547823696 | 0.630241151 |
| ENSG00000120837 | NFYB | 1.547817803 | 0.630235659 |
| ENSG00000044459 | CNTLN | 1.547793459 | 0.630212968 |
| ENSG00000205795 | CYS1 | 1.547735998 | 0.630159408 |
| ENSG00000170606 | HSPA4 | 1.547656828 | 0.630085608 |
| ENSG00000156531 | PHF6 | 1.547624083 | 0.630055084 |
| ENSG00000138621 | PPCDC | 1.54732839 | 0.629779413 |
| ENSG00000187790 | FANCM | 1.547322551 | 0.629773969 |
| ENSG00000182013 | PNMA8A | 1.547246376 | 0.629702943 |
| ENSG00000156253 | RWDD2B | 1.547232667 | 0.629690161 |
| ENSG00000163932 | PRKCD | 1.54695024 | 0.629426791 |
| ENSG00000198721 | ECI2 | 1.546611525 | 0.629110868 |
| ENSG00000005020 | SKAP2 | 1.546408839 | 0.628921788 |
| ENSG00000099365 | STX1B | 1.54604073 | 0.628578327 |
| ENSG00000122884 | P4HA1 | 1.54573343 | 0.62829154 |
| ENSG00000013561 | RNF14 | 1.545715603 | 0.628274901 |
| ENSG00000165169 | DYNLT3 | 1.545442658 | 0.628020125 |
| ENSG00000131067 | GGT7 | 1.545438526 | 0.628016268 |
| ENSG00000182154 | MRPL41 | 1.545263269 | 0.627852654 |
| ENSG00000283580 | AC098484.3 | 1.545025591 | 0.627630735 |
| ENSG00000168259 | DNAJC7 | 1.544874137 | 0.627489305 |
| ENSG00000170381 | SEMA3E | 1.544783829 | 0.627404967 |
| ENSG00000187140 | FOXD3 | 1.544776959 | 0.627398551 |
| ENSG00000147894 | C9orf72 | 1.544745696 | 0.627369354 |
| ENSG00000143344 | RGL1 | 1.544720184 | 0.627345527 |
| ENSG00000183828 | NUDT14 | 1.544704361 | 0.627330749 |
| ENSG00000262576 | PCDHGA4 | 1.544643891 | 0.627274272 |
| ENSG00000085999 | RAD54L | 1.544631603 | 0.627262794 |
| ENSG00000009950 | MLXIPL | 1.54459566 | 0.627229223 |
| ENSG00000188786 | MTF1 | 1.544496965 | 0.627137036 |
| ENSG00000177675 | CD163L1 | 1.5444715 | 0.62711325 |
| ENSG00000169612 | RAMAC | 1.544367697 | 0.627016283 |
| ENSG00000148057 | IDNK | 1.544343997 | 0.626994144 |
| ENSG00000115361 | ACADL | 1.544259322 | 0.62691504 |
| ENSG00000182054 | IDH2 | 1.543618891 | 0.626316605 |
| ENSG00000124508 | BTN2A2 | 1.543586452 | 0.626286287 |
| ENSG00000103707 | MTFMT | 1.54330367 | 0.626021963 |
| ENSG00000123364 | HOXC13 | 1.54302623 | 0.625762587 |
| ENSG00000188659 | SAXO2 | 1.543000355 | 0.625738394 |
| ENSG00000178115 | GOLGA8Q | 1.542996442 | 0.625734735 |
| ENSG00000137142 | IGFBPL1 | 1.54297083 | 0.625710788 |
| ENSG00000039319 | ZFYVE16 | 1.542740505 | 0.625495415 |
| ENSG00000169548 | ZNF280A | 1.54269603 | 0.625453824 |
| ENSG00000182459 | TEX19 | 1.54259 | 0.625354664 |
| ENSG00000110046 | ATG2A | 1.54243549 | 0.625210152 |
| ENSG00000103365 | GGA2 | 1.542014635 | 0.624816458 |
| ENSG00000278318 | ZNF229 | 1.541916334 | 0.624724486 |
| ENSG00000166153 | DEPDC4 | 1.54182486 | 0.624638895 |
| ENSG00000198648 | STK39 | 1.541714407 | 0.62453554 |
| ENSG00000164440 | TXLNB | 1.541308928 | 0.624156054 |
| ENSG00000163126 | ANKRD23 | 1.541165902 | 0.624022172 |
| ENSG00000107223 | EDF1 | 1.541157359 | 0.624014175 |
| ENSG00000076321 | KLHL20 | 1.54089653 | 0.62376999 |
| ENSG00000069974 | RAB27A | 1.540754444 | 0.623636952 |
| ENSG00000255374 | TAS2R43 | 1.540472989 | 0.623373386 |
| ENSG00000185808 | PIGP | 1.540239309 | 0.623154522 |
| ENSG00000103042 | SLC38A7 | 1.540239277 | 0.623154491 |
| ENSG00000173992 | CCS | 1.540021832 | 0.622950803 |
| ENSG00000168216 | LMBRD1 | 1.53992009 | 0.622855488 |
| ENSG00000105894 | PTN | 1.539720918 | 0.622668879 |
| ENSG00000149743 | TRPT1 | 1.539589963 | 0.622546171 |
| ENSG00000153029 | MR1 | 1.539510851 | 0.622472036 |
| ENSG00000075188 | NUP37 | 1.539381881 | 0.622351171 |
| ENSG00000103121 | CMC2 | 1.539163769 | 0.622146744 |
| ENSG00000123297 | TSFM | 1.539079191 | 0.622067466 |
| ENSG00000121900 | TMEM54 | 1.538723459 | 0.621733972 |
| ENSG00000197444 | OGDHL | 1.538685292 | 0.621698187 |
| ENSG00000068394 | GPKOW | 1.53838165 | 0.62141346 |
| ENSG00000075218 | GTSE1 | 1.538379727 | 0.621411656 |
| ENSG00000110318 | CEP126 | 1.538287804 | 0.621325448 |
| ENSG00000177200 | CHD9 | 1.537929661 | 0.620989522 |
| ENSG00000105865 | DUS4L | 1.537823521 | 0.620889951 |
| ENSG00000144712 | CAND2 | 1.537812777 | 0.620879871 |
| ENSG00000159256 | MORC3 | 1.537658242 | 0.620734887 |
| ENSG00000134109 | EDEM1 | 1.537493009 | 0.62057985 |
| ENSG00000181626 | ANKRD62 | 1.537395954 | 0.620488777 |
| ENSG00000111879 | FAM184A | 1.53731064 | 0.620408715 |
| ENSG00000170627 | GTSF1 | 1.537223539 | 0.620326973 |
| ENSG00000256087 | ZNF432 | 1.537132635 | 0.620241657 |
| ENSG00000182759 | MAFA | 1.536880545 | 0.620005035 |
| ENSG00000135736 | CCDC102A | 1.536474949 | 0.619624245 |
| ENSG00000163093 | BBS5 | 1.536446263 | 0.61959731 |
| ENSG00000135002 | RFK | 1.536416994 | 0.619569826 |
| ENSG00000175104 | TRAF6 | 1.53623308 | 0.619397121 |
| ENSG00000139132 | FGD4 | 1.53620955 | 0.619375023 |
| ENSG00000115657 | ABCB6 | 1.536175375 | 0.619342928 |
| ENSG00000154814 | OXNAD1 | 1.535927979 | 0.619110568 |
| ENSG00000214226 | C17orf67 | 1.535890098 | 0.619074986 |
| ENSG00000273331 | TM4SF19-TCTEX1D2 | 1.535297317 | 0.618518067 |
| ENSG00000166347 | CYB5A | 1.535270579 | 0.618492941 |
| ENSG00000133103 | COG6 | 1.535119939 | 0.618351378 |
| ENSG00000118454 | ANKRD13C | 1.53490892 | 0.61815305 |
| ENSG00000127527 | EPS15L1 | 1.534703244 | 0.617959717 |
| ENSG00000163611 | SPICE1 | 1.53464549 | 0.617905425 |
| ENSG00000197535 | MYO5A | 1.534333212 | 0.617611828 |
| ENSG00000197857 | ZNF44 | 1.534116787 | 0.617408315 |
| ENSG00000064995 | TAF11 | 1.534048538 | 0.617344131 |
| ENSG00000186051 | TAL2 | 1.534019915 | 0.617317213 |
| ENSG00000025156 | HSF2 | 1.533982163 | 0.617281708 |
| ENSG00000107021 | TBC1D13 | 1.533785687 | 0.617096912 |
| ENSG00000135720 | DYNC1LI2 | 1.533754153 | 0.61706725 |
| ENSG00000162624 | LHX8 | 1.5332 | 0.616545903 |
| ENSG00000214654 | B3GNT10 | 1.532921855 | 0.616284153 |
| ENSG00000179008 | C14orf39 | 1.532631689 | 0.616011041 |
| ENSG00000068400 | GRIPAP1 | 1.532424017 | 0.615815542 |
| ENSG00000119969 | HELLS | 1.532358762 | 0.615754106 |
| ENSG00000132702 | HAPLN2 | 1.532133293 | 0.615541815 |
| ENSG00000178685 | PARP10 | 1.53203942 | 0.615453419 |
| ENSG00000101003 | GINS1 | 1.531993558 | 0.615410231 |
| ENSG00000125510 | OPRL1 | 1.531613511 | 0.615052292 |
| ENSG00000170949 | ZNF160 | 1.531564423 | 0.615006053 |
| ENSG00000060237 | WNK1 | 1.531207557 | 0.614669855 |
| ENSG00000142687 | KIAA0319L | 1.530817118 | 0.614301939 |
| ENSG00000196923 | PDLIM7 | 1.530598414 | 0.61409581 |
| ENSG00000135298 | ADGRB3 | 1.53056198 | 0.614061468 |
| ENSG00000186395 | KRT10 | 1.530396174 | 0.613905172 |
| ENSG00000007866 | TEAD3 | 1.53038546 | 0.613895072 |
| ENSG00000212747 | RTL8B | 1.530259035 | 0.613775886 |
| ENSG00000189043 | NDUFA4 | 1.530095851 | 0.613622031 |
| ENSG00000198478 | SH3BGRL2 | 1.529921998 | 0.6134581 |
| ENSG00000111490 | TBC1D30 | 1.529804342 | 0.613347148 |
| ENSG00000006756 | ARSD | 1.529696943 | 0.61324586 |
| ENSG00000134899 | ERCC5 | 1.529632603 | 0.613185179 |
| ENSG00000171241 | SHCBP1 | 1.529532069 | 0.613090356 |
| ENSG00000144655 | CSRNP1 | 1.529203026 | 0.61277996 |
| ENSG00000104164 | BLOC1S6 | 1.52901307 | 0.612600739 |
| ENSG00000166333 | ILK | 1.528781955 | 0.612382655 |
| ENSG00000184489 | PTP4A3 | 1.528738786 | 0.612341916 |
| ENSG00000173801 | JUP | 1.528638163 | 0.612246953 |
| ENSG00000161267 | BDH1 | 1.528459894 | 0.612078697 |
| ENSG00000068323 | TFE3 | 1.528435427 | 0.612055602 |
| ENSG00000114125 | RNF7 | 1.527947368 | 0.611594849 |
| ENSG00000106399 | RPA3 | 1.527935897 | 0.611584018 |
| ENSG00000104321 | TRPA1 | 1.527928829 | 0.611577344 |
| ENSG00000197296 | FITM2 | 1.527651626 | 0.611315581 |
| ENSG00000069956 | MAPK6 | 1.527514525 | 0.611186099 |
| ENSG00000262209 | PCDHGB3 | 1.527506021 | 0.611178067 |
| ENSG00000100218 | RSPH14 | 1.527471473 | 0.611145436 |
| ENSG00000147853 | AK3 | 1.527467637 | 0.611141814 |
| ENSG00000109265 | KIAA1211 | 1.527383762 | 0.611062591 |
| ENSG00000163072 | NOSTRIN | 1.527161647 | 0.610852776 |
| ENSG00000144895 | EIF2A | 1.526993133 | 0.610693574 |
| ENSG00000170142 | UBE2E1 | 1.526877674 | 0.610584485 |
| ENSG00000166451 | CENPN | 1.526446997 | 0.610177496 |
| ENSG00000278848 | TP53TG3F | 1.526244994 | 0.609986563 |
| ENSG00000158427 | TMSB15B | 1.526143422 | 0.609890548 |
| ENSG00000240303 | ACAD11 | 1.525546072 | 0.60932575 |
| ENSG00000184678 | HIST2H2BE | 1.524901092 | 0.608715669 |
| ENSG00000119421 | NDUFA8 | 1.524794184 | 0.608614522 |
| ENSG00000167705 | RILP | 1.524328354 | 0.608173706 |
| ENSG00000167971 | CASKIN1 | 1.524076955 | 0.60793575 |
| ENSG00000214530 | STARD10 | 1.523937072 | 0.60780333 |
| ENSG00000049769 | PPP1R3F | 1.523854636 | 0.607725287 |
| ENSG00000125122 | LRRC29 | 1.523724539 | 0.607602114 |
| ENSG00000111727 | HCFC2 | 1.523664601 | 0.607545363 |
| ENSG00000149231 | CCDC82 | 1.522971002 | 0.606888472 |
| ENSG00000138496 | PARP9 | 1.522859496 | 0.60678284 |
| ENSG00000101265 | RASSF2 | 1.52279898 | 0.606725509 |
| ENSG00000169896 | ITGAM | 1.522412648 | 0.606359453 |
| ENSG00000242732 | RTL5 | 1.52230626 | 0.606258632 |
| ENSG00000189227 | C15orf61 | 1.522106106 | 0.606068932 |
| ENSG00000198246 | SLC29A3 | 1.522046676 | 0.606012602 |
| ENSG00000161057 | PSMC2 | 1.521916597 | 0.605889299 |
| ENSG00000136936 | XPA | 1.521733581 | 0.6057158 |
| ENSG00000055211 | GINM1 | 1.521724905 | 0.605707575 |
| ENSG00000129003 | VPS13C | 1.521707508 | 0.60569108 |
| ENSG00000144840 | RABL3 | 1.520938841 | 0.604962141 |
| ENSG00000205810 | KLRC3 | 1.520937294 | 0.604960674 |
| ENSG00000111726 | CMAS | 1.520918547 | 0.604942891 |
| ENSG00000204438 | GPANK1 | 1.520864274 | 0.604891409 |
| ENSG00000164687 | FABP5 | 1.520785411 | 0.604816598 |
| ENSG00000112245 | PTP4A1 | 1.520663851 | 0.604701275 |
| ENSG00000171772 | SYCE1 | 1.520609918 | 0.604650106 |
| ENSG00000142609 | CFAP74 | 1.520325023 | 0.604379784 |
| ENSG00000007264 | MATK | 1.520284483 | 0.604341313 |
| ENSG00000172425 | TTC36 | 1.52006 | 0.604128271 |
| ENSG00000167325 | RRM1 | 1.520041042 | 0.604110278 |
| ENSG00000115073 | ACTR1B | 1.519893597 | 0.603970329 |
| ENSG00000114784 | EIF1B | 1.519684522 | 0.603771859 |
| ENSG00000168495 | POLR3D | 1.519671409 | 0.60375941 |
| ENSG00000146085 | MMUT | 1.519442046 | 0.603541649 |
| ENSG00000090263 | MRPS33 | 1.519433031 | 0.60353309 |
| ENSG00000089876 | DHX32 | 1.519210857 | 0.603322121 |
| ENSG00000136682 | CBWD2 | 1.519180873 | 0.603293646 |
| ENSG00000133313 | CNDP2 | 1.519166856 | 0.603280335 |
| ENSG00000158528 | PPP1R9A | 1.519152872 | 0.603267055 |
| ENSG00000104412 | EMC2 | 1.518772387 | 0.602905674 |
| ENSG00000142459 | EVI5L | 1.518695099 | 0.602832256 |
| ENSG00000109270 | LAMTOR3 | 1.518545457 | 0.602690096 |
| ENSG00000104142 | VPS18 | 1.518536449 | 0.602681538 |
| ENSG00000137413 | TAF8 | 1.517926795 | 0.602102216 |
| ENSG00000119866 | BCL11A | 1.517586647 | 0.60177889 |
| ENSG00000129472 | RAB2B | 1.517319088 | 0.601524512 |
| ENSG00000058063 | ATP11B | 1.517246795 | 0.601455773 |
| ENSG00000100097 | LGALS1 | 1.517149069 | 0.601362846 |
| ENSG00000117408 | IPO13 | 1.517056112 | 0.601274448 |
| ENSG00000159176 | CSRP1 | 1.516953207 | 0.601176583 |
| ENSG00000251287 | ALG1L2 | 1.516932694 | 0.601157075 |
| ENSG00000111790 | FGFR1OP2 | 1.516404733 | 0.600654865 |
| ENSG00000138356 | AOX1 | 1.516335787 | 0.600589268 |
| ENSG00000185436 | IFNLR1 | 1.516287218 | 0.600543058 |
| ENSG00000197461 | PDGFA | 1.51620214 | 0.600462106 |
| ENSG00000148337 | CIZ1 | 1.516198444 | 0.600458589 |
| ENSG00000127990 | SGCE | 1.515994401 | 0.600264425 |
| ENSG00000080986 | NDC80 | 1.515930823 | 0.60020392 |
| ENSG00000185669 | SNAI3 | 1.515782981 | 0.600063214 |
| ENSG00000266412 | NCOA4 | 1.515774724 | 0.600055355 |
| ENSG00000087274 | ADD1 | 1.515560301 | 0.599851255 |
| ENSG00000119397 | CNTRL | 1.515461603 | 0.5997573 |
| ENSG00000152253 | SPC25 | 1.515454793 | 0.599750816 |
| ENSG00000124357 | NAGK | 1.515382229 | 0.599681734 |
| ENSG00000171914 | TLN2 | 1.514890669 | 0.599213677 |
| ENSG00000167693 | NXN | 1.514871693 | 0.599195605 |
| ENSG00000123427 | EEF1AKMT3 | 1.514854221 | 0.599178965 |
| ENSG00000159674 | SPON2 | 1.514804984 | 0.599132074 |
| ENSG00000172020 | GAP43 | 1.51479044 | 0.599118221 |
| ENSG00000078304 | PPP2R5C | 1.514364174 | 0.598712186 |
| ENSG00000106415 | GLCCI1 | 1.514264288 | 0.598617025 |
| ENSG00000102043 | MTMR8 | 1.513898891 | 0.598268855 |
| ENSG00000112984 | KIF20A | 1.513685223 | 0.598065222 |
| ENSG00000170989 | S1PR1 | 1.513673967 | 0.598054494 |
| ENSG00000143412 | ANXA9 | 1.513602078 | 0.597985975 |
| ENSG00000001617 | SEMA3F | 1.513349891 | 0.597745582 |
| ENSG00000164902 | PHAX | 1.512841157 | 0.597260518 |
| ENSG00000101871 | MID1 | 1.512811793 | 0.597232515 |
| ENSG00000166503 | HDGFL3 | 1.512552474 | 0.596985194 |
| ENSG00000179855 | GIPC3 | 1.512228062 | 0.596675732 |
| ENSG00000149150 | SLC43A1 | 1.511904803 | 0.596367304 |
| ENSG00000126790 | L3HYPDH | 1.511898986 | 0.596361753 |
| ENSG00000014919 | COX15 | 1.511896966 | 0.596359825 |
| ENSG00000163945 | UVSSA | 1.511726856 | 0.596197492 |
| ENSG00000173157 | ADAMTS20 | 1.511502695 | 0.595983552 |
| ENSG00000134014 | ELP3 | 1.511497193 | 0.5959783 |
| ENSG00000188215 | DCUN1D3 | 1.511454714 | 0.595937754 |
| ENSG00000168026 | TTC21A | 1.511214881 | 0.595708813 |
| ENSG00000063176 | SPHK2 | 1.511186756 | 0.595681963 |
| ENSG00000066185 | ZMYND12 | 1.511142901 | 0.595640095 |
| ENSG00000178104 | PDE4DIP | 1.510967313 | 0.595472451 |
| ENSG00000188021 | UBQLN2 | 1.510615886 | 0.595136864 |
| ENSG00000152455 | SUV39H2 | 1.510530356 | 0.595055177 |
| ENSG00000136098 | NEK3 | 1.510349568 | 0.594882498 |
| ENSG00000157349 | DDX19B | 1.51034429 | 0.594877456 |
| ENSG00000205189 | ZBTB10 | 1.510312817 | 0.594847393 |
| ENSG00000011243 | AKAP8L | 1.510185458 | 0.59472573 |
| ENSG00000125746 | EML2 | 1.510149031 | 0.594690931 |
| ENSG00000136770 | DNAJC1 | 1.510067981 | 0.594613499 |
| ENSG00000168389 | MFSD2A | 1.509655483 | 0.594219351 |
| ENSG00000130592 | LSP1 | 1.509621742 | 0.594187106 |
| ENSG00000099256 | PRTFDC1 | 1.509414283 | 0.593988832 |
| ENSG00000100196 | KDELR3 | 1.509404638 | 0.593979612 |
| ENSG00000131979 | GCH1 | 1.509389688 | 0.593965323 |
| ENSG00000157978 | LDLRAP1 | 1.509319417 | 0.593898155 |
| ENSG00000120327 | PCDHB14 | 1.508925774 | 0.59352184 |
| ENSG00000079150 | FKBP7 | 1.508919441 | 0.593515784 |
| ENSG00000189377 | CXCL17 | 1.508502819 | 0.593117392 |
| ENSG00000130522 | JUND | 1.508253799 | 0.592879216 |
| ENSG00000075340 | ADD2 | 1.508210622 | 0.592837915 |
| ENSG00000168137 | SETD5 | 1.508107989 | 0.592739737 |
| ENSG00000104671 | DCTN6 | 1.508047101 | 0.592681489 |
| ENSG00000067208 | EVI5 | 1.508010497 | 0.592646471 |
| ENSG00000168243 | GNG4 | 1.507824602 | 0.592468616 |
| ENSG00000143633 | C1orf131 | 1.507793621 | 0.592438973 |
| ENSG00000044524 | EPHA3 | 1.507509838 | 0.592167417 |
| ENSG00000143575 | HAX1 | 1.507373916 | 0.592037333 |
| ENSG00000245680 | ZNF585B | 1.507033258 | 0.591711256 |
| ENSG00000188095 | MESP2 | 1.50652513 | 0.591224739 |
| ENSG00000104361 | NIPAL2 | 1.506515101 | 0.591215134 |
| ENSG00000178860 | MSC | 1.50639609 | 0.59110116 |
| ENSG00000154839 | SKA1 | 1.50621572 | 0.590928408 |
| ENSG00000146233 | CYP39A1 | 1.506091686 | 0.5908096 |
| ENSG00000177565 | TBL1XR1 | 1.505916071 | 0.590641367 |
| ENSG00000170469 | SPATA24 | 1.505897923 | 0.590623981 |
| ENSG00000116254 | CHD5 | 1.505806751 | 0.590536632 |
| ENSG00000183617 | MRPL54 | 1.505779437 | 0.590510463 |
| ENSG00000101115 | SALL4 | 1.505594188 | 0.590332964 |
| ENSG00000110013 | SIAE | 1.505591933 | 0.590330804 |
| ENSG00000184986 | TMEM121 | 1.505548862 | 0.590289531 |
| ENSG00000181409 | AATK | 1.505419069 | 0.590165151 |
| ENSG00000082515 | MRPL22 | 1.505303654 | 0.590054541 |
| ENSG00000170348 | TMED10 | 1.504956162 | 0.589721463 |
| ENSG00000151445 | VIPAS39 | 1.504841618 | 0.589611653 |
| ENSG00000151239 | TWF1 | 1.504639898 | 0.589418252 |
| ENSG00000162510 | MATN1 | 1.504532504 | 0.589315275 |
| ENSG00000185305 | ARL15 | 1.504439329 | 0.589225927 |
| ENSG00000270249 | AC093668.1 | 1.504426953 | 0.589214059 |
| ENSG00000099624 | ATP5F1D | 1.504402169 | 0.589190291 |
| ENSG00000110172 | CHORDC1 | 1.503499009 | 0.588323917 |
| ENSG00000073350 | LLGL2 | 1.503330633 | 0.588162341 |
| ENSG00000136710 | CCDC115 | 1.503275166 | 0.588109111 |
| ENSG00000106245 | BUD31 | 1.50297103 | 0.587817202 |
| ENSG00000187583 | PLEKHN1 | 1.502358896 | 0.587229497 |
| ENSG00000170430 | MGMT | 1.502288462 | 0.587161858 |
| ENSG00000068885 | IFT80 | 1.502113685 | 0.586994005 |
| ENSG00000152767 | FARP1 | 1.501992151 | 0.586877274 |
| ENSG00000169228 | RAB24 | 1.501969961 | 0.586855959 |
| ENSG00000283239 | AC019257.8 | 1.501925956 | 0.586813691 |
| ENSG00000277972 | CISD3 | 1.501760696 | 0.586654939 |
| ENSG00000179603 | GRM8 | 1.501735394 | 0.586630632 |
| ENSG00000143367 | TUFT1 | 1.501646201 | 0.586544943 |
| ENSG00000171428 | NAT1 | 1.501351629 | 0.586261908 |
| ENSG00000150779 | TIMM8B | 1.501304887 | 0.586216991 |
| ENSG00000228474 | OST4 | 1.50122916 | 0.586144218 |
| ENSG00000111052 | LIN7A | 1.501153924 | 0.586071915 |
| ENSG00000126524 | SBDS | 1.501100044 | 0.586020132 |
| ENSG00000204616 | TRIM31 | 1.50098 | 0.585904754 |
| ENSG00000196914 | ARHGEF12 | 1.500948092 | 0.585874084 |
| ENSG00000112394 | SLC16A10 | 1.50070996 | 0.585645177 |
| ENSG00000129991 | TNNI3 | 1.500571294 | 0.585511865 |
| ENSG00000213047 | DENND1B | 1.500377529 | 0.585325562 |
| ENSG00000151689 | INPP1 | 1.500295066 | 0.585246267 |
| ENSG00000196511 | TPK1 | 1.500264576 | 0.585216947 |
| ENSG00000229809 | ZNF688 | 1.500201387 | 0.585156181 |
| ENSG00000109111 | SUPT6H | 1.50017726 | 0.585132979 |
| ESG | symbol | fold | log2 |
| ENSG00000145506 | NKD2 | 0.669721779 | -0.578366211 |
| ENSG00000165275 | TRMT10B | 0.669483948 | -0.578878631 |
| ENSG00000103187 | COTL1 | 0.669318939 | -0.579234258 |
| ENSG00000198663 | C6orf89 | 0.668941242 | -0.5800486 |
| ENSG00000169683 | LRRC45 | 0.668694702 | -0.580580408 |
| ENSG00000034533 | ASTE1 | 0.668525976 | -0.580944476 |
| ENSG00000137478 | FCHSD2 | 0.66843726 | -0.58113594 |
| ENSG00000138698 | RAP1GDS1 | 0.668367373 | -0.581286787 |
| ENSG00000145476 | CYP4V2 | 0.668334287 | -0.581358205 |
| ENSG00000119707 | RBM25 | 0.668232926 | -0.581577025 |
| ENSG00000104907 | TRMT1 | 0.668223085 | -0.581598271 |
| ENSG00000140199 | SLC12A6 | 0.66802738 | -0.58202086 |
| ENSG00000011478 | QPCTL | 0.667948747 | -0.582190689 |
| ENSG00000143110 | C1orf162 | 0.667680523 | -0.582770138 |
| ENSG00000173480 | ZNF417 | 0.667468918 | -0.583227438 |
| ENSG00000185989 | RASA3 | 0.667462096 | -0.583242184 |
| ENSG00000149541 | B3GAT3 | 0.667384483 | -0.583409951 |
| ENSG00000138744 | NAAA | 0.667190274 | -0.583829837 |
| ENSG00000099282 | TSPAN15 | 0.667077046 | -0.584074695 |
| ENSG00000082497 | SERTAD4 | 0.667053505 | -0.584125609 |
| ENSG00000132603 | NIP7 | 0.666916039 | -0.58442295 |
| ENSG00000215915 | ATAD3C | 0.666812191 | -0.584647613 |
| ENSG00000170876 | TMEM43 | 0.666667402 | -0.584960908 |
| ENSG00000065989 | PDE4A | 0.666395205 | -0.585550074 |
| ENSG00000170632 | ARMC10 | 0.666308917 | -0.585736895 |
| ENSG00000185825 | BCAP31 | 0.666163185 | -0.586052467 |
| ENSG00000158089 | GALNT14 | 0.665937142 | -0.586542088 |
| ENSG00000112149 | CD83 | 0.665933157 | -0.58655072 |
| ENSG00000125247 | TMTC4 | 0.665746248 | -0.586955703 |
| ENSG00000143514 | TP53BP2 | 0.665324072 | -0.587870861 |
| ENSG00000179431 | FJX1 | 0.665117905 | -0.588317987 |
| ENSG00000161243 | AC010605.1 | 0.665093402 | -0.588371137 |
| ENSG00000100038 | TOP3B | 0.664963993 | -0.588651872 |
| ENSG00000012232 | EXTL3 | 0.664871323 | -0.588852942 |
| ENSG00000105186 | ANKRD27 | 0.664807029 | -0.588992459 |
| ENSG00000086062 | B4GALT1 | 0.664688362 | -0.58925 |
| ENSG00000164040 | PGRMC2 | 0.664525331 | -0.589603901 |
| ENSG00000134882 | UBAC2 | 0.664235949 | -0.59023229 |
| ENSG00000166171 | DPCD | 0.663870676 | -0.591025867 |
| ENSG00000108591 | DRG2 | 0.663743987 | -0.59130121 |
| ENSG00000159069 | FBXW5 | 0.66362969 | -0.591549663 |
| ENSG00000162494 | LRRC38 | 0.663617125 | -0.591576979 |
| ENSG00000165672 | PRDX3 | 0.663515897 | -0.591797064 |
| ENSG00000126464 | PRR12 | 0.663481228 | -0.591872447 |
| ENSG00000165449 | SLC16A9 | 0.663417494 | -0.59201104 |
| ENSG00000172466 | ZNF24 | 0.663293408 | -0.592280907 |
| ENSG00000080815 | PSEN1 | 0.663185605 | -0.592515402 |
| ENSG00000185800 | DMWD | 0.663144973 | -0.592603796 |
| ENSG00000254673 | AC110275.1 | 0.66289084 | -0.593156776 |
| ENSG00000242689 | CNTF | 0.662828058 | -0.59329342 |
| ENSG00000175582 | RAB6A | 0.662738732 | -0.593487858 |
| ENSG00000171163 | ZNF692 | 0.662204787 | -0.594650654 |
| ENSG00000113578 | FGF1 | 0.662019352 | -0.595054704 |
| ENSG00000166886 | NAB2 | 0.66197882 | -0.595143035 |
| ENSG00000142920 | AZIN2 | 0.661704232 | -0.595741588 |
| ENSG00000137075 | RNF38 | 0.661630419 | -0.595902529 |
| ENSG00000135392 | DNAJC14 | 0.661620476 | -0.59592421 |
| ENSG00000112699 | GMDS | 0.661489757 | -0.596209278 |
| ENSG00000075223 | SEMA3C | 0.661377618 | -0.596453871 |
| ENSG00000173726 | TOMM20 | 0.661279899 | -0.596667047 |
| ENSG00000162129 | CLPB | 0.661229545 | -0.596776907 |
| ENSG00000169684 | CHRNA5 | 0.661088363 | -0.597084976 |
| ENSG00000125901 | MRPS26 | 0.660843061 | -0.597620399 |
| ENSG00000180776 | ZDHHC20 | 0.660813683 | -0.597684535 |
| ENSG00000126500 | FLRT1 | 0.660640635 | -0.598062384 |
| ENSG00000138758 | SEPT11 | 0.660426494 | -0.598530097 |
| ENSG00000183718 | TRIM52 | 0.660280351 | -0.598849381 |
| ENSG00000103647 | CORO2B | 0.660211012 | -0.599000893 |
| ENSG00000180574 | EIF2S3B | 0.66018362 | -0.59906075 |
| ENSG00000166340 | TPP1 | 0.659963101 | -0.59954273 |
| ENSG00000167004 | PDIA3 | 0.65995876 | -0.59955222 |
| ENSG00000148840 | PPRC1 | 0.659745399 | -0.60001871 |
| ENSG00000188725 | SMIM15 | 0.659674676 | -0.600173371 |
| ENSG00000285471 | AC007846.2 | 0.659474646 | -0.600610901 |
| ENSG00000182544 | MFSD5 | 0.659181739 | -0.601251818 |
| ENSG00000105227 | PRX | 0.659077715 | -0.601479505 |
| ENSG00000146834 | MEPCE | 0.659005488 | -0.601637615 |
| ENSG00000142156 | COL6A1 | 0.658939901 | -0.601781204 |
| ENSG00000105245 | NUMBL | 0.658911432 | -0.601843537 |
| ENSG00000198840 | MT-ND3 | 0.658626202 | -0.602468188 |
| ENSG00000110442 | COMMD9 | 0.658580755 | -0.60256774 |
| ENSG00000153558 | FBXL2 | 0.658540899 | -0.602655052 |
| ENSG00000204428 | LY6G5C | 0.658519693 | -0.60270151 |
| ENSG00000171517 | LPAR3 | 0.658446766 | -0.602861288 |
| ENSG00000175322 | ZNF519 | 0.65825852 | -0.603273805 |
| ENSG00000124785 | NRN1 | 0.658021837 | -0.603792634 |
| ENSG00000053438 | NNAT | 0.657839126 | -0.604193278 |
| ENSG00000047932 | GOPC | 0.657818357 | -0.604238826 |
| ENSG00000144749 | LRIG1 | 0.657784992 | -0.604312002 |
| ENSG00000121579 | NAA50 | 0.657672226 | -0.60455935 |
| ENSG00000112237 | CCNC | 0.657652146 | -0.604603399 |
| ENSG00000185245 | GP1BA | 0.65748531 | -0.604969434 |
| ENSG00000173226 | IQCB1 | 0.65730085 | -0.605374244 |
| ENSG00000132386 | SERPINF1 | 0.657283663 | -0.605411969 |
| ENSG00000160007 | ARHGAP35 | 0.657101096 | -0.605812746 |
| ENSG00000213398 | LCAT | 0.656799618 | -0.606474807 |
| ENSG00000257594 | GALNT4 | 0.65673829 | -0.606609524 |
| ENSG00000157500 | APPL1 | 0.65648798 | -0.6071595 |
| ENSG00000162144 | CYB561A3 | 0.656407315 | -0.607336779 |
| ENSG00000144785 | AC073896.1 | 0.656159529 | -0.607881481 |
| ENSG00000259305 | ZHX1-C8orf76 | 0.656113168 | -0.607983419 |
| ENSG00000211455 | STK38L | 0.656004333 | -0.608222751 |
| ENSG00000159873 | CCDC117 | 0.655744951 | -0.608793301 |
| ENSG00000120756 | PLS1 | 0.655672388 | -0.608952955 |
| ENSG00000184445 | KNTC1 | 0.654952454 | -0.610537916 |
| ENSG00000135502 | SLC26A10 | 0.654570135 | -0.611380315 |
| ENSG00000175505 | CLCF1 | 0.654294057 | -0.611988927 |
| ENSG00000184708 | EIF4ENIF1 | 0.654186701 | -0.612225665 |
| ENSG00000164849 | GPR146 | 0.654163801 | -0.612276168 |
| ENSG00000140839 | CLEC18B | 0.653958036 | -0.612730033 |
| ENSG00000147548 | NSD3 | 0.653922564 | -0.61280829 |
| ENSG00000064545 | TMEM161A | 0.65347527 | -0.613795456 |
| ENSG00000228253 | MT-ATP8 | 0.653362447 | -0.614044559 |
| ENSG00000285791 | AC009879.3 | 0.653260595 | -0.614269477 |
| ENSG00000257093 | KIAA1147 | 0.653106038 | -0.61461085 |
| ENSG00000286001 | AC021087.5 | 0.652905936 | -0.615052937 |
| ENSG00000118655 | DCLRE1B | 0.652722812 | -0.615457635 |
| ENSG00000125843 | AP5S1 | 0.652647164 | -0.615624845 |
| ENSG00000026025 | VIM | 0.652330313 | -0.616325424 |
| ENSG00000115275 | MOGS | 0.652224381 | -0.616559722 |
| ENSG00000106244 | PDAP1 | 0.652043627 | -0.616959599 |
| ENSG00000102158 | MAGT1 | 0.651882232 | -0.617316743 |
| ENSG00000164930 | FZD6 | 0.651792116 | -0.617516194 |
| ENSG00000159063 | ALG8 | 0.651570043 | -0.61800782 |
| ENSG00000078269 | SYNJ2 | 0.651433257 | -0.61831072 |
| ENSG00000026036 | RTEL1-TNFRSF6B | 0.651319697 | -0.618562238 |
| ENSG00000146830 | GIGYF1 | 0.651179448 | -0.618872927 |
| ENSG00000163659 | TIPARP | 0.651172055 | -0.618889306 |
| ENSG00000074527 | NTN4 | 0.651153921 | -0.618929485 |
| ENSG00000160796 | NBEAL2 | 0.651119999 | -0.619004644 |
| ENSG00000138119 | MYOF | 0.650954788 | -0.619370749 |
| ENSG00000103160 | HSDL1 | 0.650938872 | -0.619406025 |
| ENSG00000164808 | SPIDR | 0.650880306 | -0.619535832 |
| ENSG00000271092 | TMEM56-RWDD3 | 0.650879491 | -0.61953764 |
| ENSG00000212907 | MT-ND4L | 0.650803248 | -0.619706644 |
| ENSG00000268465 | AC008403.1 | 0.650766701 | -0.619787664 |
| ENSG00000006062 | MAP3K14 | 0.650672254 | -0.619997059 |
| ENSG00000187957 | DNER | 0.650367118 | -0.620673777 |
| ENSG00000156374 | PCGF6 | 0.650017582 | -0.621449353 |
| ENSG00000065600 | TMEM206 | 0.649858876 | -0.621801639 |
| ENSG00000188157 | AGRN | 0.649550204 | -0.622487059 |
| ENSG00000136068 | FLNB | 0.649316363 | -0.623006529 |
| ENSG00000110328 | GALNT18 | 0.649229188 | -0.623200233 |
| ENSG00000241404 | EGFL8 | 0.649067792 | -0.623558926 |
| ENSG00000115159 | GPD2 | 0.649066034 | -0.623562834 |
| ENSG00000130935 | NOL11 | 0.648616674 | -0.624561983 |
| ENSG00000184557 | SOCS3 | 0.648435859 | -0.62496422 |
| ENSG00000108799 | EZH1 | 0.648426525 | -0.624984987 |
| ENSG00000188994 | ZNF292 | 0.648422999 | -0.624992833 |
| ENSG00000198898 | CAPZA2 | 0.648190186 | -0.625510917 |
| ENSG00000168685 | IL7R | 0.648067347 | -0.625784349 |
| ENSG00000259112 | NDUFC2-KCTD14 | 0.647896199 | -0.626165401 |
| ENSG00000104738 | MCM4 | 0.647885368 | -0.626189518 |
| ENSG00000147684 | NDUFB9 | 0.64779361 | -0.626393859 |
| ENSG00000176155 | CCDC57 | 0.647618452 | -0.626784002 |
| ENSG00000155760 | FZD7 | 0.647493556 | -0.627062259 |
| ENSG00000135372 | NAT10 | 0.647373366 | -0.627330083 |
| ENSG00000112599 | GUCA1B | 0.647362518 | -0.627354257 |
| ENSG00000156973 | PDE6D | 0.646803093 | -0.628601517 |
| ENSG00000234616 | JRK | 0.646692394 | -0.628848453 |
| ENSG00000269590 | AC010422.5 | 0.646640637 | -0.62896392 |
| ENSG00000035499 | DEPDC1B | 0.646221969 | -0.629898297 |
| ENSG00000168615 | ADAM9 | 0.645925958 | -0.630559296 |
| ENSG00000106299 | WASL | 0.645850882 | -0.63072699 |
| ENSG00000147454 | SLC25A37 | 0.645510887 | -0.631486667 |
| ENSG00000261052 | SULT1A3 | 0.645472387 | -0.631572715 |
| ENSG00000120093 | HOXB3 | 0.645458331 | -0.631604133 |
| ENSG00000187676 | B3GLCT | 0.645243064 | -0.632085367 |
| ENSG00000196689 | TRPV1 | 0.645095827 | -0.632414611 |
| ENSG00000166526 | ZNF3 | 0.64500128 | -0.632626072 |
| ENSG00000102098 | SCML2 | 0.644867689 | -0.632924909 |
| ENSG00000156466 | GDF6 | 0.644728394 | -0.633236574 |
| ENSG00000186470 | BTN3A2 | 0.644225905 | -0.634361421 |
| ENSG00000198431 | TXNRD1 | 0.644106476 | -0.634628898 |
| ENSG00000177733 | HNRNPA0 | 0.644097892 | -0.634648125 |
| ENSG00000188042 | ARL4C | 0.644094821 | -0.634655003 |
| ENSG00000117226 | GBP3 | 0.643773738 | -0.635374369 |
| ENSG00000006327 | TNFRSF12A | 0.643669114 | -0.635608851 |
| ENSG00000283228 | AC068547.1 | 0.643372975 | -0.636272758 |
| ENSG00000177666 | PNPLA2 | 0.643288712 | -0.636461722 |
| ENSG00000166920 | C15orf48 | 0.642512222 | -0.638204197 |
| ENSG00000140464 | PML | 0.642388777 | -0.638481406 |
| ENSG00000134627 | PIWIL4 | 0.642205798 | -0.638892405 |
| ENSG00000171475 | WIPF2 | 0.64209593 | -0.63913924 |
| ENSG00000132591 | ERAL1 | 0.642066876 | -0.639204523 |
| ENSG00000137309 | HMGA1 | 0.641869024 | -0.639649156 |
| ENSG00000136895 | GARNL3 | 0.641707976 | -0.64001118 |
| ENSG00000140350 | ANP32A | 0.641647803 | -0.640146467 |
| ENSG00000107130 | NCS1 | 0.641451644 | -0.640587583 |
| ENSG00000148153 | INIP | 0.641292927 | -0.6409446 |
| ENSG00000010704 | HFE | 0.640849003 | -0.641943625 |
| ENSG00000275395 | FCGBP | 0.640754685 | -0.642155974 |
| ENSG00000182979 | MTA1 | 0.640722878 | -0.642227591 |
| ENSG00000186086 | NBPF6 | 0.640707179 | -0.64226294 |
| ENSG00000183780 | SLC35F3 | 0.640680447 | -0.642323133 |
| ENSG00000134508 | CABLES1 | 0.640632522 | -0.642431056 |
| ENSG00000143702 | CEP170 | 0.640372025 | -0.64301781 |
| ENSG00000112234 | FBXL4 | 0.640278442 | -0.64322866 |
| ENSG00000241878 | PISD | 0.64027346 | -0.643239886 |
| ENSG00000113456 | RAD1 | 0.639811911 | -0.644280245 |
| ENSG00000172269 | DPAGT1 | 0.639665377 | -0.644610698 |
| ENSG00000152082 | MZT2B | 0.639467273 | -0.64505757 |
| ENSG00000185721 | DRG1 | 0.639319177 | -0.645391725 |
| ENSG00000129625 | REEP5 | 0.639236787 | -0.645577659 |
| ENSG00000023445 | BIRC3 | 0.639137433 | -0.64580191 |
| ENSG00000164010 | ERMAP | 0.638587308 | -0.647044215 |
| ENSG00000139793 | MBNL2 | 0.638463111 | -0.647324828 |
| ENSG00000198938 | MT-CO3 | 0.638262784 | -0.647777565 |
| ENSG00000211456 | SACM1L | 0.638039663 | -0.648281984 |
| ENSG00000185250 | PPIL6 | 0.637999332 | -0.648373181 |
| ENSG00000120063 | GNA13 | 0.637980563 | -0.648415624 |
| ENSG00000136628 | EPRS | 0.637961505 | -0.64845872 |
| ENSG00000051825 | MPHOSPH9 | 0.637849252 | -0.648712595 |
| ENSG00000285053 | TBCE | 0.637519387 | -0.649458879 |
| ENSG00000130844 | ZNF331 | 0.637493678 | -0.64951706 |
| ENSG00000128923 | MINDY2 | 0.637373792 | -0.649788397 |
| ENSG00000129646 | QRICH2 | 0.637197704 | -0.650187027 |
| ENSG00000242612 | DECR2 | 0.636701568 | -0.651310779 |
| ENSG00000271601 | LIX1L | 0.636560207 | -0.651631122 |
| ENSG00000166949 | SMAD3 | 0.63655883 | -0.651634243 |
| ENSG00000141429 | GALNT1 | 0.63653845 | -0.651680433 |
| ENSG00000163882 | POLR2H | 0.636313664 | -0.652189992 |
| ENSG00000283486 | FAM95C | 0.636226703 | -0.652387171 |
| ENSG00000160223 | ICOSLG | 0.636203829 | -0.652439039 |
| ENSG00000116584 | ARHGEF2 | 0.63588222 | -0.653168525 |
| ENSG00000159363 | ATP13A2 | 0.635848514 | -0.653244999 |
| ENSG00000213380 | COG8 | 0.63537049 | -0.65433001 |
| ENSG00000256043 | CTSO | 0.635132896 | -0.6548696 |
| ENSG00000130810 | PPAN | 0.63495314 | -0.655277971 |
| ENSG00000124702 | KLHDC3 | 0.634794112 | -0.655639348 |
| ENSG00000126001 | CEP250 | 0.634488811 | -0.656333372 |
| ENSG00000174579 | MSL2 | 0.634467709 | -0.656381354 |
| ENSG00000196338 | NLGN3 | 0.634433703 | -0.656458681 |
| ENSG00000107738 | VSIR | 0.634431142 | -0.656464506 |
| ENSG00000162923 | WDR26 | 0.634412032 | -0.656507962 |
| ENSG00000284969 | AL049629.2 | 0.634343731 | -0.656663291 |
| ENSG00000196196 | HRCT1 | 0.633876981 | -0.657725216 |
| ENSG00000136783 | NIPSNAP3A | 0.633471072 | -0.658649356 |
| ENSG00000006530 | AGK | 0.633216138 | -0.65923007 |
| ENSG00000162062 | TEDC2 | 0.632917344 | -0.659910992 |
| ENSG00000204149 | AGAP6 | 0.632509991 | -0.660839826 |
| ENSG00000130733 | YIPF2 | 0.63246832 | -0.660934876 |
| ENSG00000187134 | AKR1C1 | 0.632397272 | -0.661096951 |
| ENSG00000185624 | P4HB | 0.632295722 | -0.661328636 |
| ENSG00000146963 | LUC7L2 | 0.632110953 | -0.661750281 |
| ENSG00000148400 | NOTCH1 | 0.63187428 | -0.662290552 |
| ENSG00000102221 | JADE3 | 0.631749213 | -0.662576134 |
| ENSG00000046604 | DSG2 | 0.631609 | -0.662896367 |
| ENSG00000178773 | CPNE7 | 0.631232037 | -0.663757667 |
| ENSG00000107863 | ARHGAP21 | 0.631206106 | -0.663816934 |
| ENSG00000182568 | SATB1 | 0.631177577 | -0.663882141 |
| ENSG00000165678 | GHITM | 0.631111923 | -0.664032216 |
| ENSG00000143013 | LMO4 | 0.631003133 | -0.664280925 |
| ENSG00000198948 | MFAP3L | 0.630972674 | -0.664350568 |
| ENSG00000153551 | CMTM7 | 0.630921477 | -0.664467632 |
| ENSG00000186318 | BACE1 | 0.63087813 | -0.664566756 |
| ENSG00000110057 | UNC93B1 | 0.630488599 | -0.665457812 |
| ENSG00000169714 | CNBP | 0.630475297 | -0.66548825 |
| ENSG00000125257 | ABCC4 | 0.630375319 | -0.665717044 |
| ENSG00000163406 | SLC15A2 | 0.630355274 | -0.66576292 |
| ENSG00000204967 | PCDHA4 | 0.630272045 | -0.66595342 |
| ENSG00000160818 | GPATCH4 | 0.630015562 | -0.666540629 |
| ENSG00000256966 | AL513165.2 | 0.629502409 | -0.667716195 |
| ENSG00000167601 | AXL | 0.629415198 | -0.667916079 |
| ENSG00000162337 | LRP5 | 0.629116111 | -0.668601787 |
| ENSG00000135930 | EIF4E2 | 0.628706707 | -0.669540942 |
| ENSG00000070961 | ATP2B1 | 0.628689683 | -0.669580007 |
| ENSG00000084693 | AGBL5 | 0.628393793 | -0.670259164 |
| ENSG00000138835 | RGS3 | 0.62836466 | -0.670326051 |
| ENSG00000124767 | GLO1 | 0.628304297 | -0.670464648 |
| ENSG00000121775 | TMEM39B | 0.627763573 | -0.671706778 |
| ENSG00000155660 | PDIA4 | 0.627642552 | -0.67198493 |
| ENSG00000100479 | POLE2 | 0.627293816 | -0.672786754 |
| ENSG00000141012 | GALNS | 0.626711362 | -0.674126945 |
| ENSG00000099326 | MZF1 | 0.626661087 | -0.674242685 |
| ENSG00000167615 | LENG8 | 0.626622973 | -0.674330432 |
| ENSG00000241852 | C8orf58 | 0.626621102 | -0.67433474 |
| ENSG00000156171 | DRAM2 | 0.626518283 | -0.674571485 |
| ENSG00000126246 | IGFLR1 | 0.626429699 | -0.674775483 |
| ENSG00000119899 | SLC17A5 | 0.626243212 | -0.675205033 |
| ENSG00000166508 | MCM7 | 0.62618584 | -0.67533721 |
| ENSG00000159128 | IFNGR2 | 0.626008755 | -0.675745261 |
| ENSG00000213199 | ASIC3 | 0.625583038 | -0.676726698 |
| ENSG00000179954 | SSC5D | 0.625441482 | -0.677053187 |
| ENSG00000143815 | LBR | 0.625401086 | -0.67714637 |
| ENSG00000138448 | ITGAV | 0.625194204 | -0.677623692 |
| ENSG00000164828 | SUN1 | 0.62511125 | -0.677815128 |
| ENSG00000198899 | MT-ATP6 | 0.624821589 | -0.678483791 |
| ENSG00000092529 | CAPN3 | 0.62480446 | -0.678523343 |
| ENSG00000165195 | PIGA | 0.624479477 | -0.679273935 |
| ENSG00000143537 | ADAM15 | 0.624381863 | -0.679499465 |
| ENSG00000277726 | AL109811.3 | 0.624132316 | -0.680076182 |
| ENSG00000203943 | SAMD13 | 0.624052299 | -0.680261155 |
| ENSG00000048162 | NOP16 | 0.62391188 | -0.680585814 |
| ENSG00000130713 | EXOSC2 | 0.623903849 | -0.680604384 |
| ENSG00000110274 | CEP164 | 0.623899126 | -0.680615306 |
| ENSG00000105438 | KDELR1 | 0.623880522 | -0.680658328 |
| ENSG00000206530 | CFAP44 | 0.623635487 | -0.681225069 |
| ENSG00000130309 | COLGALT1 | 0.622805882 | -0.683145526 |
| ENSG00000139112 | GABARAPL1 | 0.622656642 | -0.683491272 |
| ENSG00000048828 | FAM120A | 0.622446578 | -0.683978073 |
| ENSG00000259066 | AL110118.2 | 0.622427064 | -0.684023302 |
| ENSG00000104936 | DMPK | 0.622403607 | -0.684077674 |
| ENSG00000243477 | NAA80 | 0.621947756 | -0.685134697 |
| ENSG00000189007 | ADAT2 | 0.62144536 | -0.686300545 |
| ENSG00000170464 | DNAJC18 | 0.621344123 | -0.686535589 |
| ENSG00000082516 | GEMIN5 | 0.621272031 | -0.686702988 |
| ENSG00000080189 | SLC35C2 | 0.621137673 | -0.687015022 |
| ENSG00000138678 | GPAT3 | 0.621052631 | -0.687212561 |
| ENSG00000130270 | ATP8B3 | 0.620902206 | -0.687562038 |
| ENSG00000186416 | NKRF | 0.620629923 | -0.688194837 |
| ENSG00000105058 | FAM32A | 0.620566728 | -0.688341746 |
| ENSG00000204231 | RXRB | 0.620458684 | -0.688592949 |
| ENSG00000174080 | CTSF | 0.620266927 | -0.689038893 |
| ENSG00000127564 | PKMYT1 | 0.619369131 | -0.691128612 |
| ENSG00000130821 | SLC6A8 | 0.619321772 | -0.691238931 |
| ENSG00000101337 | TM9SF4 | 0.619318224 | -0.691247196 |
| ENSG00000115875 | SRSF7 | 0.61906037 | -0.691847989 |
| ENSG00000182963 | GJC1 | 0.619003738 | -0.691979974 |
| ENSG00000215041 | NEURL4 | 0.6188161 | -0.692417362 |
| ENSG00000149357 | LAMTOR1 | 0.618379239 | -0.693436211 |
| ENSG00000112062 | MAPK14 | 0.618358864 | -0.693483747 |
| ENSG00000011405 | PIK3C2A | 0.6183182 | -0.693578624 |
| ENSG00000129596 | CDO1 | 0.618082893 | -0.694127759 |
| ENSG00000103855 | CD276 | 0.617833969 | -0.694708901 |
| ENSG00000116977 | LGALS8 | 0.617805859 | -0.694774543 |
| ENSG00000130226 | DPP6 | 0.61772551 | -0.694962185 |
| ENSG00000122778 | KIAA1549 | 0.617646946 | -0.695145682 |
| ENSG00000136932 | TRMO | 0.617272067 | -0.696021588 |
| ENSG00000204564 | C6orf136 | 0.617117088 | -0.696383851 |
| ENSG00000088035 | ALG6 | 0.617112082 | -0.696395555 |
| ENSG00000133816 | MICAL2 | 0.617004025 | -0.696648194 |
| ENSG00000075618 | FSCN1 | 0.616606385 | -0.697578266 |
| ENSG00000106524 | ANKMY2 | 0.61652199 | -0.697775742 |
| ENSG00000103066 | PLA2G15 | 0.616090538 | -0.698785716 |
| ENSG00000165810 | BTNL9 | 0.61606744 | -0.698839806 |
| ENSG00000140443 | IGF1R | 0.615688929 | -0.699726468 |
| ENSG00000258790 | AL121594.1 | 0.615419889 | -0.700357025 |
| ENSG00000106617 | PRKAG2 | 0.615056877 | -0.701208265 |
| ENSG00000139977 | NAA30 | 0.614874529 | -0.701636049 |
| ENSG00000182551 | ADI1 | 0.614827948 | -0.701745348 |
| ENSG00000072954 | TMEM38A | 0.614423036 | -0.702695789 |
| ENSG00000140391 | TSPAN3 | 0.614392161 | -0.702768286 |
| ENSG00000118898 | PPL | 0.613527853 | -0.704799255 |
| ENSG00000033050 | ABCF2 | 0.613376938 | -0.705154171 |
| ENSG00000120697 | ALG5 | 0.613335445 | -0.705251768 |
| ENSG00000174791 | RIN1 | 0.612691757 | -0.706766653 |
| ENSG00000140382 | HMG20A | 0.612687645 | -0.706776336 |
| ENSG00000198888 | MT-ND1 | 0.612370815 | -0.707522567 |
| ENSG00000005100 | DHX33 | 0.612288645 | -0.707716168 |
| ENSG00000160688 | FLAD1 | 0.612271738 | -0.707756004 |
| ENSG00000130045 | NXNL2 | 0.6121898 | -0.707949087 |
| ENSG00000171867 | PRNP | 0.612062999 | -0.708247938 |
| ENSG00000150625 | GPM6A | 0.61197313 | -0.708459786 |
| ENSG00000163249 | CCNYL1 | 0.611780303 | -0.708914437 |
| ENSG00000135414 | GDF11 | 0.611595341 | -0.709350679 |
| ENSG00000094975 | SUCO | 0.611503609 | -0.709567082 |
| ENSG00000163050 | COQ8A | 0.611275061 | -0.710106386 |
| ENSG00000100600 | LGMN | 0.611147391 | -0.710407736 |
| ENSG00000198156 | NPIPB6 | 0.611139372 | -0.710426667 |
| ENSG00000180287 | PLD5 | 0.610870205 | -0.71106222 |
| ENSG00000179218 | CALR | 0.61086724 | -0.711069223 |
| ENSG00000169247 | SH3TC2 | 0.610846483 | -0.711118245 |
| ENSG00000144021 | CIAO1 | 0.610832179 | -0.711152028 |
| ENSG00000174718 | RESF1 | 0.61065687 | -0.711566142 |
| ENSG00000154889 | MPPE1 | 0.610414545 | -0.712138755 |
| ENSG00000121104 | FAM117A | 0.610378637 | -0.712223625 |
| ENSG00000168490 | PHYHIP | 0.610264278 | -0.712493951 |
| ENSG00000163661 | PTX3 | 0.609732809 | -0.713750917 |
| ENSG00000119927 | GPAM | 0.60908781 | -0.715277865 |
| ENSG00000182253 | SYNM | 0.608866018 | -0.715803299 |
| ENSG00000103335 | PIEZO1 | 0.608694882 | -0.716208859 |
| ENSG00000065150 | IPO5 | 0.607919355 | -0.718048143 |
| ENSG00000172366 | MCRIP2 | 0.607772093 | -0.718397663 |
| ENSG00000162413 | KLHL21 | 0.607442544 | -0.719180139 |
| ENSG00000142227 | EMP3 | 0.607164012 | -0.719841813 |
| ENSG00000215788 | TNFRSF25 | 0.607133001 | -0.719915502 |
| ENSG00000163251 | FZD5 | 0.60703846 | -0.72014017 |
| ENSG00000167772 | ANGPTL4 | 0.606816899 | -0.720666832 |
| ENSG00000241106 | HLA-DOB | 0.606771816 | -0.720774019 |
| ENSG00000164167 | LSM6 | 0.60673904 | -0.720851952 |
| ENSG00000164733 | CTSB | 0.606732572 | -0.720867331 |
| ENSG00000185551 | NR2F2 | 0.605988409 | -0.722637897 |
| ENSG00000168502 | MTCL1 | 0.605458765 | -0.723899386 |
| ENSG00000110104 | CCDC86 | 0.605071694 | -0.724821999 |
| ENSG00000090863 | GLG1 | 0.605020256 | -0.724944651 |
| ENSG00000134333 | LDHA | 0.604974088 | -0.725054745 |
| ENSG00000042753 | AP2S1 | 0.604900855 | -0.725229396 |
| ENSG00000163806 | SPDYA | 0.604769115 | -0.725543631 |
| ENSG00000105429 | MEGF8 | 0.604657575 | -0.725809737 |
| ENSG00000068438 | FTSJ1 | 0.604578686 | -0.725997975 |
| ENSG00000170525 | PFKFB3 | 0.604546038 | -0.726075885 |
| ENSG00000151576 | QTRT2 | 0.604483371 | -0.726225444 |
| ENSG00000100311 | PDGFB | 0.604381407 | -0.726468816 |
| ENSG00000155097 | ATP6V1C1 | 0.60364804 | -0.728220471 |
| ENSG00000157227 | MMP14 | 0.603602003 | -0.728330502 |
| ENSG00000163964 | PIGX | 0.603429541 | -0.728742768 |
| ENSG00000140479 | PCSK6 | 0.603383412 | -0.728853059 |
| ENSG00000101695 | RNF125 | 0.603333239 | -0.728973028 |
| ENSG00000153214 | TMEM87B | 0.602623107 | -0.730672102 |
| ENSG00000090861 | AARS | 0.602389385 | -0.731231747 |
| ENSG00000204366 | ZBTB12 | 0.602004874 | -0.732152927 |
| ENSG00000168994 | PXDC1 | 0.601986834 | -0.73219616 |
| ENSG00000174928 | C3orf33 | 0.601682178 | -0.73292647 |
| ENSG00000165476 | REEP3 | 0.601487342 | -0.733393719 |
| ENSG00000196421 | C20orf204 | 0.601280715 | -0.733889409 |
| ENSG00000198885 | ITPRIPL1 | 0.601125651 | -0.734261512 |
| ENSG00000077312 | SNRPA | 0.601033876 | -0.734481788 |
| ENSG00000126012 | KDM5C | 0.601007409 | -0.73454532 |
| ENSG00000140939 | AC074143.1 | 0.600979378 | -0.734612607 |
| ENSG00000105281 | SLC1A5 | 0.600749456 | -0.735164658 |
| ENSG00000137216 | TMEM63B | 0.600721843 | -0.735230971 |
| ENSG00000109118 | PHF12 | 0.600437051 | -0.735915092 |
| ENSG00000163635 | ATXN7 | 0.600385589 | -0.736038747 |
| ENSG00000114019 | AMOTL2 | 0.600234459 | -0.73640195 |
| ENSG00000144583 | MARCH4 | 0.599751629 | -0.737562924 |
| ENSG00000107798 | LIPA | 0.599096825 | -0.739138907 |
| ENSG00000107882 | SUFU | 0.598955821 | -0.739478501 |
| ENSG00000065361 | ERBB3 | 0.598890467 | -0.739635927 |
| ENSG00000125398 | SOX9 | 0.59872935 | -0.7400241 |
| ENSG00000146476 | ARMT1 | 0.59872407 | -0.740036824 |
| ENSG00000101384 | JAG1 | 0.598416848 | -0.740777302 |
| ENSG00000058085 | LAMC2 | 0.598169597 | -0.74137351 |
| ENSG00000162521 | RBBP4 | 0.597925113 | -0.741963289 |
| ENSG00000258529 | AP001781.2 | 0.597893624 | -0.742039268 |
| ENSG00000142949 | PTPRF | 0.59776562 | -0.74234817 |
| ENSG00000250067 | YJEFN3 | 0.597590023 | -0.742772033 |
| ENSG00000146828 | SLC12A9 | 0.597373067 | -0.743295901 |
| ENSG00000114346 | ECT2 | 0.597363775 | -0.743318341 |
| ENSG00000205923 | CEMP1 | 0.596251368 | -0.746007425 |
| ENSG00000115841 | RMDN2 | 0.596006481 | -0.746600076 |
| ENSG00000142166 | IFNAR1 | 0.595625757 | -0.747521952 |
| ENSG00000174136 | RGMB | 0.595068167 | -0.748873153 |
| ENSG00000066697 | MSANTD3 | 0.594488037 | -0.750280316 |
| ENSG00000164307 | ERAP1 | 0.593848392 | -0.751833434 |
| ENSG00000269693 | AC010422.6 | 0.59355142 | -0.752555077 |
| ENSG00000196937 | FAM3C | 0.593430844 | -0.752848182 |
| ENSG00000214717 | ZBED1 | 0.592586295 | -0.754902834 |
| ENSG00000107779 | BMPR1A | 0.592530873 | -0.755037769 |
| ENSG00000155189 | AGPAT5 | 0.59246652 | -0.755194464 |
| ENSG00000171310 | CHST11 | 0.59243395 | -0.755273776 |
| ENSG00000143612 | C1orf43 | 0.592395898 | -0.755366443 |
| ENSG00000026508 | CD44 | 0.592240362 | -0.755745281 |
| ENSG00000097021 | ACOT7 | 0.592212661 | -0.755812761 |
| ENSG00000135801 | TAF5L | 0.591807582 | -0.756799915 |
| ENSG00000008441 | NFIX | 0.591659856 | -0.757160083 |
| ENSG00000116285 | ERRFI1 | 0.59163363 | -0.757224033 |
| ENSG00000175354 | PTPN2 | 0.59130138 | -0.758034452 |
| ENSG00000131467 | PSME3 | 0.591211654 | -0.758253388 |
| ENSG00000166396 | SERPINB7 | 0.590928248 | -0.758945131 |
| ENSG00000067182 | TNFRSF1A | 0.590793305 | -0.759274619 |
| ENSG00000099341 | PSMD8 | 0.590776326 | -0.75931608 |
| ENSG00000148677 | ANKRD1 | 0.590541793 | -0.759888932 |
| ENSG00000183624 | HMCES | 0.59047965 | -0.760040754 |
| ENSG00000197905 | TEAD4 | 0.59038634 | -0.760268753 |
| ENSG00000187244 | BCAM | 0.590174233 | -0.760787161 |
| ENSG00000162402 | USP24 | 0.589740641 | -0.761847475 |
| ENSG00000107566 | ERLIN1 | 0.58971622 | -0.761907219 |
| ENSG00000099194 | SCD | 0.589484654 | -0.762473838 |
| ENSG00000116954 | RRAGC | 0.589460456 | -0.762533062 |
| ENSG00000147471 | PLPBP | 0.589286932 | -0.762957822 |
| ENSG00000141873 | SLC39A3 | 0.588645635 | -0.764528702 |
| ENSG00000114450 | GNB4 | 0.588011864 | -0.766082832 |
| ENSG00000079337 | RAPGEF3 | 0.587754766 | -0.766713762 |
| ENSG00000070214 | SLC44A1 | 0.587635772 | -0.767005874 |
| ENSG00000100034 | PPM1F | 0.587343012 | -0.767724801 |
| ENSG00000164985 | PSIP1 | 0.586764493 | -0.769146523 |
| ENSG00000146674 | IGFBP3 | 0.586710636 | -0.769278949 |
| ENSG00000122026 | RPL21 | 0.5867039 | -0.769295514 |
| ENSG00000166068 | SPRED1 | 0.586683303 | -0.76934616 |
| ENSG00000256453 | DND1 | 0.586439778 | -0.76994513 |
| ENSG00000151651 | ADAM8 | 0.586374823 | -0.770104934 |
| ENSG00000188315 | C3orf62 | 0.585942586 | -0.771168787 |
| ENSG00000286112 | AL441992.2 | 0.585539066 | -0.772162666 |
| ENSG00000110169 | HPX | 0.585413788 | -0.772471371 |
| ENSG00000110917 | MLEC | 0.585003637 | -0.773482502 |
| ENSG00000143418 | CERS2 | 0.584596497 | -0.774486911 |
| ENSG00000113845 | TIMMDC1 | 0.584339575 | -0.775121094 |
| ENSG00000251201 | TMED7-TICAM2 | 0.584001411 | -0.775956239 |
| ENSG00000035403 | VCL | 0.583930101 | -0.776132411 |
| ENSG00000143811 | PYCR2 | 0.58387688 | -0.776263909 |
| ENSG00000251493 | FOXD1 | 0.583696858 | -0.776708793 |
| ENSG00000196497 | IPO4 | 0.583589819 | -0.776973379 |
| ENSG00000173272 | MZT2A | 0.583511489 | -0.777167033 |
| ENSG00000084234 | APLP2 | 0.583255679 | -0.777799647 |
| ENSG00000173611 | SCAI | 0.58318658 | -0.777970574 |
| ENSG00000131368 | MRPS25 | 0.582589811 | -0.779447625 |
| ENSG00000147889 | CDKN2A | 0.582585524 | -0.779458241 |
| ENSG00000164236 | ANKRD33B | 0.582279759 | -0.780215625 |
| ENSG00000165912 | PACSIN3 | 0.582015672 | -0.780870094 |
| ENSG00000148835 | TAF5 | 0.581719611 | -0.781604153 |
| ENSG00000258484 | SPESP1 | 0.581573066 | -0.781967638 |
| ENSG00000134285 | FKBP11 | 0.581269139 | -0.782721781 |
| ENSG00000101966 | XIAP | 0.581178737 | -0.782946173 |
| ENSG00000068001 | HYAL2 | 0.580629447 | -0.784310354 |
| ENSG00000183726 | TMEM50A | 0.580384103 | -0.784920092 |
| ENSG00000144354 | CDCA7 | 0.580322967 | -0.785072068 |
| ENSG00000128271 | ADORA2A | 0.580251822 | -0.785248946 |
| ENSG00000214827 | MTCP1 | 0.58009205 | -0.785646247 |
| ENSG00000148429 | USP6NL | 0.580034834 | -0.785788551 |
| ENSG00000113328 | CCNG1 | 0.580022498 | -0.785819234 |
| ENSG00000204519 | ZNF551 | 0.579861115 | -0.7862207 |
| ENSG00000119929 | CUTC | 0.579512634 | -0.78708798 |
| ENSG00000205981 | DNAJC19 | 0.578720168 | -0.789062173 |
| ENSG00000170145 | SIK2 | 0.578633361 | -0.789278591 |
| ENSG00000050555 | LAMC3 | 0.578415942 | -0.78982078 |
| ENSG00000173457 | PPP1R14B | 0.578323936 | -0.79005028 |
| ENSG00000198951 | NAGA | 0.578271083 | -0.790182134 |
| ENSG00000137203 | TFAP2A | 0.577952531 | -0.79097709 |
| ENSG00000178809 | TRIM73 | 0.577895284 | -0.791119998 |
| ENSG00000172315 | TP53RK | 0.577046539 | -0.793240418 |
| ENSG00000107679 | PLEKHA1 | 0.576784957 | -0.793894556 |
| ENSG00000174640 | SLCO2A1 | 0.5759359 | -0.796019843 |
| ENSG00000091136 | LAMB1 | 0.575320271 | -0.797562791 |
| ENSG00000130779 | CLIP1 | 0.575076789 | -0.798173486 |
| ENSG00000100084 | HIRA | 0.575025217 | -0.798302869 |
| ENSG00000187514 | PTMA | 0.574572284 | -0.799439693 |
| ENSG00000103319 | EEF2K | 0.574432886 | -0.799789749 |
| ENSG00000102931 | ARL2BP | 0.573754961 | -0.801493372 |
| ENSG00000102805 | CLN5 | 0.573651237 | -0.801754207 |
| ENSG00000026950 | BTN3A1 | 0.573575006 | -0.801945937 |
| ENSG00000165949 | IFI27 | 0.573460247 | -0.802234615 |
| ENSG00000164070 | HSPA4L | 0.572220115 | -0.805357881 |
| ENSG00000184347 | SLIT3 | 0.571654595 | -0.806784389 |
| ENSG00000176597 | B3GNT5 | 0.571446825 | -0.807308839 |
| ENSG00000062282 | DGAT2 | 0.571205158 | -0.807919087 |
| ENSG00000082781 | ITGB5 | 0.570540719 | -0.80959824 |
| ENSG00000183087 | GAS6 | 0.570393872 | -0.809969613 |
| ENSG00000170881 | RNF139 | 0.569717825 | -0.81168055 |
| ENSG00000168906 | MAT2A | 0.569354266 | -0.812601483 |
| ENSG00000125520 | SLC2A4RG | 0.568923513 | -0.813693388 |
| ENSG00000077348 | EXOSC5 | 0.568527386 | -0.814698248 |
| ENSG00000135324 | MRAP2 | 0.566622682 | -0.819539742 |
| ENSG00000120669 | SOHLH2 | 0.566610467 | -0.819570843 |
| ENSG00000171951 | SCG2 | 0.566016597 | -0.821083738 |
| ENSG00000012660 | ELOVL5 | 0.565842883 | -0.821526578 |
| ENSG00000108349 | CASC3 | 0.565592829 | -0.822164266 |
| ENSG00000172380 | GNG12 | 0.565550169 | -0.822273086 |
| ENSG00000198763 | MT-ND2 | 0.565362276 | -0.822752473 |
| ENSG00000163762 | TM4SF18 | 0.564940184 | -0.823829972 |
| ENSG00000253250 | C8orf88 | 0.564858564 | -0.824038422 |
| ENSG00000173011 | TADA2B | 0.563202674 | -0.828273911 |
| ENSG00000143554 | SLC27A3 | 0.562869222 | -0.829128331 |
| ENSG00000272916 | AC022400.7 | 0.562467443 | -0.830158503 |
| ENSG00000204839 | MROH6 | 0.56209926 | -0.83110318 |
| ENSG00000180198 | RCC1 | 0.561766044 | -0.831958671 |
| ENSG00000198727 | MT-CYB | 0.561724615 | -0.832065072 |
| ENSG00000275832 | ARHGAP23 | 0.561679609 | -0.832180666 |
| ENSG00000132471 | WBP2 | 0.561220619 | -0.833360082 |
| ENSG00000198211 | AC092143.1 | 0.560481329 | -0.835261781 |
| ENSG00000243232 | PCDHAC2 | 0.559394938 | -0.838060897 |
| ENSG00000139908 | TSSK4 | 0.558589655 | -0.840139239 |
| ENSG00000197321 | SVIL | 0.557757124 | -0.842291059 |
| ENSG00000173852 | DPY19L1 | 0.557561636 | -0.842796797 |
| ENSG00000018408 | WWTR1 | 0.556955922 | -0.84436494 |
| ENSG00000080839 | RBL1 | 0.556927383 | -0.844438866 |
| ENSG00000015475 | BID | 0.556572846 | -0.845357571 |
| ENSG00000273154 | AL121845.3 | 0.556175833 | -0.846387037 |
| ENSG00000138074 | SLC5A6 | 0.55615094 | -0.846451611 |
| ENSG00000111144 | LTA4H | 0.556118449 | -0.846535896 |
| ENSG00000188483 | IER5L | 0.555738959 | -0.847520715 |
| ENSG00000109861 | CTSC | 0.554928634 | -0.849625849 |
| ENSG00000137692 | DCUN1D5 | 0.554794031 | -0.849975828 |
| ENSG00000083444 | PLOD1 | 0.554626236 | -0.850412232 |
| ENSG00000243646 | IL10RB | 0.554173383 | -0.851590676 |
| ENSG00000171298 | GAA | 0.553897396 | -0.85230934 |
| ENSG00000077782 | FGFR1 | 0.553849936 | -0.852432959 |
| ENSG00000122863 | CHST3 | 0.553004081 | -0.854637967 |
| ENSG00000259529 | AL136295.5 | 0.552850243 | -0.855039361 |
| ENSG00000067955 | CBFB | 0.551801955 | -0.857777527 |
| ENSG00000139675 | HNRNPA1L2 | 0.551381571 | -0.858877047 |
| ENSG00000224420 | ADM5 | 0.551373777 | -0.85889744 |
| ENSG00000168000 | BSCL2 | 0.550708944 | -0.860638055 |
| ENSG00000167588 | GPD1 | 0.549925911 | -0.862690832 |
| ENSG00000198791 | CNOT7 | 0.549912035 | -0.862727235 |
| ENSG00000104814 | MAP4K1 | 0.549712964 | -0.863249592 |
| ENSG00000100796 | PPP4R3A | 0.54943867 | -0.863969644 |
| ENSG00000161395 | PGAP3 | 0.549406168 | -0.864054988 |
| ENSG00000178397 | FAM220A | 0.549389773 | -0.864098039 |
| ENSG00000100350 | FOXRED2 | 0.549242266 | -0.864485445 |
| ENSG00000104899 | AMH | 0.548873211 | -0.865455168 |
| ENSG00000244038 | DDOST | 0.548105795 | -0.867473706 |
| ENSG00000169136 | ATF5 | 0.548074822 | -0.867555234 |
| ENSG00000109685 | NSD2 | 0.547414007 | -0.869295746 |
| ENSG00000116406 | EDEM3 | 0.547395009 | -0.869345813 |
| ENSG00000166012 | TAF1D | 0.547173985 | -0.869928455 |
| ENSG00000142751 | GPN2 | 0.547149822 | -0.869992165 |
| ENSG00000285269 | AL160269.1 | 0.546955094 | -0.870505705 |
| ENSG00000113719 | ERGIC1 | 0.5459018 | -0.87328664 |
| ENSG00000175893 | ZDHHC21 | 0.545858144 | -0.873402017 |
| ENSG00000154102 | C16orf74 | 0.545854609 | -0.873411362 |
| ENSG00000110108 | TMEM109 | 0.545686603 | -0.87385547 |
| ENSG00000142627 | EPHA2 | 0.545456254 | -0.874464598 |
| ENSG00000213999 | MEF2B | 0.544552134 | -0.876857918 |
| ENSG00000165914 | TTC7B | 0.543552317 | -0.879509194 |
| ENSG00000122515 | ZMIZ2 | 0.543291805 | -0.88020081 |
| ENSG00000072310 | SREBF1 | 0.542473716 | -0.882374858 |
| ENSG00000105971 | CAV2 | 0.542000636 | -0.88363355 |
| ENSG00000148090 | AUH | 0.54182748 | -0.884094529 |
| ENSG00000111057 | KRT18 | 0.541715114 | -0.884393752 |
| ENSG00000063177 | RPL18 | 0.540563312 | -0.887464495 |
| ENSG00000122642 | FKBP9 | 0.539270648 | -0.890918582 |
| ENSG00000064607 | SUGP2 | 0.539168739 | -0.891191245 |
| ENSG00000105479 | CCDC114 | 0.539055546 | -0.891494155 |
| ENSG00000181090 | EHMT1 | 0.538456118 | -0.89309932 |
| ENSG00000137700 | SLC37A4 | 0.538318093 | -0.893469179 |
| ENSG00000164181 | ELOVL7 | 0.537631436 | -0.895310598 |
| ENSG00000121716 | PILRB | 0.537196079 | -0.896479321 |
| ENSG00000173809 | AC008736.1 | 0.53706464 | -0.896832357 |
| ENSG00000163938 | GNL3 | 0.536976854 | -0.897068193 |
| ENSG00000273155 | AC092587.1 | 0.536769788 | -0.897624622 |
| ENSG00000149218 | ENDOD1 | 0.536685399 | -0.897851455 |
| ENSG00000163002 | NUP35 | 0.536542335 | -0.898236087 |
| ENSG00000129038 | LOXL1 | 0.536423798 | -0.898554852 |
| ENSG00000114554 | PLXNA1 | 0.536363124 | -0.898718042 |
| ENSG00000137496 | IL18BP | 0.536174588 | -0.89922525 |
| ENSG00000197594 | ENPP1 | 0.536146108 | -0.899301883 |
| ENSG00000173264 | GPR137 | 0.536056443 | -0.89954318 |
| ENSG00000131188 | PRR7 | 0.535981069 | -0.89974605 |
| ENSG00000078124 | ACER3 | 0.535730818 | -0.900419806 |
| ENSG00000135437 | RDH5 | 0.534655764 | -0.903317779 |
| ENSG00000109084 | TMEM97 | 0.534437127 | -0.90390786 |
| ENSG00000172830 | SSH3 | 0.534188261 | -0.904579822 |
| ENSG00000124145 | SDC4 | 0.534161342 | -0.904652526 |
| ENSG00000136444 | RSAD1 | 0.534122573 | -0.90475724 |
| ENSG00000143543 | JTB | 0.53368655 | -0.905935441 |
| ENSG00000179403 | VWA1 | 0.532775831 | -0.908399458 |
| ENSG00000103194 | USP10 | 0.532746285 | -0.908479468 |
| ENSG00000123240 | OPTN | 0.532723603 | -0.908540893 |
| ENSG00000087586 | AURKA | 0.532374402 | -0.90948689 |
| ENSG00000163794 | UCN | 0.531429699 | -0.912049238 |
| ENSG00000242028 | HYPK | 0.530546021 | -0.914450195 |
| ENSG00000185499 | MUC1 | 0.530282844 | -0.91516602 |
| ENSG00000126010 | GRPR | 0.529950461 | -0.916070591 |
| ENSG00000117480 | FAAH | 0.529677929 | -0.9168127 |
| ENSG00000261587 | TMEM249 | 0.52793152 | -0.921577292 |
| ENSG00000160131 | VMA21 | 0.527928809 | -0.9215847 |
| ENSG00000225828 | FAM229A | 0.527824773 | -0.92186903 |
| ENSG00000112852 | PCDHB2 | 0.527718683 | -0.922159035 |
| ENSG00000285238 | AC006064.6 | 0.527301873 | -0.923298975 |
| ENSG00000162377 | COA7 | 0.526880575 | -0.924452102 |
| ENSG00000084090 | STARD7 | 0.525045022 | -0.929486957 |
| ENSG00000277117 | FP565260.3 | 0.524122694 | -0.932023517 |
| ENSG00000262484 | CCER2 | 0.523528585 | -0.933659782 |
| ENSG00000109063 | MYH3 | 0.523467675 | -0.933827643 |
| ENSG00000178188 | SH2B1 | 0.523174113 | -0.934636938 |
| ENSG00000069345 | DNAJA2 | 0.522875922 | -0.935459457 |
| ENSG00000145439 | CBR4 | 0.522593922 | -0.936237749 |
| ENSG00000186017 | ZNF566 | 0.521328538 | -0.939735259 |
| ENSG00000148660 | CAMK2G | 0.521263245 | -0.939915959 |
| ENSG00000092295 | TGM1 | 0.520155959 | -0.94298384 |
| ENSG00000123091 | RNF11 | 0.518509327 | -0.947558154 |
| ENSG00000117407 | ARTN | 0.518272147 | -0.948218232 |
| ENSG00000254726 | MEX3A | 0.517910277 | -0.949225907 |
| ENSG00000101187 | SLCO4A1 | 0.517561198 | -0.950198635 |
| ENSG00000116649 | SRM | 0.517103958 | -0.951473746 |
| ENSG00000204305 | AGER | 0.516491061 | -0.953184714 |
| ENSG00000134108 | ARL8B | 0.515639904 | -0.95556418 |
| ENSG00000134013 | LOXL2 | 0.514665444 | -0.958293176 |
| ENSG00000157600 | TMEM164 | 0.512740179 | -0.963700142 |
| ENSG00000112078 | KCTD20 | 0.512736829 | -0.963709566 |
| ENSG00000138162 | TACC2 | 0.511914793 | -0.966024397 |
| ENSG00000166272 | WBP1L | 0.511053663 | -0.968453306 |
| ENSG00000130997 | POLN | 0.510068213 | -0.971237899 |
| ENSG00000172840 | PDP2 | 0.509947251 | -0.971580073 |
| ENSG00000177917 | ARL6IP6 | 0.509931271 | -0.971625283 |
| ENSG00000180340 | FZD2 | 0.509893809 | -0.971731272 |
| ENSG00000027001 | MIPEP | 0.508459014 | -0.975796609 |
| ENSG00000125630 | POLR1B | 0.508259585 | -0.976362577 |
| ENSG00000189143 | CLDN4 | 0.507736152 | -0.977849109 |
| ENSG00000272325 | NUDT3 | 0.50674778 | -0.980660231 |
| ENSG00000213901 | SLC23A3 | 0.506206334 | -0.982202536 |
| ENSG00000125347 | IRF1 | 0.505476343 | -0.984284522 |
| ENSG00000267281 | ATF7-NPFF | 0.504975501 | -0.985714698 |
| ENSG00000094880 | CDC23 | 0.504956111 | -0.985770095 |
| ENSG00000204525 | HLA-C | 0.503499107 | -0.989938876 |
| ENSG00000162775 | RBM15 | 0.50173749 | -0.994995354 |
| ENSG00000161671 | EMC10 | 0.501533076 | -0.995583247 |
| ENSG00000090581 | GNPTG | 0.501381316 | -0.99601986 |
| ENSG00000008283 | CYB561 | 0.501293171 | -0.996273515 |
| ENSG00000132470 | ITGB4 | 0.501277754 | -0.996317883 |
| ENSG00000175592 | FOSL1 | 0.501041423 | -0.996998214 |
| ENSG00000185340 | GAS2L1 | 0.501030608 | -0.997029354 |
| ENSG00000142657 | PGD | 0.500853747 | -0.997538707 |
| ENSG00000170296 | GABARAP | 0.500727496 | -0.997902416 |
| ENSG00000253846 | PCDHGA10 | 0.500404278 | -0.998833972 |
| ENSG00000082996 | RNF13 | 0.499991504 | -1.000024515 |
| ENSG00000198959 | TGM2 | 0.49929428 | -1.002037716 |
| ENSG00000102100 | SLC35A2 | 0.499283316 | -1.002069395 |
| ENSG00000164251 | F2RL1 | 0.498744058 | -1.003628442 |
| ENSG00000145014 | TMEM44 | 0.497212078 | -1.008066754 |
| ENSG00000183864 | TOB2 | 0.496184056 | -1.011052718 |
| ENSG00000171150 | SOCS5 | 0.49608058 | -1.011353612 |
| ENSG00000181222 | POLR2A | 0.495695405 | -1.01247421 |
| ENSG00000100304 | TTLL12 | 0.495267228 | -1.013720934 |
| ENSG00000254806 | SYS1-DBNDD2 | 0.494760001 | -1.015199224 |
| ENSG00000145555 | MYO10 | 0.494109266 | -1.017097983 |
| ENSG00000189403 | HMGB1 | 0.493646794 | -1.018448937 |
| ENSG00000184164 | CRELD2 | 0.493515309 | -1.018833257 |
| ENSG00000110025 | SNX15 | 0.492526562 | -1.021726562 |
| ENSG00000125912 | NCLN | 0.490463436 | -1.027782508 |
| ENSG00000171490 | RSL1D1 | 0.489549038 | -1.030474713 |
| ENSG00000169550 | MUC15 | 0.489272821 | -1.031288951 |
| ENSG00000145623 | OSMR | 0.489163563 | -1.031611152 |
| ENSG00000070614 | NDST1 | 0.488862276 | -1.032500014 |
| ENSG00000124783 | SSR1 | 0.487954745 | -1.035180742 |
| ENSG00000119681 | LTBP2 | 0.487928524 | -1.035258271 |
| ENSG00000244165 | P2RY11 | 0.48783714 | -1.035528496 |
| ENSG00000164414 | SLC35A1 | 0.487470901 | -1.036611993 |
| ENSG00000125871 | MGME1 | 0.486751507 | -1.03874265 |
| ENSG00000111261 | MANSC1 | 0.486542533 | -1.039362167 |
| ENSG00000105887 | MTPN | 0.486464305 | -1.039594144 |
| ENSG00000082641 | NFE2L1 | 0.486355477 | -1.03991693 |
| ENSG00000183691 | NOG | 0.485503426 | -1.042446619 |
| ENSG00000167524 | RSKR | 0.482667788 | -1.050897548 |
| ENSG00000165526 | RPUSD4 | 0.481610432 | -1.054061453 |
| ENSG00000240053 | LY6G5B | 0.481436269 | -1.054583263 |
| ENSG00000100060 | MFNG | 0.481375667 | -1.054764878 |
| ENSG00000117298 | ECE1 | 0.480726154 | -1.056712799 |
| ENSG00000128595 | CALU | 0.480454725 | -1.057527609 |
| ENSG00000126890 | CTAG2 | 0.479823425 | -1.059424503 |
| ENSG00000152465 | NMT2 | 0.47975495 | -1.059630404 |
| ENSG00000148926 | ADM | 0.479668051 | -1.059891746 |
| ENSG00000101346 | POFUT1 | 0.479218344 | -1.06124496 |
| ENSG00000171823 | FBXL14 | 0.478914367 | -1.062160379 |
| ENSG00000243710 | CFAP57 | 0.478690872 | -1.062833798 |
| ENSG00000182004 | SNRPE | 0.47829413 | -1.06403001 |
| ENSG00000154127 | UBASH3B | 0.478238727 | -1.064197132 |
| ENSG00000150593 | PDCD4 | 0.47769713 | -1.065831886 |
| ENSG00000050344 | NFE2L3 | 0.475624318 | -1.072105614 |
| ENSG00000102054 | RBBP7 | 0.475612414 | -1.072141723 |
| ENSG00000108639 | SYNGR2 | 0.475607236 | -1.07215743 |
| ENSG00000145685 | LHFPL2 | 0.474779964 | -1.074669042 |
| ENSG00000138061 | CYP1B1 | 0.471902651 | -1.083438819 |
| ENSG00000133657 | ATP13A3 | 0.471162418 | -1.085703628 |
| ENSG00000148154 | UGCG | 0.471037107 | -1.086087378 |
| ENSG00000068650 | ATP11A | 0.470682706 | -1.087173248 |
| ENSG00000054983 | GALC | 0.470305459 | -1.088330018 |
| ENSG00000163520 | FBLN2 | 0.469203843 | -1.091713265 |
| ENSG00000104894 | CD37 | 0.468521272 | -1.093813542 |
| ENSG00000123600 | METTL8 | 0.468128584 | -1.095023236 |
| ENSG00000072274 | TFRC | 0.467886331 | -1.095770012 |
| ENSG00000134531 | EMP1 | 0.467553172 | -1.096797652 |
| ENSG00000257365 | FNTB | 0.466497138 | -1.100059864 |
| ENSG00000169189 | NSMCE1 | 0.466131426 | -1.101191313 |
| ENSG00000164574 | GALNT10 | 0.465688527 | -1.102562754 |
| ENSG00000155959 | VBP1 | 0.465164675 | -1.104186553 |
| ENSG00000117054 | ACADM | 0.464806675 | -1.105297307 |
| ENSG00000128342 | LIF | 0.462752585 | -1.111687046 |
| ENSG00000160208 | RRP1B | 0.460865021 | -1.117583822 |
| ENSG00000118785 | SPP1 | 0.459976532 | -1.120367839 |
| ENSG00000157540 | DYRK1A | 0.459972961 | -1.120379037 |
| ENSG00000058804 | NDC1 | 0.459419896 | -1.122114757 |
| ENSG00000182934 | SRPRA | 0.459373074 | -1.122261799 |
| ENSG00000100522 | GNPNAT1 | 0.459111262 | -1.123084274 |
| ENSG00000085514 | PILRA | 0.458800402 | -1.12406144 |
| ENSG00000188818 | ZDHHC11 | 0.457474751 | -1.128235976 |
| ENSG00000106366 | SERPINE1 | 0.45627068 | -1.132038145 |
| ENSG00000003056 | M6PR | 0.455626867 | -1.134075275 |
| ENSG00000144231 | POLR2D | 0.455552581 | -1.134310513 |
| ENSG00000059769 | DNAJC25 | 0.454928349 | -1.136288756 |
| ENSG00000133818 | RRAS2 | 0.454403137 | -1.137955299 |
| ENSG00000187268 | FAM9C | 0.454357516 | -1.138100151 |
| ENSG00000117143 | UAP1 | 0.451912434 | -1.145884842 |
| ENSG00000065923 | SLC9A7 | 0.451092051 | -1.148506232 |
| ENSG00000168610 | STAT3 | 0.451031844 | -1.1486988 |
| ENSG00000138829 | FBN2 | 0.450734386 | -1.14965058 |
| ENSG00000213648 | SULT1A4 | 0.449953637 | -1.152151741 |
| ENSG00000065548 | ZC3H15 | 0.449630469 | -1.153188291 |
| ENSG00000197757 | HOXC6 | 0.449123203 | -1.154816837 |
| ENSG00000197467 | COL13A1 | 0.448272447 | -1.157552266 |
| ENSG00000105376 | ICAM5 | 0.4480989 | -1.15811091 |
| ENSG00000136933 | RABEPK | 0.447545802 | -1.159892759 |
| ENSG00000178445 | GLDC | 0.447441335 | -1.160229556 |
| ENSG00000118515 | SGK1 | 0.446825499 | -1.162216576 |
| ENSG00000177192 | PUS1 | 0.446697871 | -1.162628717 |
| ENSG00000132640 | BTBD3 | 0.445356629 | -1.166967027 |
| ENSG00000074582 | BCS1L | 0.444623869 | -1.169342697 |
| ENSG00000115541 | HSPE1 | 0.443711454 | -1.1723063 |
| ENSG00000137404 | NRM | 0.442100478 | -1.177553801 |
| ENSG00000144677 | CTDSPL | 0.440802214 | -1.181796626 |
| ENSG00000129474 | AJUBA | 0.440730717 | -1.182030644 |
| ENSG00000095383 | TBC1D2 | 0.440345967 | -1.183290641 |
| ENSG00000185567 | AHNAK2 | 0.438901382 | -1.188031283 |
| ENSG00000167701 | GPT | 0.438121915 | -1.190595716 |
| ENSG00000261796 | ISY1-RAB43 | 0.43783278 | -1.191548123 |
| ENSG00000084073 | ZMPSTE24 | 0.435770154 | -1.198360706 |
| ENSG00000121774 | KHDRBS1 | 0.435160842 | -1.200379354 |
| ENSG00000131446 | MGAT1 | 0.435030824 | -1.200810468 |
| ENSG00000005884 | ITGA3 | 0.43481256 | -1.20153448 |
| ENSG00000115232 | ITGA4 | 0.433909163 | -1.204535042 |
| ENSG00000185033 | SEMA4B | 0.431241596 | -1.213431754 |
| ENSG00000182871 | COL18A1 | 0.430867632 | -1.214683372 |
| ENSG00000179456 | ZBTB18 | 0.428544153 | -1.222484242 |
| ENSG00000079819 | EPB41L2 | 0.427967524 | -1.224426772 |
| ENSG00000187193 | MT1X | 0.426084244 | -1.23078939 |
| ENSG00000049449 | RCN1 | 0.425732912 | -1.23197947 |
| ENSG00000141540 | TTYH2 | 0.424774953 | -1.235229395 |
| ENSG00000114812 | VIPR1 | 0.424417352 | -1.236444452 |
| ENSG00000143164 | DCAF6 | 0.423288873 | -1.240285529 |
| ENSG00000198162 | MAN1A2 | 0.423072549 | -1.241023016 |
| ENSG00000125454 | SLC25A19 | 0.422719456 | -1.242227581 |
| ENSG00000067167 | TRAM1 | 0.422609513 | -1.242602852 |
| ENSG00000145860 | RNF145 | 0.421906344 | -1.245005313 |
| ENSG00000182667 | NTM | 0.421903341 | -1.245015584 |
| ENSG00000268083 | AC008982.1 | 0.419487194 | -1.253301327 |
| ENSG00000107833 | NPM3 | 0.419190447 | -1.254322254 |
| ENSG00000104660 | LEPROTL1 | 0.418678336 | -1.256085825 |
| ENSG00000101188 | NTSR1 | 0.416378348 | -1.264033047 |
| ENSG00000170779 | CDCA4 | 0.41632306 | -1.264224623 |
| ENSG00000136048 | DRAM1 | 0.415087216 | -1.268513597 |
| ENSG00000243335 | KCTD7 | 0.41323457 | -1.274967145 |
| ENSG00000167774 | AC010323.1 | 0.412689653 | -1.276870829 |
| ENSG00000244462 | RBM12 | 0.410671934 | -1.28394174 |
| ENSG00000198805 | PNP | 0.410047799 | -1.286136002 |
| ENSG00000035862 | TIMP2 | 0.409286582 | -1.288816723 |
| ENSG00000028839 | TBPL1 | 0.409107444 | -1.289448305 |
| ENSG00000156471 | PTDSS1 | 0.407533798 | -1.295008385 |
| ENSG00000213064 | SFT2D2 | 0.407448267 | -1.2953112 |
| ENSG00000011422 | PLAUR | 0.407369999 | -1.295588359 |
| ENSG00000139291 | TMEM19 | 0.406954453 | -1.297060759 |
| ENSG00000150764 | DIXDC1 | 0.4062114 | -1.299697366 |
| ENSG00000179104 | TMTC2 | 0.405882298 | -1.300866675 |
| ENSG00000249624 | AP000295.1 | 0.405101689 | -1.303643996 |
| ENSG00000173950 | XXYLT1 | 0.404716 | -1.30501821 |
| ENSG00000073605 | GSDMB | 0.404673218 | -1.305170724 |
| ENSG00000090857 | PDPR | 0.402888659 | -1.311546901 |
| ENSG00000026103 | FAS | 0.401992281 | -1.314760297 |
| ENSG00000150867 | PIP4K2A | 0.401258122 | -1.3173975 |
| ENSG00000180817 | PPA1 | 0.400876766 | -1.318769291 |
| ENSG00000134294 | SLC38A2 | 0.398563491 | -1.32711853 |
| ENSG00000149591 | TAGLN | 0.397708786 | -1.330215659 |
| ENSG00000162972 | MAIP1 | 0.397154404 | -1.332228094 |
| ENSG00000113070 | HBEGF | 0.39623723 | -1.335563653 |
| ENSG00000151929 | BAG3 | 0.395612143 | -1.337841384 |
| ENSG00000164300 | SERINC5 | 0.395309864 | -1.338944141 |
| ENSG00000103342 | GSPT1 | 0.393780722 | -1.344535612 |
| ENSG00000203896 | LIME1 | 0.393588916 | -1.345238503 |
| ENSG00000105767 | CADM4 | 0.390858287 | -1.355282468 |
| ENSG00000103044 | HAS3 | 0.390063523 | -1.358219004 |
| ENSG00000140526 | ABHD2 | 0.387515427 | -1.367674349 |
| ENSG00000124942 | AHNAK | 0.385285773 | -1.375999182 |
| ENSG00000091409 | ITGA6 | 0.384701486 | -1.378188694 |
| ENSG00000181104 | F2R | 0.384314992 | -1.379638839 |
| ENSG00000258947 | TUBB3 | 0.383517227 | -1.382636714 |
| ENSG00000213699 | SLC35F6 | 0.383210599 | -1.38379063 |
| ENSG00000113621 | TXNDC15 | 0.381702938 | -1.389477804 |
| ENSG00000058673 | ZC3H11A | 0.381007332 | -1.392109333 |
| ENSG00000135069 | PSAT1 | 0.380507111 | -1.39400468 |
| ENSG00000111186 | WNT5B | 0.379552482 | -1.397628709 |
| ENSG00000136197 | C7orf25 | 0.379236135 | -1.398831658 |
| ENSG00000048140 | TSPAN17 | 0.379173223 | -1.39907101 |
| ENSG00000142871 | CCN1 | 0.378934568 | -1.399979341 |
| ENSG00000130312 | MRPL34 | 0.378411475 | -1.401972258 |
| ENSG00000249992 | TMEM158 | 0.374900681 | -1.415419648 |
| ENSG00000188223 | AD000671.1 | 0.374781971 | -1.415876543 |
| ENSG00000116237 | ICMT | 0.374576911 | -1.41666612 |
| ENSG00000117862 | TXNDC12 | 0.374498281 | -1.416968997 |
| ENSG00000165490 | DDIAS | 0.373002731 | -1.422741903 |
| ENSG00000198363 | ASPH | 0.372879775 | -1.423217549 |
| ENSG00000165507 | DEPP1 | 0.369937886 | -1.434645036 |
| ENSG00000076067 | RBMS2 | 0.368052106 | -1.442018067 |
| ENSG00000090339 | ICAM1 | 0.3665088 | -1.448080255 |
| ENSG00000214300 | SPDYE3 | 0.363972717 | -1.458097785 |
| ENSG00000171603 | CLSTN1 | 0.36383146 | -1.4586578 |
| ENSG00000254245 | PCDHGA3 | 0.363698029 | -1.459186988 |
| ENSG00000135407 | AVIL | 0.361673157 | -1.467241568 |
| ENSG00000132670 | PTPRA | 0.359410952 | -1.476293725 |
| ENSG00000164362 | TERT | 0.358381594 | -1.480431551 |
| ENSG00000118523 | CCN2 | 0.355658842 | -1.491434065 |
| ENSG00000072501 | SMC1A | 0.35196507 | -1.506495838 |
| ENSG00000138031 | ADCY3 | 0.351303926 | -1.509208397 |
| ENSG00000166889 | PATL1 | 0.348613575 | -1.520299348 |
| ENSG00000256100 | AP000721.1 | 0.348510744 | -1.520724964 |
| ENSG00000285130 | AL358113.1 | 0.347161165 | -1.526322526 |
| ENSG00000068489 | PRR11 | 0.346382334 | -1.529562743 |
| ENSG00000142798 | HSPG2 | 0.346032528 | -1.531020434 |
| ENSG00000170425 | ADORA2B | 0.345734809 | -1.53226223 |
| ENSG00000241360 | PDXP | 0.345040438 | -1.535162642 |
| ENSG00000224578 | HNRNPA1P48 | 0.34480481 | -1.536148196 |
| ENSG00000160781 | PAQR6 | 0.34466199 | -1.536745892 |
| ENSG00000258839 | MC1R | 0.344348426 | -1.538059013 |
| ENSG00000170421 | KRT8 | 0.342796753 | -1.544574652 |
| ENSG00000254870 | ATP6V1G2-DDX39B | 0.342332442 | -1.546530074 |
| ENSG00000134352 | IL6ST | 0.341644514 | -1.549432133 |
| ENSG00000172954 | LCLAT1 | 0.339228057 | -1.559672598 |
| ENSG00000196950 | SLC39A10 | 0.337808382 | -1.565722968 |
| ENSG00000137824 | RMDN3 | 0.33737753 | -1.567564202 |
| ENSG00000146587 | RBAK | 0.334776494 | -1.578729859 |
| ENSG00000126453 | BCL2L12 | 0.334073025 | -1.581764598 |
| ENSG00000152926 | ZNF117 | 0.333578147 | -1.583903314 |
| ENSG00000257341 | AL928654.3 | 0.333113624 | -1.585913736 |
| ENSG00000065485 | PDIA5 | 0.325358851 | -1.619896294 |
| ENSG00000283515 | AC020915.5 | 0.324192991 | -1.625075196 |
| ENSG00000167695 | FAM57A | 0.322201718 | -1.633963907 |
| ENSG00000079739 | PGM1 | 0.321357563 | -1.63774867 |
| ENSG00000139146 | SINHCAF | 0.320271077 | -1.642634577 |
| ENSG00000101945 | SUV39H1 | 0.319299849 | -1.647016225 |
| ENSG00000105323 | HNRNPUL1 | 0.318371545 | -1.651216695 |
| ENSG00000104341 | LAPTM4B | 0.313315137 | -1.674313624 |
| ENSG00000115107 | STEAP3 | 0.312864033 | -1.676392278 |
| ENSG00000182774 | RPS17 | 0.312110054 | -1.679873261 |
| ENSG00000112578 | BYSL | 0.30791315 | -1.699404615 |
| ENSG00000240038 | AMY2B | 0.307124397 | -1.703104974 |
| ENSG00000102225 | CDK16 | 0.306438837 | -1.706328942 |
| ENSG00000124172 | ATP5F1E | 0.300217575 | -1.735919661 |
| ENSG00000284862 | CCDC39 | 0.299982211 | -1.737051143 |
| ENSG00000105976 | MET | 0.293162127 | -1.770229356 |
| ENSG00000115762 | PLEKHB2 | 0.285342379 | -1.809234063 |
| ENSG00000123689 | G0S2 | 0.280367884 | -1.834606995 |
| ENSG00000178882 | RFLNA | 0.277374894 | -1.850090884 |
| ENSG00000122861 | PLAU | 0.270789047 | -1.884758708 |
| ENSG00000169908 | TM4SF1 | 0.270420376 | -1.886724233 |
| ENSG00000242265 | PEG10 | 0.270084552 | -1.888516968 |
| ENSG00000173674 | EIF1AX | 0.267902585 | -1.900219595 |
| ENSG00000083312 | TNPO1 | 0.267877178 | -1.90035642 |
| ENSG00000285625 | AC117378.1 | 0.263768622 | -1.922655141 |
| ENSG00000145730 | PAM | 0.262879385 | -1.927527086 |
| ENSG00000123179 | EBPL | 0.254440892 | -1.974597547 |
| ENSG00000259040 | BLOC1S5-TXNDC5 | 0.254078534 | -1.976653601 |
| ENSG00000270757 | HSPE1-MOB4 | 0.25363249 | -1.979188529 |
| ENSG00000071553 | ATP6AP1 | 0.252770827 | -1.984098127 |
| ENSG00000104131 | EIF3J | 0.25160604 | -1.99076154 |
| ENSG00000170545 | SMAGP | 0.244638782 | -2.031274967 |
| ENSG00000076554 | TPD52 | 0.238104529 | -2.070333032 |
| ENSG00000255439 | AC135050.2 | 0.231619917 | -2.110168777 |
| ENSG00000257524 | AL157935.2 | 0.228333584 | -2.130785025 |
| ENSG00000135486 | HNRNPA1 | 0.227330085 | -2.137139474 |
| ENSG00000103257 | SLC7A5 | 0.215983516 | -2.211006887 |
| ENSG00000269955 | FMC1-LUC7L2 | 0.20625127 | -2.277525094 |
| ENSG00000132383 | RPA1 | 0.202221681 | -2.305990412 |
| ENSG00000160888 | IER2 | 0.200852441 | -2.315792103 |
| ENSG00000254692 | AL136295.1 | 0.198261249 | -2.334525372 |
| ENSG00000118707 | TGIF2 | 0.18937146 | -2.400709173 |
| ENSG00000135678 | CPM | 0.186060049 | -2.426159783 |
| ENSG00000140988 | RPS2 | 0.184641995 | -2.437197377 |
| ENSG00000286235 | AL035461.3 | 0.165963755 | -2.591059892 |
| ENSG00000069702 | TGFBR3 | 0.163179669 | -2.615466777 |
| ENSG00000265590 | C21orf59-TCP10L | 0.162940912 | -2.617579208 |
| ENSG00000137801 | THBS1 | 0.158349091 | -2.65881951 |
| ENSG00000108825 | PTGES3L-AARSD1 | 0.155636247 | -2.683750003 |
| ENSG00000144959 | NCEH1 | 0.141944688 | -2.816599236 |
| ENSG00000110651 | CD81 | 0.13546065 | -2.884054267 |
| ENSG00000171155 | C1GALT1C1 | 0.134876424 | -2.890289905 |
| ENSG00000277957 | SENP3-EIF4A1 | 0.102574371 | -3.285257794 |
| ENSG00000288053 | AC231657.3 | 0.063343077 | -3.980669249 |
| ENSG00000255508 | AP002990.1 | 0.059683957 | -4.066513009 |
| ENSG00000285920 | AC087721.2 | 0.059674192 | -4.066749062 |
| ENSG00000255730 | AC011462.1 | 0.048302961 | -4.371744556 |
